# Synthesis, Characterization and Antimicrobial Activity of Trimethylantimony(V) Biscyanoximates, a New Family of Antimicrobials

**DOI:** 10.3390/molecules29235779

**Published:** 2024-12-06

**Authors:** Seth A. Amankrah, Tarosha Salpadoru, Kaitlyn Cotton, Marianna A. Patrauchan, Karen L. Wozniak, Nikolay Gerasimchuk

**Affiliations:** 1 Department of Chemistry and Biochemistry, Missouri State University, Springfield, MO 65897, USA; saduamankrah@gmail.com; 2 Department of Microbiology and Molecular Genetics, Oklahoma State University, Stillwater, OK 74078, USA; tarosha.salpadoru@okstate.edu (T.S.); kaicott@okstate.edu (K.C.); karen.wozniak@okstate.edu (K.L.W.)

**Keywords:** cyanoximes, trimethylantimony (V), X-ray analysis, thermal analysis, antimicrobial activity, antifungal activity

## Abstract

Antimicrobial compounds play a critical role in combating microbial infections. However, the emergence of antibiotic and antifungal resistance and the scarcity of new antibiotic developments pose a significant threat and demand the discovery of new antimicrobials for both bacterial and fungal pathogens. Our previous work described the first generation (***G1***) of organoantimony-based compounds that showed antimicrobial activity against several bacterial and fungal pathogens. Here, we present our efforts in modifying these compounds by replacing the tetraphenyl backbone in ***G1*** compounds with a trimethyl group, thereby generating a new series of compounds we refer to as “generation 2”, ***G2***. In addition to the novel backbone structure, we introduced three new anionic chloro-cyanoxime ligand groups, namely 2,4-diCl-PhCO^−^, 2,6-diCl-PhCO^−^ and 2Cl-PhCO^−^, which were found to be biologically active in the past. Nine new compounds of SbMe_3_L_2_ composition were obtained in high yields and characterized by NMR, IR spectroscopies, thermogravimetric TG/DSC and X-ray single crystal analyses. The antibacterial activity of the cyanoximates was tested against three bacterial (*Pseudomonas aeruginosa* PAO1, *Escherichia coli* S17 and methicillin-resistant *Staphylococcus aureus* (MRSA) NRS70) and two fungal (*Candida albicans* strain SC5314 and *Cryptococcus neoformans* strain H99) pathogens. Two compounds, SbMe_3_(MCO)_2_ and SbMe_3_(2,4-diClPhCO)_2_, were active against bacterial strains and inhibited the growth of PAO1 and MRSA with MICs of 50 and 100 µg/mL, respectively. Three compounds, SbMe_3_(MCO)_2_, SbMe_3_(ECO)_2_ and SbMe_3_(TCO)_2_, were active against fungal strains and inhibited either one of or both *C. albicans* and *C. neoformans* at MICs of 2.6–66.67 μg/mL. In addition, SbMe_3_(TCO)_2_ and SbMe_3_(MCO)_2_ were fungicidal at MFC 33.33–66.67 μg/mL. Ultra-thin-layer TEM imaging suggested that SbMe_3_(MCO)_2_ targets the integrity of bacterial membranes. Overall, four of the studied ***G2*** series compounds possess antimicrobial activity against a broad range of microbial pathogens, with particular potential against fungal pathogens, which will be explored in further studies.

## 1. Introduction

Antibiotics are considered one of the most important medical interventions that play a critical role in combating severe infections. Conceptually, they belong to a broader category, antimicrobials. While antibiotics are commonly defined as compounds targeting only bacteria, antimicrobials affect all microorganisms, including bacteria and fungi, and therefore have significant importance in medicine. However, the persistent challenges associated with their use over time have led to the emergence of antimicrobial resistance, posing a significant threat to the effectiveness of the treatments. In recognition, the World Health Organization (WHO) and the Center for Disease Control (CDC) have identified global priority pathogens that exhibit multidrug resistance (MDR) and emphasized the urgent requirement for the development of new antimicrobial agents [1,2]. Infections caused by MDR pathogens have led to higher mortality rates and are estimated to impose an economic burden of over USD 20 billion per year in the United States alone [3,4,5]. The WHO has listed bacterial priority pathogens that include several Gram-negative bacteria such as *Pseudomonas aeruginosa* and *Escherichia coli*, and Gram-positive bacteria such as methicillin-resistant *Staphylococcus aureus* (MRSA) [1,6]. In addition, the fungal pathogens *Candida albicans* and *Cryptococcus neoformans* were classified by the WHO as “critical priority” fungal pathogens in the 2022 report [7]. These pathogens have a remarkable track record of causing a significant number of infections that pose a threat to life. For example, in 2019, bacterial pathogens were responsible for an average of 3.57 million deaths, with MRSA alone causing more than 100,000 deaths [3]. From 2019 to 2021, fungal pathogens were responsible for an annual incidence of 6.5 million invasive fungal infections and 3.8 million annual deaths, with approximately 1 million annual deaths attributed to *Candida* bloodstream infections and 147,000 annual deaths attributed to cryptococcal meningitis [8]. Therefore, discovering new antimicrobials for both bacterial and fungal pathogens is of utmost importance. For this, inorganic compounds were found to be especially promising [9,10].

Metal-based coordination and organometallic compounds have been found to exhibit significant antimicrobial activity, often with a less targeted mode of action than traditional antibiotics. This reduces specific selection pressure, thereby lowering the likelihood of resistance development to these compounds. Numerous organotin (IV), silver (I) [11,12], copper (II) [13], zinc [14] and even mercury [15] and arsenic (III) compounds have been extensively studied as alternatives to antibiotics [16]. However, most of them are toxic to human cells, which limits their further development for biomedical applications.

Here, we focused on antimony, which is in the main Group 15 (pnictogens) in the Periodic Table. The pnictogens display broadly varying chemical and biological properties despite being elements of the same group. Thus, nitrogen and phosphorus are non-metals and are essential for life elements, arsenic and antimony are metalloids with the former being acutely toxic, and bismuth is a nontoxic metal widely used as bismuth (III) salicylate in a popular “five-in-one” drug sold as Pepto-Bismol© [17]. A brief description of inorganic antimicrobials, including antimonials, that are used in medical practice is provided in the Electronic Supplementary Materials section *ESI 1*. Antimony readily forms compounds with dominant oxidation states +3 and +5 and possesses a low general toxicity whilst demonstrating appreciable antimicrobial properties. A simple inorganic antimony compound, NaSbO_3_ (meta antimonate), was used as an emetic agent up until the late 1700s. There are two Sb-based carbohydrate compounds that were developed in the past as antimicrobial drugs—Pentostam [18] and Glucantime [19]. We aimed to use organometallic derivatives of antimony because Sb-C bonds are much more stable towards hydrolysis in aqueous media as opposed to Sb-N and Sb-O bonds. The latter causes fairly rapid biodegradation and inactivation of the above commercially used drugs. Further, there are relatively easily accessible phenyl- and methyl-derivatives of antimony with variable content of organic groups that can be obtained from inexpensive precursors such as SbCl_3_ and SbBr_3_ and a variety of Grignard reagents.

The acidoligands chosen to be bound to the metalloid were cyanoximes. This is a new subclass of oximes, small organic compounds representing weak acids. The general structure for cyanoximes is HO-N=C(CN)-R, where R represents the varying electron withdrawing group, typically O, N or S. The nitrile group (CN) makes the cyanoximes several orders of magnitude more acidic, which makes them better nucleophiles and, therefore, a better ligand when compared to other oximes [20]. There are 44 known cyanoximes today that have been characterized thus far, with many found to have significant biological activity. This ranges from growth regulation and detoxification to antimicrobial applications [21,22,23,24,25,26,27]. Our previous work detected antimicrobial activity in silver (I) cyanoximates and in compounds containing sulfur thioamide–cyanoxime, HO-N=C(CN) C(S)NH_2_ (later TCO). Currently, there are new antiviral oxime-containing compounds being developed that affect HIV, SARS-CoV-2 and MERS-CoV-2, all of which are now in different stages of clinical trials [28]. Finally, several tetraphenylantimony(V) compounds with oximes of substituted benzaldehydes have been made and characterized [29,30].

**Figure 1:**
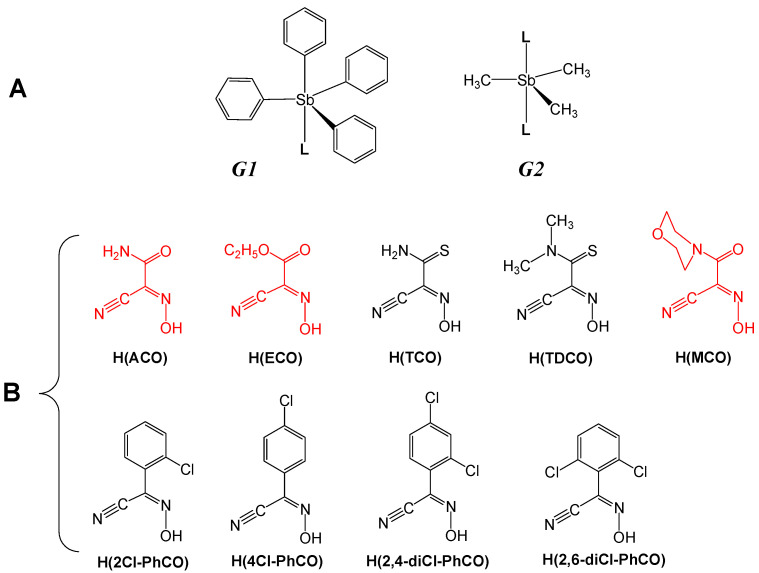
(**A**)—schematic depiction of the ***G1*** and ***G2*** families of organoantimony(V) cyanoximates where L is the cyanoxime anion; (**B**)—choice of ligands for the ***G2*** series. Highlighted in red are the most active compounds found in studies of the ***G1*** family of organoantimonials [34].

We aimed to combine the biological properties of cyanoximes with the low general toxicity of antimony to generate a library of compounds with potential antimicrobial activity for applications in the biomedical area. To the best of our knowledge, our previous work presents the only two publications reporting the synthesis, properties and biological activity of Sb-based compounds with oxime-bearing ligands [31,32]. In the latter paper, we reported the synthesis, structures and properties of the first generation (we designated this ***G1***) of the novel organoantimony cyanoximates SbPh_4_L based on a tetraphenyl-Sb(V) core, where L were specially chosen cyanoximes with previously established biological activity (Figure 1A). Considering the well-established importance of even small variations in the chemical structure, bulkiness and hydrophobicity of compounds for their biological activity [33,34,35,36,37,38,39,40] and based on our earlier data describing properties of the ***G1*** family of compounds, we propose to generate a novel ***G2*** series of compounds in which four bulky lipophilic phenyl groups are replaced with three compact methyl groups (Figure 1B). Also, there is an increase in cyanoxime moieties from one in the ***G1*** family to two in the new ***G2*** family. Thus, the number of hydrophobic phenyl groups in the core structure is progressively changed with the simultaneous increase in the number of the biologically active cyanoximes ACO^−^, ECO^−^, MCO^−^ or TCO^−^ from one to two. Varying the number of bulky lipophilic phenyl groups in combination with introducing different cyanoximes bound to the central Sb atom will allow us to identify the most optimal molecular design for the desired chemical stability and antimicrobial properties.

Here, we present the preparation and detailed characterization of the second generation, ***G2***, of the new SbMe_3_L_2_ organoantimonials based on a trimethyl-Sb(V) core with cyanoximes **L**, with some of them being selected as the most active compounds in the ***G1*** family. Their chemical structures are presented in Figure 1. Nine new compounds were obtained in high yield and characterized by NMR, IR spectroscopies, thermogravimetric TG/DSC and X-ray single crystal analyses. The antibacterial activity of the cyanoximates was tested against three bacterial (*Pseudomonas aeruginosa* PAO1, *Escherichia coli* S17 and methicillin-resistant *Staphylococcus aureus* (MRSA) NRS70) and two fungal (*Candida albicans* strain SC5314 and *Cryptococcus neoformans* strain H99) pathogens.

## 2. Results and Discussion

### 2.1. Chemistry

#### 2.1.1. Preparation of Compounds

Not one but two metal salts of Ag(I) and Tl(I) were successfully employed in the synthesis of the desired SbMe_3_L_2_ compounds for two reasons. The fact is that Ag(I) complexes for ACO^−^, ECO^−^ and MCO^−^ anions are very stable and light-insensitive [41] and represent very convenient sources of cyanoximes for a variety of organometallic derivatives. At the same time, Ag(I) salts of halogenated arylcyanoximes 2Cl-PhCO^−^, 4Cl-PhCO^−^, 2,4-diCl-PhCO^−^ and 2,6-diCl-PhCO^−^ are very light and temperature-sensitive, and products of their reactions are often contaminated with colloidal Ag as the result of photoreduction. Furthermore, Ag(I) complexes with thioamides TCO^−^ and TDCO^−^ do not exist because of the very rapid decomposition with the formation of Ag_2_S and elemental sulfur due to the oxidizing power of Ag(I). The use of Tl(I) offers an immense advantage in metathesis reactions because it chemically very much resembles silver(I) [42], but Tl-compounds are not light-sensitive and non-oxidizers, like Ag(I). As has been said, the metathesis reaction is heterogeneous, carried out between solid Ag(I) or Tl(I) cyanoximates and SbMe_3_Br_2_ in the solution, and was successfully carried out using an ultrasound bath to assure complete mixing of reactants in propionitrile. This solvent proved to be better than acetonitrile because it has a higher boiling point and is less volatile. This gave the following advantages: (1) a higher boiling point makes handling of solutions easier; (2) slower crystal growth provided better quality; and (3) it is miscible with pentane used for crystal growth using the vapor diffusion method. Also, six TlL compounds with four of the above halogenated aryl cyanoximes and two thioamide cyanoximes, TCO^−^ and TDCO^−^, were key precursors for trimethylantimony(V) compounds and were prepared following the published methods [34]. The crystal structures of three of them—Tl(4Cl-PhCO), Tl(2,4-diCl-PhCO) and Tl(2,6-diCl-PhCO)—were determined for the first time and are presented in *ESI 2*–*12*. By using the metathesis reaction (see Figure 8 in the Experimental Section), we generated very fine precipitates of AgBr or TlBr that are insoluble in propionitrile and are very difficult to filter using conventional filters. Further, passing the reaction mixture through Celite© created conditions for hydrolysis of the prepared organoantimonials since the surface of this solid phase contains numerous hydroxyl groups. Partially hydrolyzed organometallic antimony(V) compounds facilitate the formation of oxo-dimers, as we observed earlier in the case of isolation of [SbPh_4_(ECO)]_2_O [43]. Therefore, centrifugation of the reaction mixture was proved to be the ideal technique for separation of solid AgBr and TlBr. This method leaves transparent trimethylantimony(V) cyanoximates as a supernatant in the propionitrile solution. Large single crystals suitable for X-ray analysis were grown from these solutions for all of the organoantimonials reported in this work.

#### 2.1.2. Structures of Thimethyl-bis-cyanoximates of Sb(V)

**Scheme 1:**
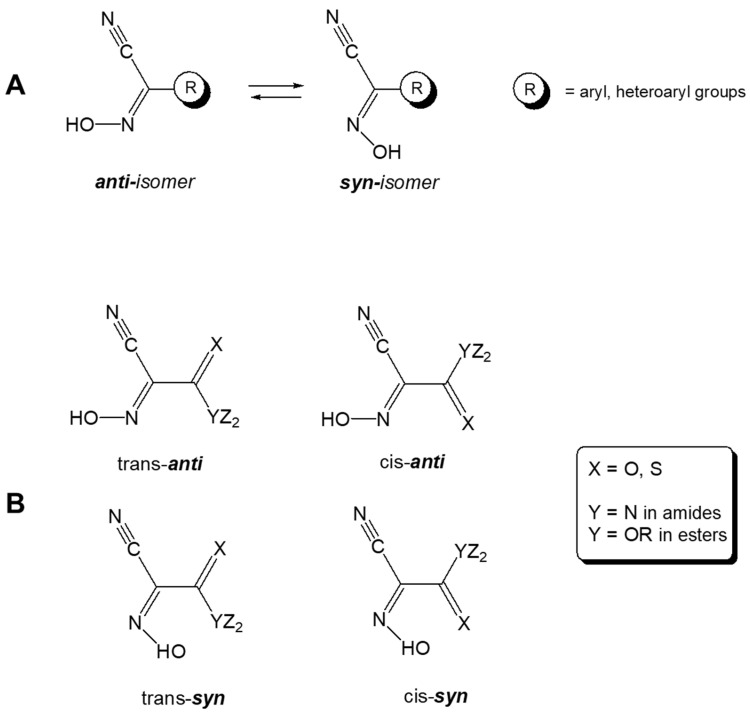
Principal geometrical stereoisomers of cyanoximes (**A**) and observed isomerism of protonated HL in amide-/ester-/keto-cyanoximes (**B**) [20].

**Scheme 2:**
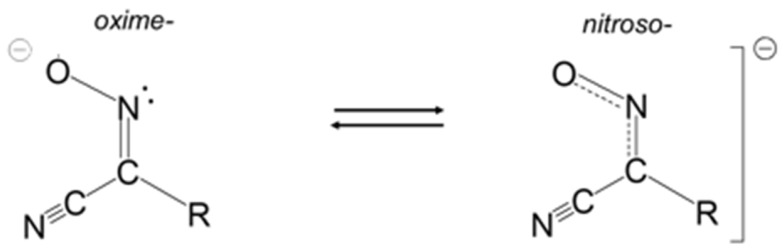
Redistribution of electron density in cyanoxime anions.

Cyanoximes contain a double bond in the >C=N-OH fragment that causes stereoisomerism, as explained in Scheme 1, while the relationship between *oxime* and *nitroso*-anions is shown in Scheme 2. Thus, during analysis of the structural chemistry of cyanoximes and their coordination and organometallic compounds, identification and presentation of both features become an important part of the characterization of novel compounds. This is specifically stated during the description of synthesized compounds.

**Figure 2:**
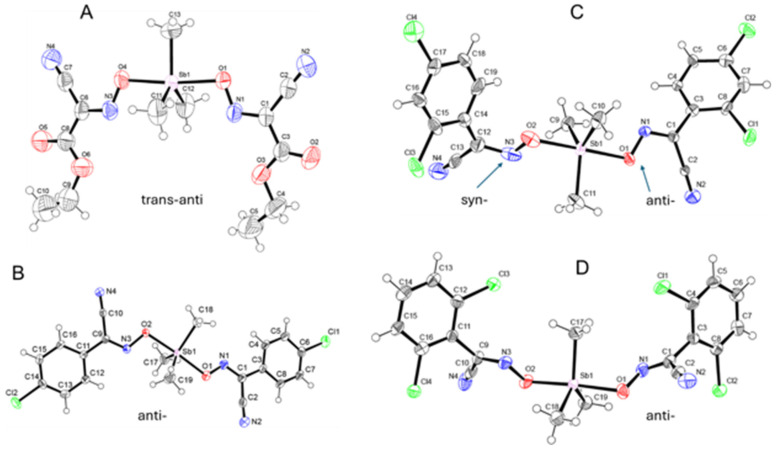
Molecular structures and numbering schemes of SbMe_3_L_2_ compounds with (**A**) ester-cyanoxime ECO^−^, and halogenated arylcyanoximes such as (**B**) 4Cl-PhCO^−^, (**C**) 2,4-diCl-PhCO^−^ and (**D**) 2,6-diCl-PhCO^−^. Assignment of geometry of anions is shown.

**Figure 3:**
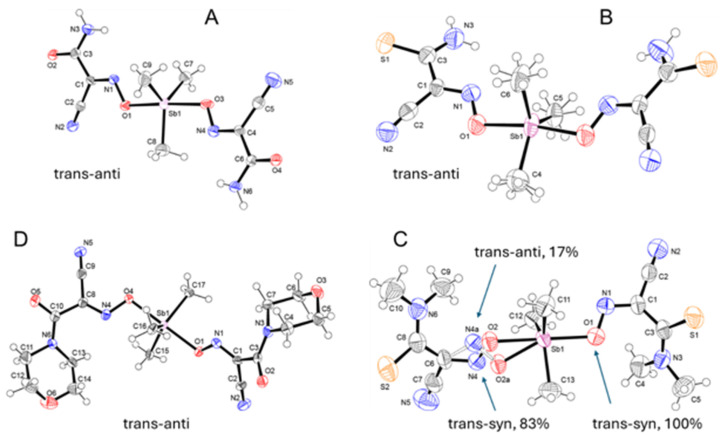
Molecular structures of trimetylantimony(V) bis-cyanoximates such as the amide/thioamide-cyanoximes: (**A**) ACO^−^, (**B**) TCO^−^, (**C**) MCO^−^ and (**D**) TDCO^−^. Geometry of O-bound anions is shown.

The vapor diffusion technique in propionitrile/pentane propionitrile/ether systems was successfully used for growing suitable crystals for structure determination. Crystallographic data for eight newly synthesized trimethylantimony(V) cyanoximates are shown in Table 1. The crystal structures of the ***G2*** series of SbMe_3_L_2_ cyanoximates are presented in Figure 2 and Figure 3 in an ORTEP representation at 50% thermal ellipsoid probability level. In all cases, the antimony(V) center adopts a slightly distorted trigonal bipyramidal environment with relatively compact methyl groups occupying the least sterically crowded equatorial space, with cyanoxime anions taking axial positions. Anions coordinated to Sb(V) centers are monodentate O-bound ligands. Therefore, the composition of the coordination polyhedron of the central atom in the ***G2*** series of organoantimonials is [SbC_3_O_2_] for all studied compounds. This is in line with our previous finding for the ***G1*** series of tetraphenyl cyanoximates having [SbC_4_O] as the composition of the coordination polyhedron [31,44]. The structural consistency throughout both series of novel organoantimony(V) cyanoximates is important, as the observed biological activity for compounds in the ***G1*** and ***G2*** series may be directly associated with the effect of coordinated cyanoximes.

Below, we present a brief discussion of the crystal structures of organoantimony(V) cyanoximates that were used for antimicrobial testing. Auxiliary crystal structures of Tl(4Cl-PhCO), Tl(2,4-diCl-PhCO) and Tl(2,6-diCl-PhCO) that were precursors to the desired respective SbMe_3_L_2_ haloaryl–cyanoxime compounds are presented in *ESI 2–12*. The preparation of thallium salts is given in *ESI 13*. As we pointed out already for these compounds, cyanoximes silver(I) complexes were either not obtainable or highly light-sensitive. Crystal data for other key precursors, such as Tl(TCO) and Tl(TDCO), are the subject of another paper and will be published elsewhere. These two Tl(I) compounds were used for the synthesis of SbMe_3_(TCO)_2_ and SbMe_3_(TDCO)_2_, which we present here.

**Crystal structure of SbMe_3_(ECO)_2_:** The molecular structure of this compound is shown in Figure 3A, while the selected bond lengths and angles can be found in *ESI 14*. Both anions adopt *trans*-*anti*-geometry and represent *oximes*, as can be judged from the averaged C-N bond (1.341 Å) that is shorter than the averaged N-O bond (1.292 Å) in the >C-N-O fragment. With respect to the O-Sb-O direction, both anions are located on the same side, forming a pseudo-cis position of cyanoximes (cisoid, Figure 2A). Two coordinated ECO^−^ anions have non-planar cores with different dihedral angles of 27.01° and 6.05° between the oxo fragment and the cyanoxime fragment. Crystal packing consists of puckered sheets stacked on top of each other with weak electrostatic forces and van-der-Waals forces connecting them.

**Crystal structure of SbMe_3_(4-Cl-PhCO)_2_:** The asymmetric unit (ASU) for this compound is shown in Figure 2B, and the selected bond lengths and valence angles are summarized in *ESI 15*. Both anions within SbMe_3_(4-Cl-PhCO)_2_ adopt an *anti*-geometry (Scheme 1A) and represent *nitroso* ligands (Scheme 2), as is evident from the shorter averaged N-O bonds as compared to C-N in the fragment. Surprisingly, contrary to the structure of the starting SbMe_3_Br_2_, all the methyl groups attached to the central atom in this compound were slightly different from each other in bond lengths and attachment angles. Also, it was interesting to observe that the two (4-Cl-PhCO)^−^ anions possess different planarity. Molecules in the structure are held together by π-π interactions between aromatic groups of neighboring units and van-der-Waals forces.

**Crystal structure of SbMe_3_(2,4-diCl-PhCO)_2_**: The molecular structure of SbMe_3_(2,4-diCl-PhCO)_2_ is shown in Figure 2C, while the selected bond length and angles are presented in *ESI 16*. This structure is truly unique as it has not been previously detected in any other cyanoximes or their numerous complexes with a variety of metals. There is a 1:1 ratio of *syn*- and *anti*-diastereomers of the same cyanoxime anion in one structure, within the same molecule. Previously, we observed quite rare co-crystallization of these two diastereomers in the ASU of some structures [45], but all of them were at different (non-equal) ratios. The uniqueness of this situation is that one of the two anions adopts *syn*-geometry, while the second one has *anti*-geometry (Scheme 1). Thus, for the first time, we found a case of co-crystallization of two diastereomers at exactly a 1:1 ratio. It is interesting to note that in the structure of the Tl(2,4-diCl-PhCO) precursor, only the *anti*-stereoisomer was observed (*ESI 5*–*8*). The Sb-C bond lengths of the CH_3_-group carbon atoms attached to the metal Sb center are all different. Both cyanoximes coordinated to the metalloid appear heavily non-planar and exhibit significant dihedral angles between cyanoxime and chloroaryl fragments. Hence, the dihedral angle between the mean planes N2-C2-C1-N1-O1 and the Cl-Ph-group is 53.3° for the *syn*-stereoisomer (Figure 3C). The same dihedral angle between the planes O2-N3-C9-C10-N4 and the Cl-Ph-group is 39.2° for the *anti*-stereoisomer. Another interesting peculiarity is that in the *anti*-isomer, an anion is an *oxime* with the N-O bond longer than C-N, while in the *syn*-isomer it is a *nitroso* ligand as the C-N bond is longer and N-O is shorter (*ESI 16*). Interestingly enough, the Sb1-O bonds are also significantly different: 2.203 Å for *syn*- and 2.086 Å for *anti*-stereoisomers. There are no hydrogen bonds present, and the crystal lattice is stabilized by π-π stacking interactions between the aryl groups from neighboring units and weak C-H---Cl inter- and intramolecular forces.

**Crystal structure of SbMe_3_(2,6-diCl-PhCO)_2_**: The molecular structure of this compound is presented in Figure 2D. Cyanoxime moieties within the structure adopt an *anti*-geometry like their Tl precursor Tl(2,6-diCl-PhCO) (*ESI 9*–*12*). Selected bond lengths and valence angles for SbMe_3_(2,6-diCl-PhCO)_2_ are presented *ESI 17*. The molecular structure of this compound resembles the cisoid arrangement for SbMe_3_(ECO)_2_: both anions are on one side of the O-Sb-O line (Figure 2D). As expected, both cyanoxime anions in the structure of SbMe_3_(2,6-diCl-PhCO)_2_ are non-planar. The dihedral angles between the cyanoxime group and the disubstituted aryl group are 82.5° for mean planes between the Ph group and O2-N3-C9-C10-N4, and 69.7° for mean planes between the Ph and O1-N1-C1-C2-N2 fragment. These angles are larger than the dihedral angle in the thallium(I) precursor because there are bulkier methyl groups close to cyanoxime in SbMe_3_(2,6-diCl-PhCO)_2_ than in Tl(2,6-diCl-PhCO). Both anions represent *oximes*: N1-O1 = 1.359(7) Å, N3-O2 = 1.354(7) Å, N1-C1 = 1.282(8) Å and N3-C9 = 1.273(8) Å (*ESI 11*). Slipped π-π stacking interactions are present to stabilize the crystal structure.

**Crystal structure of SbMe_3_(ACO)_2_**: The molecular structure can be seen in Figure 3A. Selected bond lengths and valence angles are shown in *ESI 18*. There are two non-identical cyanoximes contrary to the thio-analog in the structure of SbMe_3_(TCO)_2_ (Figure 3B). Both cyanoxime moieties represent only one diastereomer with *trans*-*anti*-geometry and are located on opposite sides of the O-Sb-O line, adopting the overall transoid structure. In this compound, both anions are oximes and practically planar, with the values of dihedral angles between the cyanoxime core and amide group being less than 10°. The NH_2_ hydrogen atoms from the amide group form intermolecular H-bonding in this structure. Intermolecular electrostatic attractions and van-der-Waals contacts help further stabilize the crystal structure of this compound.

**Crystal structure of SbMe_3_(TCO)_2_:** The molecule of SbMe_3_(TCO)_2_ with the numbering scheme in the GROW view is presented in Figure 3B. The compound crystallizes in an orthorhombic space group with the Sb atom in a special position (mirror + gliding planes) and, therefore, has only one unique anion in the ASU. Selected bond lengths and valence angles can be found in *ESI 19*. Methyl groups on the metalloid atom are disordered by two positions. The anion represents practically planar oxime as a *trans*-*anti* diastereomer in this structure. Both TCO^−^ moieties are located on one side of the O-Sb-O line, and that justifies their cisoid arrangement at the Sb center. Intermolecular H-bonds are important for the lattice formation. Parameters for the hydrogen bonds are N3-H3B---N2 = 2.19 Å and H3B---N2 = 3.016(12) Å with <DHA = 161.8°. Weak electrostatic forces as well as van-der-Waals forces further stabilize the crystal structure.

**Figure 4:**
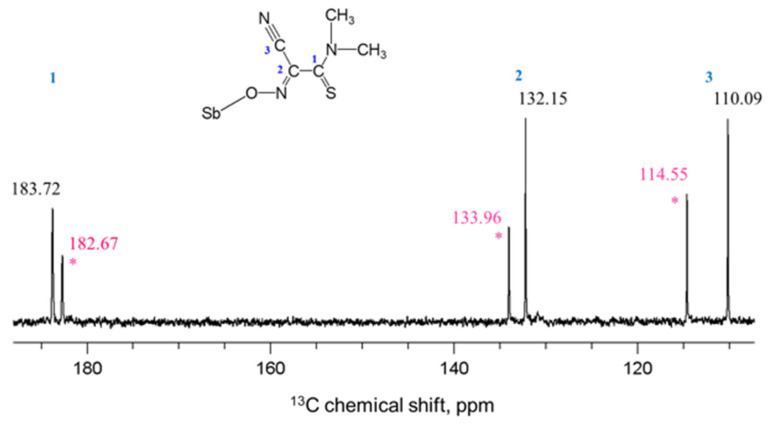
Fragment of ^13^C{^1^H} NMR spectrum of SbMe_3_(TDCO)_2_ in *sp*^2^ carbon atom region in DMSO-d_6_ at room temperature. Red asterisks and numbers show signals of minor *cis*-anti stereoisomer.

**Crystal structure of SbMe_3_(TDCO)_2_:** Selected bond lengths and valence angles for this compound are summarized in *ESI 20*. The molecular structure of this organoantimonial(V) compound is quite unique and shows the co-crystallization of two *syn*- and *anti*-diastereomers of the cyanoxime in one ASU in the structure (Figure 3C). Thus, there are unexpectedly high amounts of syn-isomer that were generated initially during the synthesis of H(TDCO) itself and then during the synthesis of the starting Tl(I) complex, from which SbMe_3_(TDCO)_2_ was obtained. This phenomenon is novel and has not been observed in the chemistry of cyanoximes before. The main peculiarity is the following: one TDCO^−^ anion is present as only a *syn*-diastereomer, while the second cyanoxime in the compound is present as both *syn*- and *anti*-diastereomers. The *syn*-isomer herein is still dominant with 84% occurrence, while the *anti*-isomer is minor with about a 16% contribution. This distribution of isomers was confirmed by the NMR ^13^C{^1^H} data displayed in Figure 4. Two syn TDCO^−^ acidoligands represent *oximes*, while the minor co-crystallized anti component is a *nitroso* ligand. Obviously, H(TDCO) itself is non-planar, and no expectation of planarity can be made for its Sb(V) complex either. Indeed, in the structures of SbMe_3_(TDCO)_2_, there are planes of the cyanoxime core and the thioamide group that have dihedral angles of 57.9° between the O1-N1-C1-C2-N2 and S1-C3-N3-C5 planes for the *syn*-isomer, and 58° for O2A-N4A-C6-C7-N5 and S2-C8-N6-C9 for the *anti*-isomer. The two TDCO^−^ anions are on opposite sides of the O-Sb-O line and can be assigned to a transoid arrangement in the ASU (Figure 3C). Only Van-der-Waals forces and weak C-H---NC- electrostatic interactions are responsible for crystal packing of this compound in the crystal lattice.

**Crystal structure of SbMe_3_(MCO)_2_:** The molecular structure of this compound is shown in Figure 3D. Two cyanoxime anions have the structure of only one trans-anti diastereomer (Scheme 1A). Selected bond lengths and valence angles are given in *ESI 21*. The six-membered rings of the morpholyl group adopt a more favorable chair conformation. Two MCO^-^ anions in the compound represent oximes since the N-O bonds are longer than the C-N in the >C-N-O fragment. The dihedral angle between the mean planes for the cyanoxime core O4-N4-C8-C9-N5 and the N6-C10-O5 amide plane is 31.5° for one anion and 44.2° for the same set of planes in the second anion.

Lastly, all crystal structures of the G2 family of novel organoantimonials depicted here represent slightly distorted trigonal bipyramids of [SbC_3_O_2_] composition with monodentate bound cyanoximes taking axial positions. There are small deviations from a perfect value of 180° in the O-Sb-O angle: O1-Sb1-O8 = 175.95° in SbMe_3_(ACO)_2_; O1-Sb1-O2 = 175.64° in SbMe_3_(2,4-diCl-PhCO)_2_; O1-Sb1-O2 = 175.48° in SbMe_3_(4Cl-PhCO)_2_; O1-Sb1-O4 = 174.07° in SbMe_3_(ECO)_2_; O1-Sb-O1* = 169.54° in SbMe_3_(TCO)_2_; O1-Sb1-O2 = 174.24° in SbMe_3_(TDCO)_2_; O1-Sb1-O4 = 175.87° in SbMe_3_(MCO)_2_; and O1-Sb1-O4 = 175.53° in SbMe_3_(2,6-diCl-PhCO)_2_. These deviations can be explained as the result of steric repulsion between bulky cyanoxime moieties and equatorial methyl groups. The average Sb-O bond, based on 15 data from eight structures, is 2.124 ± 0.029 Å, which is a normal value for a covalent single bond between these elements.

#### 2.1.3. Thermal Properties

**Figure 5:**
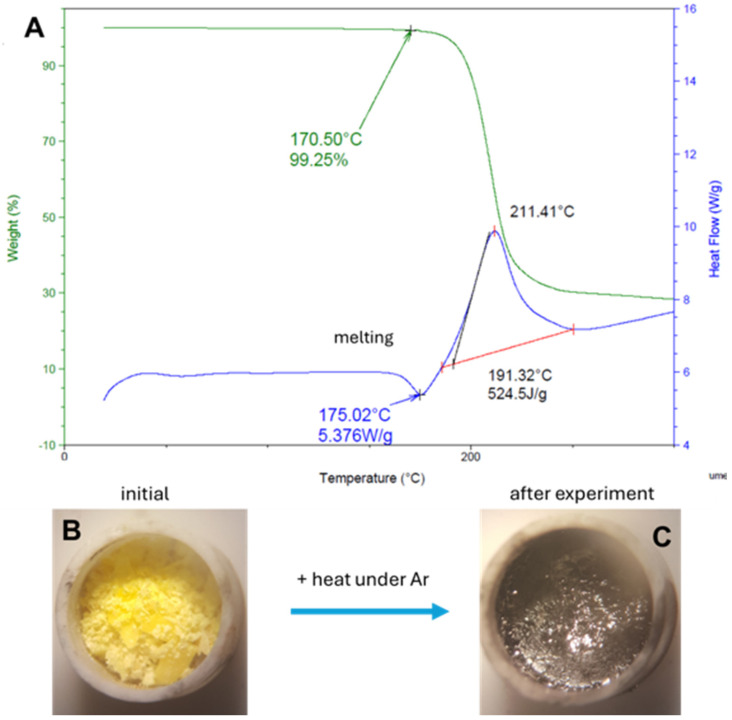
Thermal properties of SbMe_3_(TDCO)_2_: (**A**)—fragment of TG/DSC traces with clear melting at 175 °C while green arrow indicates point of crucible response on melting; (**B**)—yellow initial powder of the compound; (**C**)—final crucible content with sheets of elemental Sb.

**Figure 6:**
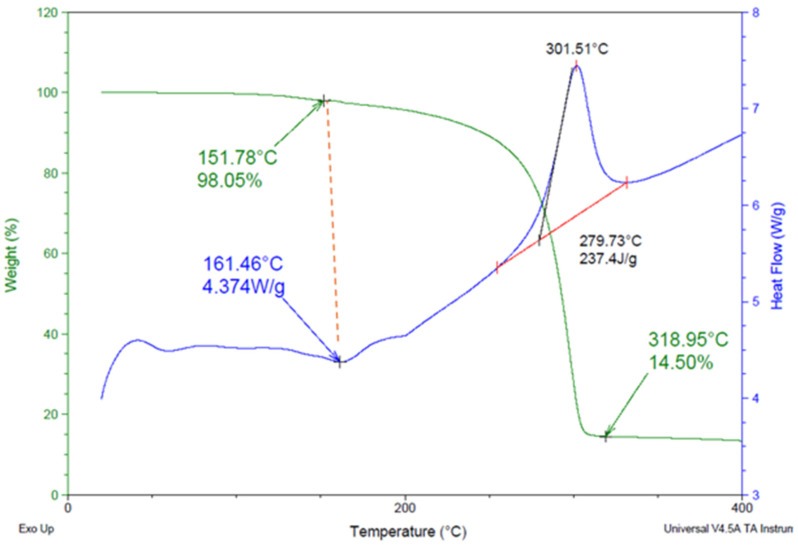
Fragment of TG/DSC traces for SbMe_3_(4Cl-PhCO)_2_ showing clear melting at 161.46 °C followed by decomposition after ~190 °C. Green arrow shows crucible weight response at beginning of melting.

All the thermal data for the cyanoximes HL and new organoantimony(V) cyanoximnates of the ***G2*** family were obtained by using the TG/DSC method. The method not only allowed accurate determination of the phase transition temperatures (melting), but also provided a good assessment of each compound’s thermal stability in the solid state. This is important from the standpoint of the intended practical application of the new compounds as antimicrobial agents either as stand-alone drugs or as components of coating composites with coated surfaces where sterilization is required and can be achieved by heating to ~110 °C. The results showed considerable thermal stability of solid samples of pure trimethylantimony(V) cyanoximates extending to at least 160 °C, which is sufficient for their thermal sterilization without decomposition. Selected examples are presented in Figure 5 and Figure 6. All the studied compounds rapidly decomposed with a strong exo-effect. Thus, peak integration for the 525.5 J/g heat flow of SbMe_3_(TDCO)_2_ corresponds to the 252 kJ/mol heat effect (Figure 5A). Similarly, the 237.4 J/g integrated heat flow peak corresponds to 141 kJ/mol of heat evolution for SbMe_3_(2,6-diCl-PhCO)_2_ (Figure 6). The final product of anaerobic thermal decomposition of the ***G2*** family of organoantimonials is elemental Sb in the form of thin sheets (Figure 5C). However, surprisingly, the residual amount of solid antimony in the crucible was considerably smaller than expected, and therefore, this suggests the formation of volatile products upon thermal degradation which leave the thermal analyzer with a flow of an inert purging gas. Similar observations were made for other SbMe_3_L_2_ compounds (*ESI 22* and *23*).

### 2.2. Microbiology

Our previous work described the ***G1*** generation of organoantimony-based compounds that showed antimicrobial activity against the bacterial pathogens *P. aeruginosa*, *E. coli* and *S. aureus* [31]. Here, we tested the antimicrobial activity of a new series of ***G2*** compounds that, in addition to the novel backbone structure, contained three new anionic cyanoxime ligand groups, namely 2,4-diCl-PhCO^−^, 2,6-diCl-PhCO^−^ and 2Cl-PhCO^−^.

**The novel organoantimony trimethyl cyanoximates SbMe_3_MCO and SbMe_3_(2,4-diClPhCO)_2_ inhibit bacterial and fungal growth.** We evaluated the antibacterial activity of seven trimethyl organoantimony compounds that comprised ***G2*** by standard methods. Our results showed that two of these compounds, SbMe_3_(MCO)_2_ and SbMe_3_(2,4-diClPhCO)_2_, inhibited the growth of *P. aeruginosa* and MRSA with MIC values of 50 and 100 µg/mL, respectively (Table 2). The ligand control of SbMe_3_(MCO)_2_, H(MCO), did not inhibit the growth of *P. aeruginosa* at the concentrations tested, indicating that the organoantimony group coupled to the novel ***G2*** backbone is primarily responsible for the antibacterial activity observed for SbMe_3_(MCO)_2_. This is, however, not true for SbMe_3_(2,4-diClPhCO)_2_, where the ligand control 2,4-diClPhCO^−^ alone inhibited MRSA, suggesting that this ligand group contributes to the antibacterial activity of SbMe_3_(2,4-diClPhCO)_2_ against MRSA (Table 2).

In comparison to its ***G1*** counterpart SbPh_4_MCO (MIC: 200 µg/mL) [31], SbMe_3_(MCO)_2_ inhibited *P. aeruginosa* with four-fold reduction in the MIC (Table 2). This indicates that introduction of the trimethyl backbone successfully improved the antibacterial activity of this compound against *P. aeruginosa.*

To our surprise, none of the new compounds showed antibacterial activity towards *E. coli.* It is noteworthy that our cumulative efforts focused on the ***G1*** and ***G2*** compounds identified only one compound, SbPh_4_ACO (from ***G1***), with inhibitory activity towards *E. coli* [31]. It is possible that *E. coli* may harbor mechanisms different from those in *P. aeruginosa* and MRSA that prevent the entry and/or the activity of these compounds.

Further, when tested against fungal pathogens, several of the ***G2*** compounds had antifungal activity, with some also having fungicidal activity (killing the pathogens rather than just inhibiting growth). SbMe_3_(MCO)_2_ was significantly effective against *C. albicans* and *C. neoformans* at concentrations of 25 μg/mL and 2.6 μg/mL, respectively. In addition, this compound was fungicidal against both *C. albicans* and *C. neoformans* at concentrations of 66.67 μg/mL and 33.33 μg/mL, respectively. SbMe_3_(ECO)_2_ was significantly effective against *C. neoformans* at 20.83 μg/mL. SbMe_3_(TCO)_2_ was significantly effective against *C. albicans* and *C. neoformans* at 50 μg/mL and 12.5μg/mL, respectively, but was only fungicidal for *C. neoformans* (Table 3). In addition, this compound was fungicidal for *C. neoformans* at 50 μg/mL. It is noteworthy that the ligand controls H(2,4-diCl-PhCO) and H(2-ClPhCO) were both antifungal towards *C. neoformans* at 25 μg/mL, indicating their significant contribution to the antimicrobial activity of the corresponding antimonial compounds. Interestingly, the compounds were much more effective against fungal organisms compared to bacterial organisms. For example, SbMe_3_(MCO)_2_ was effective against *S. aureus* and also against both fungal pathogens, but the MIC for fungal pathogens was much lower—25 µg/mL and 2.6 µg/mL for *C. albicans* and *C. neoformans*, respectively, compared to 50 µg/mL for *S. aureus*. In addition, there were compounds that showed efficacy against fungal organisms but not against bacterial organisms, including SbMe_3_(TCO)_2_ for both *C. albicans* and *C. neoformans* and SbMe_3_(ECO)_2_ against *C. neoformans*. This could indicate that the design of the ***G2*** series of compounds is more favorable towards fungal membranes and/or fungal cell walls compared to bacterial membranes and cell walls. Further studies will examine the mechanism(s) of antifungal activity of these compounds.

**Figure 7:**
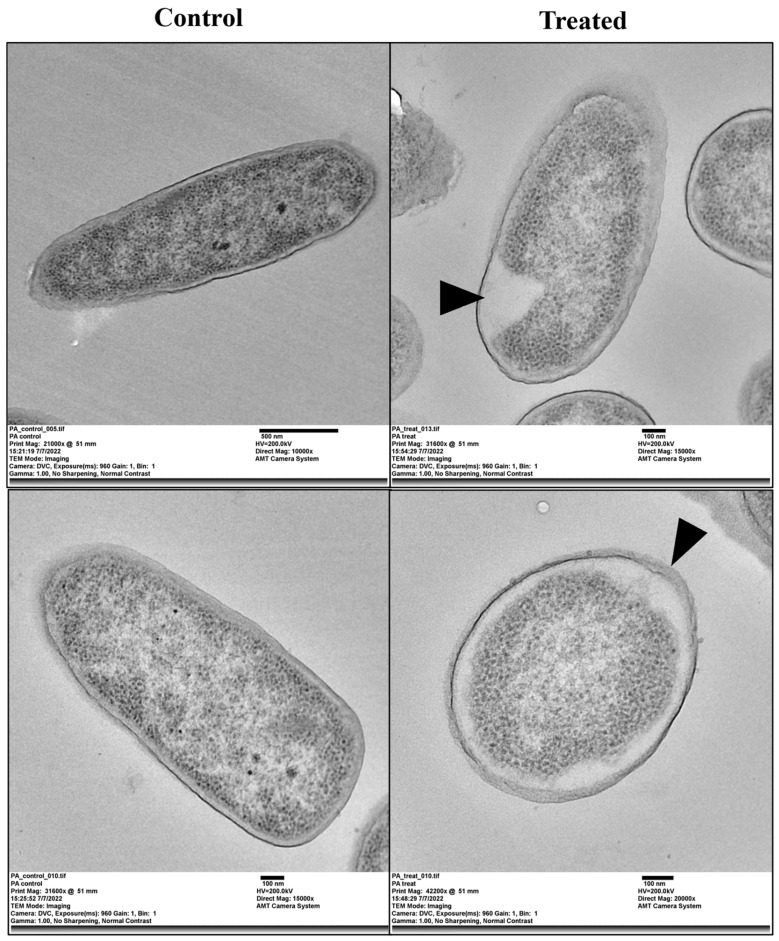
Representative ultra-thin TEM images of *P. aeruginosa* PAO1 treated with SbMe_3_(MCO)_2_ at the MIC or the solvent control, DMSO, for 1 h at 37 °C. The black arrows point to the separation of the cytoplasmic material from the cellular envelope observed upon treatment. In contrast, fully intact cells were observed in the control DMSO-treated group.

**SbMe_3_(MCO)_2_ causes disruptions in *P. aeruginosa* membranes.** To better understand the effects of the novel compounds on bacterial cells, we examined the morphological changes in *P. aeruginosa* treated with SbMe_3_(MCO)_2_ at its MIC for 1 h by using ultra-thin-layer TEM imaging. According to our observations, cells treated with 50 µg/mL SbMe_3_(MCO)_2_ showed prominent changes in cell morphology and structure in comparison to cells exposed to the solvent control, DMSO. In these samples, a distortion of the cell membranes and separation from the cytoplasm were observed (Figure 7). Some cells were also observed to lose their rod shape.

These results agree with our previous observations for the ***G1*** compound SbPh_4_ACO, suggesting that both types of compounds share the same mechanism of action and target the integrity of bacterial membranes. However, further validation is needed. Similar membrane disruptions have been observed in bacteria treated with silver-containing compounds [46]. This suggests a potentially shared property of metal-based compounds compromising membrane integrity that may help potentiate the efficacy of antibiotics by allowing their entry into bacterial cells. Since the outer membrane acts as a barrier for several classes of antibiotics, administering antimicrobial cocktails including these novel compounds with the ability to overcome this barrier may help expand the currently available treatment options for bacterial and fungal infections. However, further studies are needed to increase the efficacy of the compounds and evaluate their cytotoxicity against mammalian cells.

## 3. Experimental Section

### 3.1. Chemistry

**General information and physical methods:** All common chemicals and solvents used in this work were obtained from commercial sources (Aldrich, Strem, Across, St. Louis, MO, USA) and used without extra purification. Solvents were kept dry under freshly heat-activated molecular sieves. The antimony bis-cyanoximate SbMe_3_Br_2_ described herein is not commercially available. It was obtained from SbCl_3_, CH_3_-Mg-I and then Br_2_ using a previously described method [47]. All synthesized novel compounds were characterized by elemental analysis (C, H, N and (in some cases) S) using the combustion method (Atlantic Microlab, Norcross, GA, USA) displayed in *ESI 24*. Conventional spectroscopic methods were used as well: IR (Bruker Alfa ATR FT-IR spectrometer, Bruker, Massachussets, USA; 4 cm^−1^ resolution; 128 scans at room temperature), ^1^H, ^13^C{^1^H} NMR spectroscopy (Varian and Bruker 400 MHz spectrometers, Bruker, Massachussets, USA; room temperature, in DMSO-d_6_) and TG/DSC thermal analysis (TA Q-600; in the 25–800 °C range at 10°/min heat rate, under argon gas).

**X-ray analysis**: The crystallographic study was carried out using a Bruker APEX2 diffractometer equipped with a CCD camera, with Mo or Cu radiation sources, at cold or room temperatures depending on the thermomechanical stability of the selected crystals to prevent their cracking. Eight crystal structures were determined for almost all synthesized SbMe_3_L_2_ compounds (L = cyanoximes shown in Figure 1). In addition, we redetermined one crystal structure of the starting compound SbMe_3_Br_2_ and three crystal structures of the new auxiliary Tl(I) complexes Tl(4Cl-PhCO), Tl(2,4-diCl-PhCO) and Tl(2,6-diCl-PhCO) from which the respective trimethylantimony(V) compounds were obtained. Data for these three important precursor complexes are presented in *ESI 2*–*12*. The crystal structure of Tl(2Cl-PhCO) was reported earlier [48]. All data were collected with a 0.5° step and four omega runs of 364 frames each, covering a full sphere of reflections in 1456 frames. The H atoms in two structures were found objectively and refined, while in other structures, they were placed in geometrically idealized positions, based on hosting C and N atoms, and refined isotropically. Further, we redetermined the crystal structure of the Sb-source compound—trimethylantimony(V) dibromide (*ESI 25*–*27*)—and acquired better-quality data that were also deposited to the CCDC. All crystal structures reported in the main part were of good quality, and their solution/refinement did not present difficulties or errors. In some cases, it was possible to perform face indexing of crystals, which was helpful for a proper absorption correction procedure using the SADABS program. The video microscope photos of the obtained crystals are displayed in *ESI 28*. Crystal and refinement data are summarized in Table 1, while selected bond lengths and angles are presented in *ESI* pages 14–21. The ASU content drawings were made using Ortep 3.v2 software [49,50], whilst some details of the geometry of the reported compounds shown in the *ESI* section were made using the Mercury [51] package (version: 1.4.2). The determined crystal structures of organoantimonial cyanoximates were submitted to the CCDC and received registration numbers: SbMe_3_(ECO)_2_—2376857, SbMe_3_(MCO)_2_—2376859, SbMe_3_(ACO)_2_—2376862, SbMe_3_(4Cl-PhCO)_2_—2376858, SbMe_3_(2,4-diCl-PhCO)_2_—2376861 and SbMe_3_(2,6-diCl-PhCO)_2_—2376863. Moreover, we determined three crystal structures of the thallium(I) cyanoximates Tl(4Cl-PhCO), Tl(2,4-diCl-PhCO) and Tl(2,6-diCl-PhCO), which were used as precursors for the preparation of respective trimethylantimony(V) cyanoximates, as shown in Figure 2. Their CCDC registration numbers are as follows: Tl(4Cl-PhCO)—2380468, Tl(2,4-diCl-PhCO)—2380467 and Tl(2,6-diCl-PhCO)—2380469. Check CIF reports for all of them are presented at the very end of the Supplementary Materials section as multiple pages in *ESI 29* and *30*.

#### Preparation of Antimony Cyanoximates Using Metathesis Reaction

**Figure 8:**
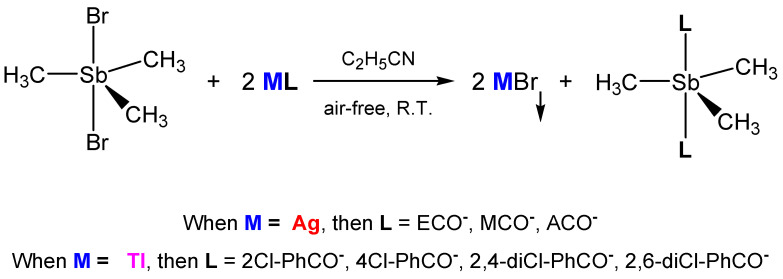
General route for preparation of antimony cyanoximates using the heterogeneous metathesis reaction.

Trimethylantimony(V) dibromide is soluble in practically all organic solvents and was used in the heterogeneous metathesis reaction for making organometallic cyanoximates from either Ag(I) or Tl(I) salts (Figure 8). Details of the particular use of each of these metal salts are explained in the Section 2 and in *ESI 31* and *32*. One of the typical preparations is presented below in detail, whilst for the other synthesized compounds only the amounts of the compounds used for their synthesis and a brief characterization of the obtained SbMe_3_L_2_ are shown. The data of the elemental analyses proving their composition can be found in *ESI 24*.

Trimethylantimony(V) dibromide was dissolved in ~10 mL of propionitrile. The solid AgL or TlL was then added in slow portions carefully to the solution under stirring on an ultrasound bath. This step is crucial because of the nature of the heterogeneous reaction where proper mixing of reactants is necessary. There was a color change from yellow to colorless/clear for all cyanoximes except TCO^−^ and TDCO^−^, which became yellow. The reaction was fast, and after ~10 min, the very fine white precipitates of AgBr or TlBr were separated via centrifugation. Complete separation required 3 min using the Thermo-Scientific Sorvall Legends Micro 17 (Thermo-Scientific, Waltham, WA, USA) at 10,000 rpm (*ESI 31*). After centrifugation, the supernatant now containing the solution of trimethylantimony(V) cyanoximate was transferred into a small beaker for subsequent crystallization in a desiccator charged with paraffin to help to absorb hydrocarbons such as propionitrile.

***SbMe_3_(ACO)_2_***: Amounts of 0.2000 g (0.6122 mmol) of SbMe_3_Br_2_ and 0.2693 g (1.224 mmol) of Ag(ACO) were used in the metathesis reaction (Figure 2). The yield of the dry product was 0.0250 g (10.4%) based on SbMe_3_Br_2_. IR spectrum, cm^−1^: 3406 ρ(NH_2_); 2935 ν(C-H); 2228 ν(C≡N); 1684 ν(C=O), 1600 ν(C=N); 1423, 1312 δ(C-H); 981 ν(N-O); 587 ν^as^(C-Sb).

***SbMe_3_(ECO)_2_*:** Amounts of 0.2000 g (0.6122 mmol) of SbMe_3_Br_2_ and 0.3049 g (1.225 mmol) of Ag(ECO) were used in the metathesis reaction. The yield of the dry product was 0.2220 g (80.8%) based on SbMe_3_Br_2_. The melting point was 140.52 °C. ^1^H NMR spectrum in DMSO-d_6_, ppm: 4.31 (2H, multiplet, CH_2_-group ECO^−^), 1.89, 1.71 and 1.67 (3H, singlet, CH_3_-group Sb), 1.28 (3H, multiplet, CH_3_-group ECO^−^). ^13^C{^1^H} NMR spectrum in DMSO-d_6_, ppm: 159, 127 (oxime carbons), 109.59 (CN-group), 62.08 (CH_2_-group ECO^−^), 14.47 (CH_3_-group ECO^−^), 8.33, 7.10, 6.63 (CH_3_-group Sb). IR spectrum, cm^−1^: 2996, 2967, 2940 ν(C-H); 2230 ν(C≡N); 1716 ν(C=O); 1465, 1372 δ(C-H); 1060 ν(N-O); 583 ν^as^(C-Sb).

***SbMe_3_(MCO)_2_***: Amounts of 0.1636 g (0.5008 mmol) of SbMe_3_Br_2_ and 0.2922 g (1.008 mmol) of Ag(MCO) were used in the metathesis reaction. The yield of the dry product was 0.1023 g (77.3%) based on SbMe_3_Br_2_. The melting point range was from 118.5 to 120 °C. IR spectrum, cm^−1^: 2965, 2935 ν(C-H); 2224 ν(C≡N); 1634 ν(C=O); 1460, 1390 δ(C-H); 930 ν(N-O); 589 ν^as^ (C-Sb).

**Synthesis of *SbMe_3_(4Cl-PhCO)_2_***: Amounts of 0.1700 g (0.5204 mmol) of SbMe_3_Br_2_ and 0.4000 g (1.042 mmol) of Tl(4-Cl-PhCO) were used in the metathesis reaction (Figure 2). The yield of the dry product was 0.1211 g (44%) based on the initial SbMe_3_Br_2_. The melting point was 162.27 °C (according to the TG/DSC method). IR spectrum, cm^−1^: 3072, 2966, 2932 ν(C-H) aromatic, aliphatic; 2220 ν(C≡N); 1594 ν(C=N); 1489 ν(C=C); 1489, 1397, 821 δ(C-H); 972 ν(N-O); 710 ν(C-Cl); 544 ν^as^(C-Sb).

Preparation **of *SbMe_3_(2Cl-PhCO)_2_*** was accomplished in the same manner.

***SbMe_3_(2,4-diCl-PhCO)_2_***: Amounts of 0.1570 g (0.4806 mmol) of SbMe_3_Br_2_ and 0.4000 g (0.9560 mmol) of Tl(2,4-diCl-PhCO) were used in the metathesis reaction. The yield of the dry product was 0.2130 g (74%) based on the initial antimony-source SbMe_3_Br_2_. The melting point was 129.18 °C. IR spectrum, cm^−1^: 3087 ν(C-H); 2219 ν(C≡N); 1585 ν(C=N); 1498 ν(C=C); 1379, 851 δ(C-H); 979 ν(N-O); 821 ν(C-Cl); 570 ν^as^ (C-Sb).

***SbMe_3_(2,6-diCl-PhCO)_2_***: Amounts of 0.07807 g (0.2390 mmol) of SbMe_3_Br_2_ and 0.2000 g (0.4870 mmol) of Tl(2,6-diCl-PhCO) were used in the metathesis reaction. The yield of the dry product was 0.1010 g (71%) based on the starting SbMe_3_Br_2_. The melting point was 184.35 °C. IR spectrum, cm^−1^: 3089, 3077, 2933 ν(C-H) aromatic, aliphatic; 2217 ν(C≡N); 1583 ν(C=N); 1561, 1526 ν(C=C); 1430, 777, 725 δ(C-H); 972 ν(N-O); 632 ν(C-Cl); 561 ν^as^(C-Sb).

***SbMe_3_(TCO)_2_***: Amounts of 0.1956 g (0.5988 mmol) of SbMe_3_Br_2_ and 0.4000 g (1.203 mmol) of Tl(TCO) were used in the metathesis reaction. The yield of the dry product was 0.03100 g (12%) based on SbMe_3_Br_2_. The melting point was from 186.9 to 189.6 °C. ^1^H NMR spectrum in DMSO-d_6_, ppm: 14.34, 10.5 (NH_2_ group in syn-); 9.98, 9.53 (NH_2_ group in anti-). ^13^C{^1^H} NMR spectrum in DMSO-d_6_, ppm: 186.51 (thioamide, anti-), 185.91 (thioamide, syn-), 133.36 (oxime group, syn-), 131.79 (oxime group, anti-), 109.47 (CN group, syn-), 108.83 (CN group, anti-). IR spectrum, cm^−1^: 3440, 3389 r(NH_2_); 2922 ν(C-H); 2236 ν(C≡N); 1693 ν(C=N); 1472, 1331 δ(C-H); 1180, 1068 ν(C=S); 981 ν(N-O); 591 ν^as^(C-Sb).

***SbMe_3_(TDCO)_2_***: Amounts of 0.1168 g (0.3575 mmol) of SbMe_3_Br_2_ and 0.2579 g (0.7153 mmol) of Tl(TDCO) were used in the metathesis reaction. The yield of the dry product was 0.1023 g (41.5%) based on SbMe_3_Br_2_. The melting point was 175.27 °C. ^1^H NMR spectrum in DMSO-d_6_, ppm: 3.45, 3.40 (6H, singlet, CH_3_-groups tertiary amide, accidental overlap), 3.35, 3.17 (3H singlet, CH_3_-groups tertiary amide), 1.79, 1.77, 1.75 (3H, singlet, CH_3_-group Sb). ^13^C{^1^H} NMR spectrum in DMSO-d_6_, ppm: 183.72 (thioamide, anti-), 182.67 (thioamide, syn-), 133.96 (oxime group, syn-), 132.15 (oxime group, anti-), 114.55 (CN group, syn-), 110.09 (CN group, anti-). IR spectrum, cm^−1^: 2932 ν(C-H); 2225 ν(C≡N); 1531 ν(C=N); 1449, 1394 δ(C-H); 1141, 1078, 1050 ν^as^(C=S), 969 ν(N-O); 568 ν^as^(C-Sb).

### 3.2. Microbiology

#### 3.2.1. Bacterial Studies

Media and antimicrobial compounds: Preliminary antimicrobial susceptibility testing was performed using low-salt Luria–Bertani (LB) medium, whereas for determination of minimum inhibitory concentrations (MICs), cation-adjusted Mueller–Hinton broth (MHB) (Sigma-Aldrich, St. Louis, MO, USA) was used as per CLSI standards.

Bacterial strains: Three bacterial pathogens, Shiga toxin-producing *E. coli* strain S17(O113:H4) isolated from the liver of a chick with septicemia [52], respiratory methicillin-resistant *S. aureus* strain NRS70 [53] and lab-adapted burn wound *P. aeruginosa* strain PAO1 [54], were used in this study. For long-term storage, strains were frozen in 10% skim milk at −80 °C. For each experiment, bacteria were streak-inoculated on LB agar plates and grown overnight at 37 °C, from which isolated colonies were picked for subsequent inoculation.

Determination of minimum inhibitory concentration (MIC): Antimony compounds were prepared, and MICs were determined as described in [32]. Briefly, initially solubilized in sterile DMSO at a concentration of 5 mg/mL, the compounds’ working stocks were prepared in MHB at a concentration of 400 µg/mL, then serially diluted to achieve the range of 0.39–200 µg/mL in MHB in a clear-bottom 96-well plate. The final concentration of DMSO in the 200 µg/mL sample was 4%, which is not toxic to bacterial cells. To achieve a CLSI-recommended inoculum size between 2 and 8 × 10^5^ CFU/mL, the bacterial cultures (100 µL), normalized to an OD_600nm_ of 0.02 in MHB [55], were inoculated into the 96-well plate containing 100 µL of prepared dilutions of antimony compounds and incubated at 37 °C for 24 h shaking at 200 rpm. As a control, we used treatment with 10 µg/mL gentamicin (Gm). Bacterial growth was monitored by measuring OD_600nm_ using a Biotek Synergy Mx plate reader (Agilent Technologies, Santa Clara, CA, USA). The concentration of the compound at which growth was not detected was reported as the MIC. All MIC assays were conducted in triplicate for each compound.

Preparation of cells for Transmission Electron Microscopy (TEM): Following a previously reported procedure [32], bacterial cultures grown in MHB for 5 h were split into two tubes for treated and control samples. SbPh_4_ACO was added to the treated sample at 1xMIC, and the control sample received an equivalent volume of the solvent DMSO. Following 1 h of incubation, the cells were harvested, fixed with 2% glutaraldehyde in 0.1 M cacodylate buffer for 2 h at room temperature, washed from the fixative by using cacodylate wash buffer (0.18 M sucrose in 0.06 M cacodylate buffer) 3 times, and postfixed with 1% osmium tetroxide solution for 1 h. Upon subsequent dehydration with 50%, 95% and 100% ethanol treatments, cells were washed three times in propylene oxide and embedded in Embed 812 (Electron Microscopy Sciences, Hatfield, PA, USA). Ultra-thin sections were prepared using a Leica EM UC6 ultramicrotome (Leica, Wetzlar, Germany) equipped with a DiATOME (Quakertown, PA, USA) diamond knife and contrasted with 2.5% aqueous uranyl acetate and 3% lead citrate Reynold’s stain. The sections were examined using a JEOL JEM-2100 Transmission Electron Microscope (JEOL, Tokyo, Japan) at the OSU Microscopy Facility.

#### 3.2.2. Fungal Studies

Media and antimicrobial compounds: Yeast peptone dextrose (YPD, Difco, BD, Franklin Lakes, NJ, USA) medium was used to culture cryptococcal organisms, and RPMI-MOPS, pH 7.0 (RPMI, ThermoFisher, MOPS Sigma-Aldrich), was used for susceptibility testing as per CLSI standards [56].

Fungal strains: The clinical isolates *Cryptococcus neoformans* strain H99 isolated from a patient with cryptococcal meningitis (a kind gift of Dr. John Perfect, Duke University Medical Center) and *Candida albicans* strain SC5314 isolated from a patient with a generalized *Candida* infection (ATCC) were used in this study. The strains were recovered from glycerol stocks frozen at −80 °C. Before each experiment, fungal cells were grown on YPD plates at 30 °C for two days. Isolated colonies were used to inoculate YPD broth, and cultures were grown for 18 h in a shaking incubator at 30 °C. Following the incubation, fungal cells were collected, washed 3 times with sterile phosphate-buffered saline (PBS) and counted using a hemacytometer with trypan blue exclusion dye. The cell number was adjusted to the number needed for each experiment.

Minimum inhibitory concentration (MIC): Antimony compounds were dissolved in sterile, cell culture grade DMSO at a concentration of 5 mg/mL, and diluted to experimental concentrations immediately before use in RPMI-MOPS to 200 μg/mL. The highest concentration of DMSO in this assay was 2% (the 100 µg/mL concentration), which is not toxic to fungal cells. A serial dilution was made in RPMI-MOPS in the range from 1.56 μg/mL to 100 μg/mL in a 96-well plate. The total volume of compounds added was 100 µL/well. Fungal cells at a concentration of 0.5 × 10^3^/mL in a volume of 100 µL/well were added to each concentration of antimony compounds. Fungal cells grown in the absence of antimony compounds served as positive controls for fungal growth, and the negative control consisted of media alone. The positive control for antifungal activity was the antifungal drug Amphotericin B. Plates were incubated at 35 °C in a humidified incubator. Following a 48 h incubation, plates were first visually inspected for fungal growth (turbidity), and then all wells were resuspended, and plates were read at OD_450nm_ using a Biotek Synergy plate reader (Agilent Technologies, Santa Clara, CA, USA). The MIC was the concentration of the compound at which no fungal growth was detected. All MIC assays were conducted in triplicate for each compound.

Minimum fungicidal concentration (MFC): Following MIC measurements (described above), 10 μL from the MIC and any increased concentrations available (from any “clear” wells) were plated on YPD plates and incubated at 30 °C for 48–72 h. Following incubation, the lowest concentration with zero fungal colonies was considered the MFC. All MFC assays were conducted in triplicate for each compound.

## 4. Conclusions

1. A total of 14 compounds were synthesized and characterized: initial trimethylantimony dibromide, 4 thallium(I) arylcyanoximates—precursors for organoantimonials—and 9 methyl–antimony (V) cyanoximates. The latter form the ***G2*** family of trimethylantimony(V) cyanoximates of SnMe_3_L_2_ composition.
2. All synthesized trimethylantimony(V) cyanoximates and their precursors were characterized using crystallography, IR, NMR and TG/DSC analysis. Crystal structures were determined for 13 synthesized compounds. Among them, the structures of eight of the trimethylantimony(V) cyanoximates represent the first examples ever determined in this field.
3. All SnMe_3_L_2_ composition cyanoximates adopt the distorted trigonal bipyramid [SbC_3_O_2_] shape of the coordination polyhedron of antimony(V), with the O-Sb-O angle being less than 180°.
4. Eight synthesized trimethylantimony(V) cyanoximates and their corresponding starting organic ligands were evaluated for their antimicrobial and antifungal activities, including the initial trimethylantimony dibromide used as a control.
5. Two compounds, SbMe_3_(MCO)_2_ and SbMe_3_(2,4-diClPhCO)_2_, were active against bacterial pathogens and inhibited the growth of *P. aeruginosa* and MRSA, with MIC values of 50 and 100 µg/mL, respectively.
6. Three compounds, SbMe_3_(MCO)_2_, SbMe_3_(ECO)_2_ and SbMe_3_(TCO)_2_, were active against two fungal pathogens and inhibited either one of or both *C. albicans* and *C. neoformans* strains at concentrations of 2.6–66.67 μg/mL. In addition, SbMe_3_(TCO)_2_ and SbMe_3_(MCO)_2_ were fungicidal against either *C. neoformans* or both fungi at concentrations of 33.33–66.67 μg/mL.
7. Four of the studied ***G2*** series compounds possess antimicrobial activity with a particular potential against two fungal pathogens, which will be explored in further studies that, among other aspects, will include evaluation of their toxicity against mammalian cells.

## 5. Patent

Patent information (pending): USA Full Patent Application Appl. No. PCT/US22/17565, filed on February 23, 2022, in Oklahoma State University. Title: Organoantimony(V) Cyanoximate Compounds And Methods Of Production And Use Thereof; Inventors: Nikolay Gerasimchuk, Kevin Pinks, Marianna Patrauchan and Karen Wozniak.

## Figures and Tables

**Table 1:** Crystal and refinement data for the trimethylantimony(V) cyanoximates, SbPh_3_L_2_.

Parameter / L	ACO	MCO	ECO	4Cl-PhCO	2,4-DiCl-PhCO	2,6-diCl-PhCO	TCO	TDCO
Empirical formula	C_9_HN_6_O_4_Sb	C_17_H_25_N_6_O_6_Sb	C_13_H_19_N_4_O_6_Sb	C_19_H_17_Cl_2_N_4_O_2_Sb	C_19_H_15_Cl_4_N_4_O_2_Sb	C_19_H_15_Cl_4_N_4_O_2_Sb	C_9_H_13_N_6_O_2_S_2_Sb	C_13_H_21_N_6_O_2_S_2_Sb
Formula weight, g/M	378.906	531.18	449.079	526.02	594.9	594.9	423.12	479.23
Temperature, K	120(2)	120(2)	296(2)	120(2)	296(2)	150(2)	296(2)	296(2)
Crystal system	triclinic	orthorhombic	monoclinic	monoclinic	monoclinic	triclinic	orthorhombic	monoclinic
Color/habitus	colorless needle	colorless plate	colorless block	colorless plate	colorless prism	colorless block	yellow block	yellow plate
Space group	P-1, #2	Pbca, #60	P2_1_/n	P2_1_/n, #14	P2_1_/c, #14	P-1, #2	Pnma, #63	P2_1_/c, #14
Cell constants, Å/°								
a	8.727(2)	9.6396(2)	9.1401(5)	11.683(3)	7.1788(12)	7.540(3)	14.6202(15)	13.4229(13)
b	8.829(2)	12.602(2)	16.0912(9)	7.2655(18)	24.154(4)	10.889(4)	16.0815(16)	12.1231(11)
c	10.349(2)	31.362(5)	13.4416(8)	25.126(6)	13.939(2)	14.709(6)	7.1203(7)	12.9825(12)
α	92.780(4)	90	90	90	90	104.891(6)	90	90
β	92.045(4)	90	103.187(1)	96.141(4)	102.843(3)	93.234(6)	90	103.248(2)
γ	107.665(4)	90	90	90	90	100.956(6)	90	90
Volume (Å^3^)	757.8(3)	4501.6(13)	1924.80(19)	2120.5(9)	2356.5(7)	1138.8(8)	1674.1(3)	2056.4(3)
Z	2	8	4	4	4	2	4	4
ρ_calc_ (g/cm^3^)	1.66	1.568	1.55	1.648	1.677	1.735	1.679	1.548
μ (mm^−1^)	1.84	1.27	1.466	1.575	1.647	1.704	1.906	1.562
F(000)	359	2144	894	1040	1168	584	832	960
Θ range for data (°)	3.94 to 52.86	1.30 to 26.43	4.02 to 50	2.01 to 26.00	1.72 to 25.00	1.98 to 26.37	2.53 to 25.00	1.56 to 26.42
Index ranges:	−10 ≤ h ≤ 10	−14 < h < 14	−12 ≤ h ≤ 12	−13 ≤ h ≤ 14	−8 ≤ h ≤ 8	−9 ≤h ≤ 9	−17 ≤ h ≤ 17	−16 < h < 16
	−11 < k < 11	−15 < k < 15	−22 ≤ k ≤ 22	−8 ≤ k ≤ 8	−28 ≤ k ≤ 28	−13 ≤ k ≤ 13	−19 ≤ k ≤ 19	−15 < k < 14
	−12 < l < 12	−39 < l < 39	−18 ≤ l ≤ 1	−30 ≤ l ≤ 30	−16 ≤ l ≤ 15	−18 ≤ l ≤ 18	−8 ≤ l ≤ 8	−16 < l < 16
Reflections collected	7575	52122	29353	17852	4135	12471	17301	18608
Independent	3096	4621	3375	4240	4135	4636	1532	4878
	[R_int_ = 0.0585]	[R_int_ = 0.1418]	[R_int_ = 0.0263]	[R_int_ = 0.0497]	[R_int_ = 0.1294]	[R_int_ = 0.0707]	[R_int_ = 0.0519]	[R_int_ = 0.0590]
Data/restraints/parameters	3096/0/228	4621/0/360	3375/0/222	4135/0/256	4135/0/274	4636/0/274	1532 0/100	4221/0/231
GOF on *F*^2^	0.989	1.045	1.076	1.036	1.101	0.968	1.141	1.031
Final R, I > 2σ(I)	R1 = 0.0446	R1 = 0.0418	R1 = 0.0253	R1 = 0.0300	R1 = 0.0857	R1 = 0.0541	R1 = 0.0645	R1 = 0.0514
	wR2 = 0.0790	wR2 = 0.0930	wR2 = 0.0654	wR2 = 0.064	wR2 = 0.1417	wR2 = 0.1233	wR2 = 0.1489	wR2 = 0.1011
Peak/hole, e(A^−3^)	1.67 and −1.36	0.59 and −1.30	0.59 and −0.31	0.53 and −0.45	0.85 and −0.88	1.52 and −1.67	4.52 and −1.07	1.79 and −1.25
Volume, A^3^ (%)	458.8 (60.41)	1200.3 (64.0)	1119.1 (58.1)	1313.7 (61.9)	1412.8 (59.9)	721.5 (63.4)	1020.5 (60.1)	1259.2 (61.2)
CCDC #	2376862	2376859	2376857	2376858	2376861	2376863		

**Table 2:** Bacterial minimum inhibitory concentrations (MICs) of Sb-based antimicrobials. The reported numbers represent the average of three experiments; the standard error was calculated and equaled 0. Each antimicrobial was subjected to standard CLSI broth dilution assays at concentrations of 0–200 µg/mL. In this table, >200 indicates that the MIC for the given compound was not detectable at the range of concentrations tested. Compounds that show antibacterial activity compared to their ligand control are highlighted in bold.

Compound	*E. coli*	*P. aeruginosa*	*S. aureus*
Ligand controls, MIC µg/mL			
H(ECO)	>200	>200	>200
H(MCO)	>200	>200	>200
H(2,4-diClPhCO)	200 ± 0.0	>200	150 ± 0.0
H(2-ClPhCO)	>200	>200	>200
Sb-based antimicrobial compounds, MIC µg/mL			
SbMe_3_(ECO)_2_	>200	>200	>200
SbMe_3_(MCO)_2_	>200	**50 ± 0.0**	>200
SbMe_3_(TCO)_2_	>200	>200	>200
SbMe_3_(TDCO)_2_	>200	>200	>200
SbMe_3_(2,4-diClPhCO)_2_	>200	>200	**100 ± 0.0**
SbMe_3_(2,6-diClPhCO) _2_	>200	>200	>200
SbMe_3_(2-ClPhCO) _2_	>200	>200	>200
Antibiotic controls, MIC µg/mL			
Polymyxin B		2 ± 0.0	
Meropenem	1.7 ± 0.3		
Cefoxitin			10.7 ± 4.6

**Table 3:** Fungal minimum inhibitory concentrations (MICs) and minimum fungicidal concentrations (MFCs) of Sb-based antimicrobials. The reported numbers represent the average of three experiments ± standard error of the mean (SEM). Each antimicrobial was subjected to standard CLSI dilution assays at concentrations of 0–100 µg/mL. In this table, >100 indicates that the MIC for the given compound was not detectable at the range of concentrations tested. Compounds that show antifungal activity compared to their ligand control are highlighted in bold.

Compound	*C. albicans*		*C. neoformans*	
	MIC (μg/mL)	MFC (μg/mL)	MIC (μg/mL)	MFC (μg/mL)
Ligand controls				
H(ECO)	>100	>100	>100	>100
H(MCO)	>100	>100	>100	>100
H(2,4-diClPhCO)	100 ± 0.0	100	25 ± 0.0	100 ± 0.0
H(2-ClPhCO)	100 ± 0.0	>100	25 ± 0.0	>100
Sb-based antimicrobial compounds				
SbMe_3_(ECO)_2_	100 ± 0.0	>100	**20.8 ± 7.2**	>100
SbMe_3_(MCO)_2_	**25 ± 0.0**	**66.7 ± 28.9**	**2.6 ±0.9**	**33.3 ± 14.4**
SbMe_3_(TCO)_2_	**50 ± 0.0**	>100	**12.5 ± 0.0**	**50 ± 0.0**
SbMe_3_(TDCO)_2_	>100	>100	>100	>100
SbMe_3_(2,4-diClPhCO)_2_	>100	>100	>100	>100
SbMe_3_(2,6-diClPhCO)_2_	>100	>100	100 ± 0.0	>100
SbMe_3_(2-ClPhCO)_2_	>100	>100	>100	>100

## Data Availability

Data is contained within the article and Supplementary Materials. Results of crystallographic characterization of presented in the text new organoantimonials as well as several key precursors were deposited into the Cambridge Crystallographic Data Center. Respective CCDC numbers are shown in Table 1.

## Supplementary Materials

The following supporting information can be downloaded at https://www.mdpi.com/article/10.3390/molecules29235779/s1: Pages of Electronic Supplementary Materials are available. These are as follows: antimicrobial compounds and uses of antimony, *ESI 1*; *ESI 3*; crystal structures of important Tl(I) salts for some compounds, *ESI 2*–*12*; obtaining of key precursors, Tl(I) salts, *ESI 13*; tables of selected bond lengths and valence angles in structures of organoantimony(V) cyanoximates, *ESI 14*–*21;* thermal analysis traces for SbMe_3_(ECO)_2_, *ESI 22*; thermal analysis traces for SbMe_3_(2,6-diCl-PhCO)_2_, *ESI 23*; data of elemental analyses for all prepared compounds, *ESI 24*; crystal structure of the antimony-source Sb(CH_3_)_3_Br_2_, *ESI 25*–*27*; videomicroscope images of indexed single crystals of some organoantimony(V) cyanoximates, *ESI 28*; multiple pages of PLATON checkCIF reports for all organoantimony(V) compounds, *ESI 29*; PLATON checkCIF reports for three key precursors of Tl(I) cyanoximates, *ESI 30*; synthesis of organoantimonials, *ESI 31* and *32*.
