# Supplementary material for: Synthesis, Characterization and Antimicrobial Activity of Trimethylantimony(V) Biscyanoximates, a New Family of Antimicrobials
Source: Molecules. 2024 Dec 6;29(23):5779. doi: 10.3390/molecules29235779 (PMC11643687; doi:10.3390/molecules29235779)
Supplement: Supplementary file 1 [file molecules-29-05779-s001.zip › molecules-3215129-supplementary.pdf]

PAGES OF ELECTRONIC SUPPORTING INFORMATION SECTION FOR THE PAPER:

Background information about antimicrobial inorganic compounds and uses of antimony.

Although most of our exposure to antimicrobials is attributed to the 1900s with the emergence of the modern era of antibiotics, traces of tetracycline – an antibiotic – found in the human skeletal remains from ancient Sudanese Nubia dating back to 305-550 CE suggest that some were already in use. Paul Ehrlich is known widely as the father of antimicrobial chemotherapy and bioinorganic chemistry because with the help of his Japanese assistant Sahachiro Hata, they were able to find the “magic bullet” (compound 606) capable of treating infectious microbes without also killing the host. This compound is an arsenic-based chemical ($C_{12}H_{13}As_2ClN_2O_2$), was used to successfully treat syphilis and was later marketed as Salvarsan, meaning “Lifesaving”. Ehrlich designed methods in search of this effective compound, and such strategy is currently still being used.

One of the most interesting elements from pnictogens family is antimony because its compounds showed great potential in the recent past.

With multi-drug resistant (MDR) microorganisms, synthetic antimicrobials may be the way out as specific drugs can be synthesized to affect specific drug target sites. Element-organic compounds like organo-arsenic and organotin have a great history in the search for antibiotics to treat various diseases. While there is a plethora of information on arsenic, other inorganic compounds may offer antimicrobial properties that have not yet been explored. Henceforth, they have been used in colloidal solutions for topical applications, coatings for various implants and as fabric for wound treatment. With addition of a variety of organometallic complexes to research, the possibility of finding novel antimicrobials is practically endless.

Antimony’s use can be dated back to the early Egyptians. It has been used as coatings for other metals, or for cosmetic purposes – even as eyeliner. Other uses of antimony are in flame retardants, in semi-conductor devices, and in numerous alloys employed in batteries and making ammunition. Medical uses include a compound with tartaric acid and Sb (III), was used as a treatment drug for leishmaniasis, but had numerous side effects such as coughs and depression and even death due to its high toxicity. One of the successful uses of antimony is in the treatment of Leishmaniasis. Leishmaniasis is caused by a protozoan parasite of genus Leishmania, transmitted through the bite of infected sandflies. This results in either cutaneous leishmaniasis (**CL**) or visceral leishmaniasis (**VL**), with the latter being most severe and leading to death. The spread of Leishmaniasis in South and Central America, Bangladesh, southern Europe, and North Africa led to the reliance on antimony-based compounds for drug therapy. In the modern era, the biological aspect of antimony has been explored and applied. Thus, antimony compounds have been used to treat diseases such as trypanosomiasis and leishmaniasis because they exhibit appreciable effects as biocides, fungicides, antitumor agents and antioxidants. Pentavalent antimonials (V) such as sodium antimony gluconate (*Pentostam*) and meglumine antimonate (*Glucantime*) have been in use for more than six decades to treat Leishmaniasis and Indian kala-azar disease. Additionally, research in this area established that organoantimony compounds show antimicrobial, antifungal, and antitumor activity.

Crystal structures of important precursors – auxiliary complexes of Tl(I) and thioamide-cyanoximes.

Crystal structure of Tl(4-Cl-PhCO).

The crystal and refinement data are shown in Table 2. The asymmetric unit (ASU) structure of Tl(4-Cl-PhCO) is shown in Figure 11. All the hydrogens within the complex were identified on the electron difference map and refined to create this ASU structure. The cyanoxime anion adopts an anti-geometry with no trans or cis assignment of geometry since the chlorine atom is in the para position to the cyanoxime fragment. Selected bond lengths and valence angles within the molecule are presented in Table 7. The two fragments present within the molecule are the chloro-aryl group and the cyanoxime group. Both fragments are planar, but the molecule is non-planar. This is due to the dihedral angle between the chloro-aryl group and the cyanoxime group which is 9.47°. This angle was calculated using mean planes Cl1-C6-C7-C8-C3-C4-C5 and N2-C3-C1-N1-O1, respectively. The bond lengths for N1-C1 = 1.308(7) Å and N1-O1 = 1.340(6) Å are within range of bond lengths observed for the cyanoxime group in similar classes of compounds and evidence this time an *oxime* character of the group. The π-π stacking interactions between 4-chlorophenyl rings of neighboring molecules holds the structure together in packing with the distance between the two closest centroids of the 4-chlorophenyl rings being 4.085 Å.

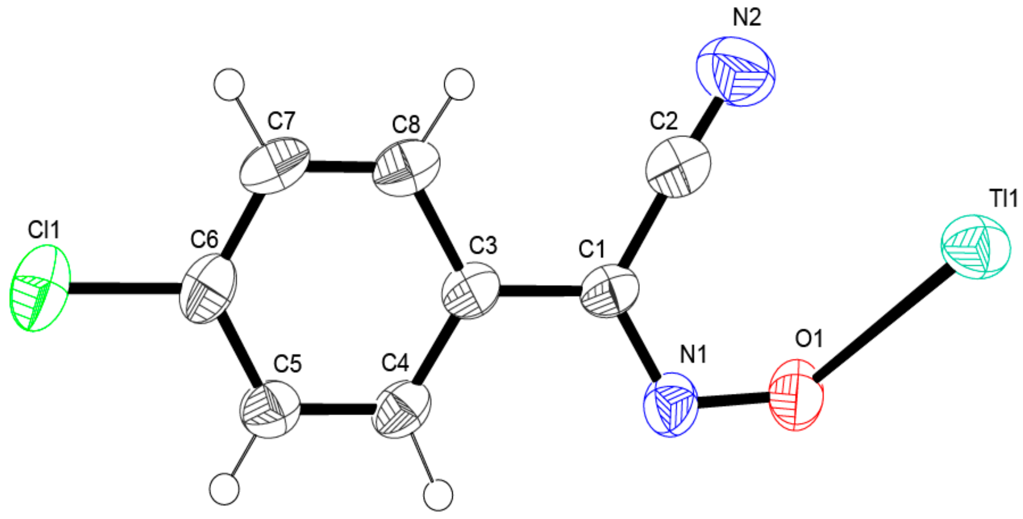

Figure S1. The ASU and atomic numbering scheme in the structure of Tl(4-Cl-PhCO) drawn in ORTEP representation at 50% ellipsoid probability level.

Table S1. Crystal and refinement data for Tl (4-Cl-PhCO).

Empirical formula	$C_8H_4ClN_2OTl$	
Formula weight, g/mol	383.95	
Temperature, K	296(2)	
Wavelength, Å	0.71073 (Mo Kα)	
Color	yellow	
Crystal system	monoclinic	
Space group	P 2_1/n	
Unit cell dimensions, Å, °	a = 10.951(4)	α = 90
	b = 4.0853(15)	β = 99.041(4)
	c = 20.616(7)	γ = 90
Unit cell volume, $Å^3$	910.9(6)	
Z	4	
Density (calculated), g/cm^3	2.800	
Absorption coefficient, mm^{-1}	17.980	
F (000)	688	
Θ range, °	2.00 to 28.90	
Index ranges	-14 ≤ h ≤ 14	
	-5 ≤ k ≤ 5	
	-27 ≤ l ≤ 28	
Reflections collected	10544	
Independent reflections	2391 [R_{int} = 0.0544]	
Data / restraints / parameters	2391 / 0 / 130	
Goodness-of-fit on F^2	1.025	
Final R indices [I>2σ(I)], 1893 data	R1 = 0.0362	
	wR2 = 0.0829	
R indices [all data]	R1 = 0.0490	
	wR2 = 0.0892	
Largest diff. peak and hole, e $Å^{-3}$	1.857 and -2.041	
CCDC registration number:	2380468	

Table S2. Selected bond lengths and valence angles of the ligand in the structure of Tl(4-Cl-PhCO).

Bond length, Å		Valence angle,°	
O1 - N1	1.340(6)	C1 - N1 - O1	115.9(4)
N1 - C1	1.308(7)	C2 - C1 - N1	119.4(5)
C1 - C2	1.440(8)	N1 - C1 - C3	120.1(5)
C1 - C3	1.474(8)	C3 - C1 - C2	120.5(5)
C2 - N2	1.133(8)	N2 - C2 - C1	177.6(7)
Cl1 - C6	1.734(6)	N1 – O1 - TlO1	90.3(3)

Crystal structure of Tl(2,4-diCl-PhCO).

The crystal specimen was an inversion twin, and it was resolved using specialized CELL_NOW–t and then TWINABS programs pointing out on a centric monoclinic $C_{2/c}$ space group. The crystal and refinement data for Tl(2,4-diCl-PhCO) is presented in Table S4. The ASU is shown below. All 3 hydrogens on the phenyl ring were attached to their hosting C-atoms and refined isotropically. Selected bond lengths and valence angles in this structure can be seen in Table S5. This compound forms an elegant centrosymmetric dimer (Figure 13A) with both cyanoxime anions on each side adopting a *trans*-anti geometry. Contrary to other studied in our laboratory Tl-derivatives, this compound has a highly non-planar structure comprised of several planar fragments. The dihedral angle, α, formed between mean planes of the cyanoxime group and the chloro-aryl group was 51.49° (Figure 13B). In comparison with the initial ligand H(2,4-diCl-PhCO), the dihedral angle is almost the same having 50.61° value. The bonds in the cyanoxime group were N1-O1 = 1.294(12) Å and N1-C1 = 1.314(13) Å indicating *nitroso* character of the anion. There is slipped and distant π-π stacking interactions between the 2,4-chlorophenyl groups at 3.869 Å contribute to the lattice stabilization and the overall architecture of the structure. This complex has a columnar 1D structure comprised of centrosymmetric dimers.

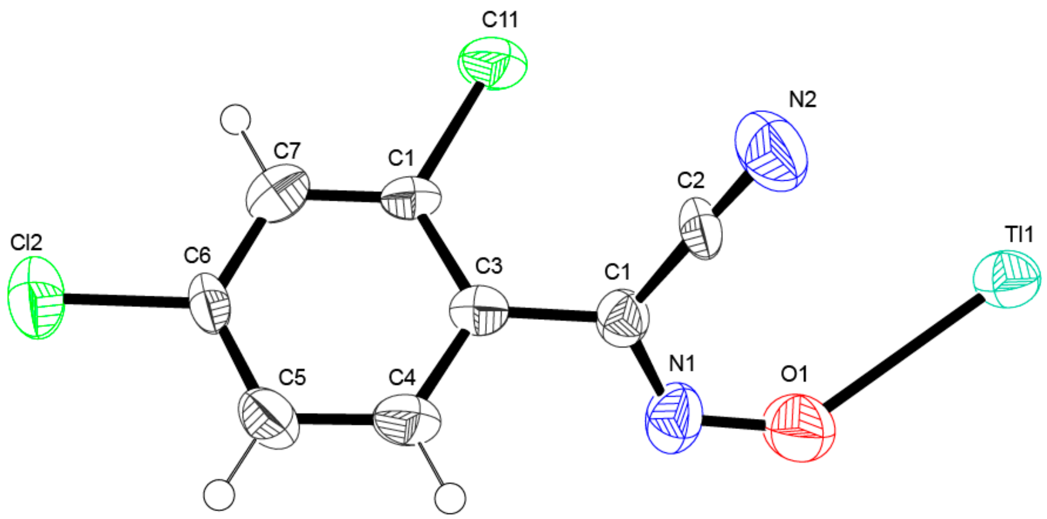

Figure S2. The ASU and atomic numbering scheme in the structure of Tl(2,4-diCl-PhCO) drawn in ORTEP representation.

Table S3. Crystal and refinement data for Tl(2,4-diCl-PhCO).

Empirical formula	$C_8H_3Cl_2N_2OTl$	
Formula weight, g/mol	418.41	
Temperature, K	296.15	
Wavelength, Å	1.54184 (Cu Kα)	
Color	Light-yellow	
Crystal system	Monoclinic	
Space group	C 2/c	
Unit cell dimensions, Å,°	a = 37.013(9)	α = 90
	b = 3.8692(10)	β = 107.072(3)
	c = 14.624(4)	γ = 90
Unit cell volume, Å^3	2002.0(9)	
Z	8	
Density (calculated), g/cm^3	2.7761	
Absorption coefficient, mm^{-1}	16.638	
F (000)	1487.0	
Θ range,°	4.6 to 66.02	
Index ranges	$-54 \le h \le 50$,	

	$-5 \le k \le 5$,
	$-21 \le l \le 21$
Reflections collected	10667
Independent reflections	3433 [R_{int} = 0.0437]
Data / restraints / parameters	3433 / 0 / 122
Goodness-of-fit on F^2	1.037
Final R indices [I>2σ(I)]	R1 = 0.0564
	wR2 = 0.1164
R indices [all data]	R1 = 0.0822
	wR2 = 0.1282
Largest diff. peak and hole, $eÅ^{-3}$	2.81 and -3.29
CCDC registration number:	2380467

SI 7

Table S4. Selected bond lengths and valence angles of the ligand Tl(2,4-diCl-PhCO).

Bond length, Å		Valence angle,°	
O1 - N1	1.294(12)	C1 - N1 - O1	115.8(10)
N1 - C1	1.314(13)	C2 - C1 - N1	121.3(9)
C1 - C2	1.402(13)	N1 - C1 - C3	117.5(9)
C1 - C3	1.466(13)	C3 - C1 - C2	120.4(9)
C2 - N2	1.151(13)	N2 - C2 - C1	177(1)
Cl1 – C8	1.734(9)		
Cl2 – C6	1.756(9)		

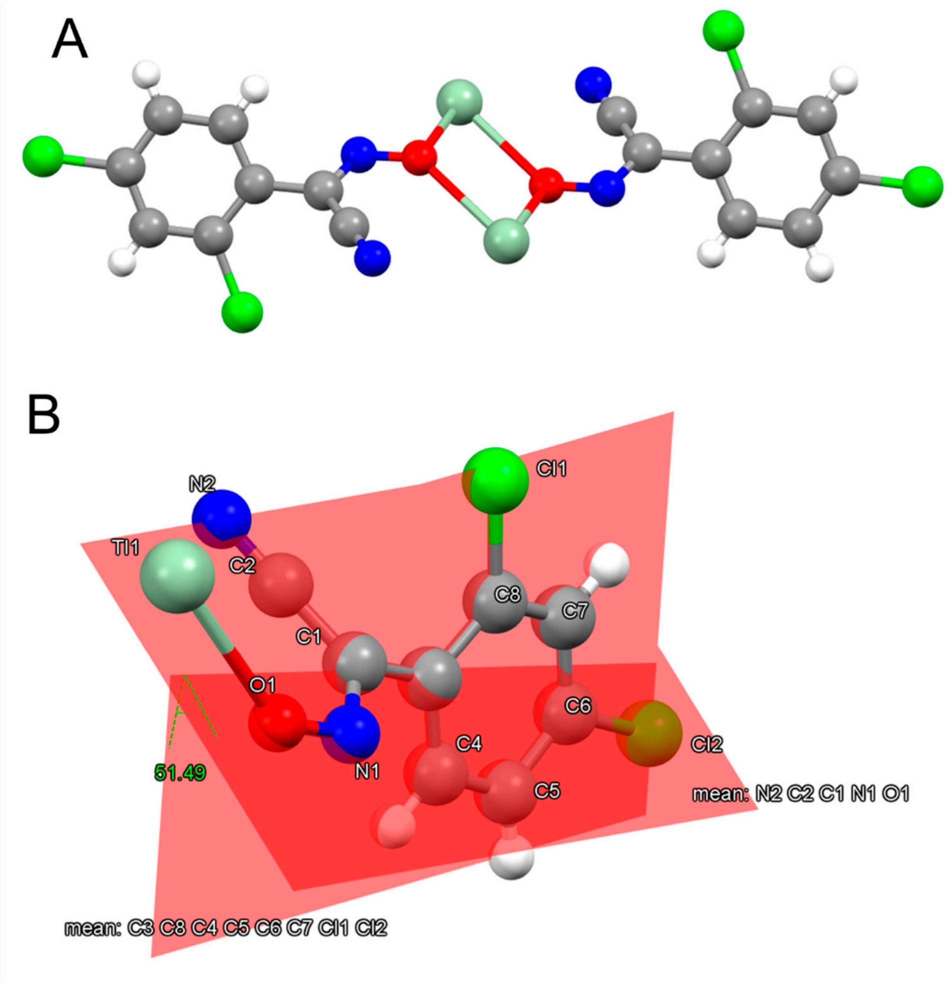

Figure S3. Molecular structure of Tl(2,4-diCl-PhCO): **A** - Dimeric GROW structure of the Tl(2,4-diCl-PhCO); **B** – non-planar core of the structure expressed is evident as a large dihedral angle between the cyanoxime and the chloro-aryl group.

Crystal structure of Tl(2,6-diCl-PhCO).

Its crystalline specimen proved difficult to work out because it turned out to be multidomain material. Out of five domains of reflections only the main, largest domain was used for the structure solution and subsequent refinement. All three H-atoms were geometrically attached to their hosting C-atoms at sp^2 hybridization. The crystal data for Tl(2,6-diCl-PhCO) is shown herein in Table S6. The asymmetric unit for Tl(2,6-diCl-PhCO) is shown in Figure S3, while important bond lengths and valence angles are shown in Table S7. The Tl(2,6-diCl-PhCO) also forms a centrosymmetric dimer (Figure S3B). The anion is heavily non-planar, adopts anti-geometry and represents an *oxime*. The dihedral angle between the dichloroaryl group and the cyanoxime (Figure S3A) was 57.62°. The bond lengths for N1-C1 = 1.30(3) Å and N1-O1 = 1.35(2) Å are within the normal ranges for the cyanoxime bond lengths in similar class of compounds. Electrostatic and van-der-Waals interactions stabilize the crystal structure. Especially important are π–π stacking interactions between neighboring dichloroaryl fragments.

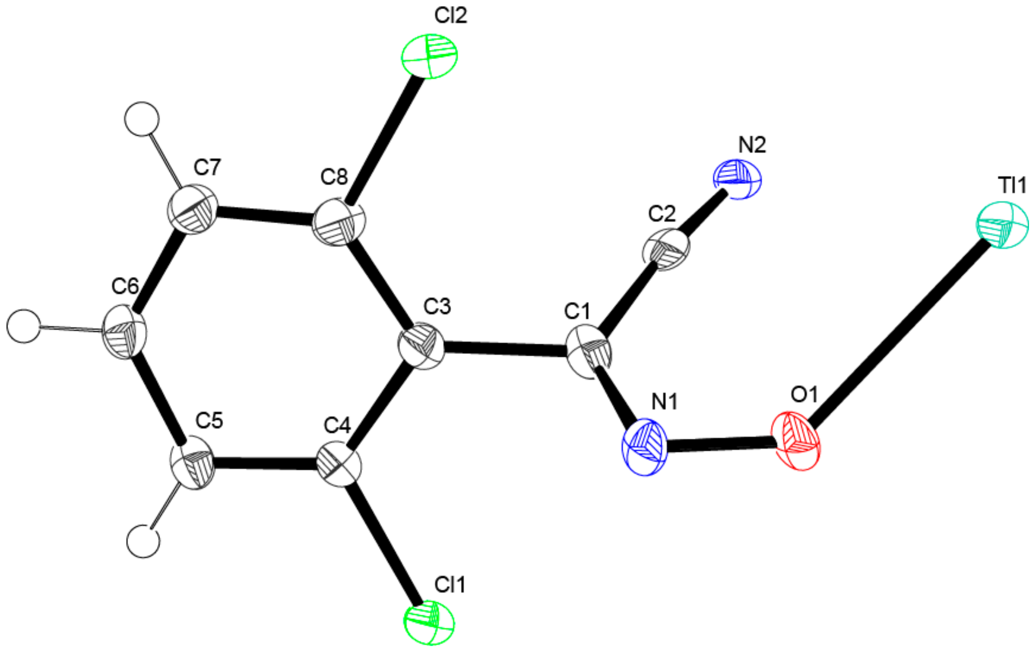

Figure S4. The ASU in the structure of one of the initial compounds Tl(2,6-diCl-PhCO) drawn in ORTEP representation.

Table S5. Crystal and refinement data for Tl(2, 6-diCl-PhCO).

Empirical formula	$C_8H_4Cl_2N_2OTl$	
Formula weight, g/mol	419.40	
Temperature, K	150(2)	
Wavelength, Å	0.71073 (Mo Kα)	
Color	clear light yellow	
Crystal system	monoclinic	
Space group	$P2_1/n$	
Unit cell dimensions, Å, °	a = 4.070(12)	α = 90
	b = 7.73(2)	β = 91.01(5)
	c = 31.04(9)	γ = 90
Unit cell volume, $Å^3$	976.(5)	
Z	4	
Density (calculated), g/cm^3	2.852	
Absorption coefficient, mm^{-1}	17.047	
F (000)	756	
Θ range, °	2.63 to 30.52	
Index ranges	-1 ≤ h ≤ 5	
	-10 ≤ k ≤ 10	
	-31 ≤ l ≤ 33	
Reflections collected	3816	
Independent reflections	2440 [R_{int} = 0.0966]	
Data / restraints / parameters	2440 / 110 / 122	
Goodness-of-fit on F^2	0.921	
Final R indices [I>2σ(I)], 1275 data	R1 = 0.0796	
	wR2 = 0.1676	
R indices [all data]	R1 = 0.1539	
	wR2 = 0.2004	
Largest diff. peak and hole, $eÅ^{-3}$	4.768 and -3.392	
CCDC registration number:	2380469	

Table S6. Selected bond lengths and valence angles of the ligand Tl(2,6-diCl-PhCO).

Bond length, Å		Valence angle,°	
O1 - N1	1.35(2)	C1 - N1 - O1	111.2(18)
N1 - C1	1.30(3)	C2 - C1 - N1	125.0(2)
C1 - C2	1.39(3)	N1 - C1 - C3	115.6(19)
C1 - C3	1.54(3)	C3 - C1 - C2	119.0(2)
C2 - N2	1.13(3)	N2 - C2 - C1	173.0(3)
Cl1 – C4	1.77(2)		
Cl2 – C8	1.77(2)		

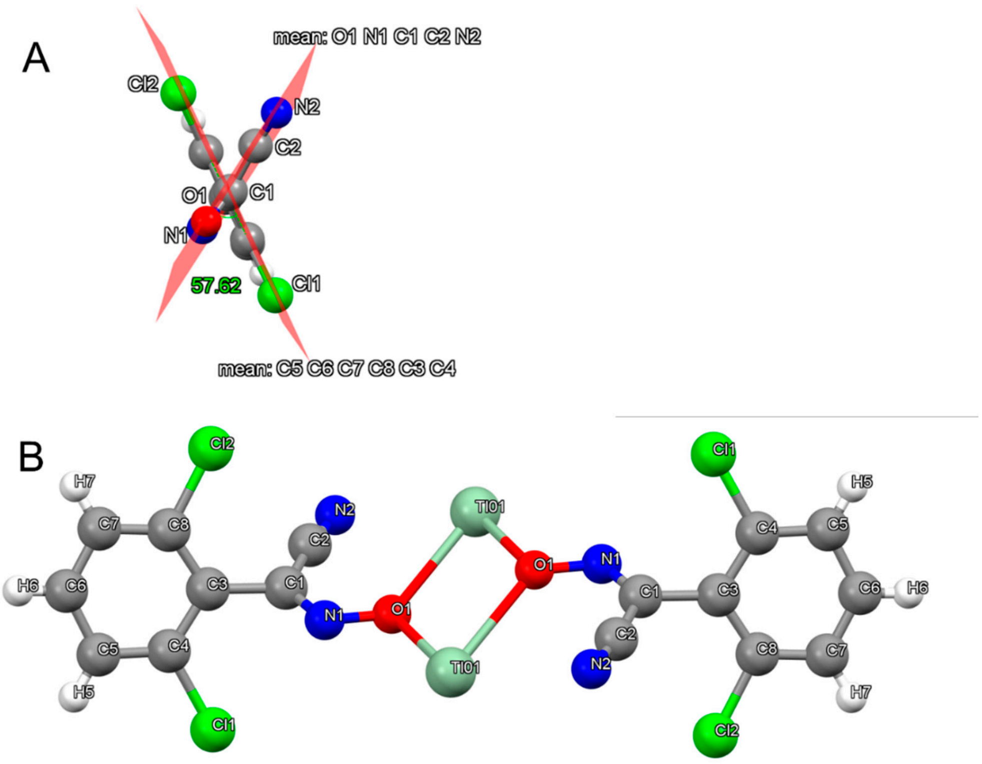

Figure S5. Molecular structure of Tl(2,6-diCl-PhCO) in GROW mode: **A** - Dihedral angle α (between the cyanoxime and the dichloroaryl group); **B** - Dimeric structure of Tl(2,6-diCl-PhCO).

Obtaining key precursors for the synthesis of $SbMe_3L_2$ complexes using Tl-salts of cyanoximes.

A heterogeneous reaction between Tl_2CO_3 in solution and solid HL in aqueous solutions at elevated temperatures leads to quantitative preparation of TlL accompanied with liberation of CO_2 gas:

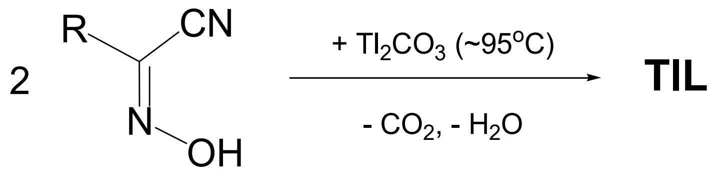

After slow cooling of a yellow solution needle-shaped crystals appeared in abundance in a great yield over 60%. Crystals were filtered, washed with cold water and dried in a desiccator. Typical preparation is shown below for Tl(2Cl-PhCO).

The 0.7158 g (1.5 mmol) of Tl_2CO_3 were dissolved in 20 cm^3 of boiling distilled water. Small portions (0.552 g; 3.05 mmol) of the powdery solid cyanoxime were added to hot (~95°C) solution of thallium carbonate within 5 min leading to a bright yellow solution. After all CO_2 gas had been evolved, the reaction mixture was filtered, and the mother liquor was placed in a large test tube which then was immersed in a Dewar flask containing 5 cm^3 of water at 95°C for slow cooling. The first portion of long needle crystals of TlL, unsuitable for the X-ray analysis, was filtered off and the remaining yellow solution was allowed to evaporate at room temperature over three to four weeks. Good quality single crystals of the complex were obtained. The combined yield of TlL is practically quantitative; m.p. with decomposition ~182°C.

Elemental analysis calculated for $C_8H_4ClN_2OTl$ (%): C, 25.02; H, 1.05; N, 7.30; found: C, 24.84; H, 1.00; N, 7.20.

IR spectrum (cm^{-1}): 3073 ν^{as}(C–H)arom, 3050 ν^{s}(C–H)arom, 2202 ν(C≡N), 1484 ν(C≡C), 1098 ν(N–O), 749 and 726 ν(C–Cl, for orthosubstituted Ph group).

It was the only convenient and high yield method of synthesis of organoantimonials where L = $(2Cl\text{-}PhCO)^-$, $(4Cl\text{-}PhCO)^-$, $(2,4\text{-}diCl\text{-}PhCO)^-$, $(2,6\text{-}diCl\text{-}PhCO)^-$, TCO^- and $TDCO^-$ anions because corresponding Ag(I) salts of these ligands do not exist or highly light-sensitive.

Table S7. Selected bond lengths and valence angles of $SbMe_3(ECO)_2$.

Bond length, Å		Valence angle,°	
Cyanoxime:			
O1 - N1	1.336(3)	C1 - N1 - O1	115.1(2)
N1 - C1	1.294(4)	C2 - C1 - N1	123.7(3)
C1 - C2	1.442(5)	N1 - C1 - C3	119.9(3)
C1 - C3	1.476(4)	C3 - C1 - C2	116.3(3)
C2 - N2	1.127(4)	N2 - C2 - C1	178.6(4)
N3 - O4	1.345(3)	O3 - C3 - O2	124.6(3)
N3 - C6	1.291(4)	C6 - N3 - O4	114.6(3)
		C7 - C6 - N3	122.5(3)
		N3 - C6 - C8	121.5(3)
		C7 - C6 - C8	116.0(3)
		N4 - C7 - C6	178.3(4)
		O5 - C8 - O6	121.7(4)
		O6 - C9 - C10	105.1(6)
Metal Center:			
Sb1 - O1	2.134(2)	C12 - Sb1 - C11	120.9(2)
Sb1 - O4	2.115(2)	C13 - Sb1 - C11	120.3(18)
Sb1 - C11	2.078(3)	C13 - Sb1 - C12	118.8(18)
Sb1 - C12	2.083(3)	O1 - Sb1 - C11	90.80(12)
Sb1 - C13	2.075(4)	O1 - Sb1 - C12	91.56(12)
		O1 - Sb1 - C13	87.10(12)
		O4 - Sb1 - C11	91.82(12)
		O4 - Sb1 - C12	91.65(12)
		O4 - Sb1 - C13	86.99(13)
		O4 - Sb1 - O1	174.1(8)

Table S8. Selected bond lengths and valence angles in the structure of $SbMe_3(4\text{-}Cl\text{-}PhCO)_2$.

Bond length, Å		Valence angle,°	
Cyanoxime:			
O1 - N1	1.354(3)	C1 - N1 - O1	112.9(3)
N1 - C1	1.306(4)	C2 - C1 - N1	120.5(3)
C1 - C2	1.456(5)	N1 - C1 - C3	121.0(3)
C1 - C3	1.472(5)	C3 - C1 - C2	118.5(3)
C2 - N2	1.135(4)	N2 - C2 - C1	176.4(4)
Cl1 - C6	1.741(4)		
Cl2 - C14	1.754(3)		
Metal Center:			
Sb1 - O1	2.093(2)	C17-Sb1-C18	120.29(15)
Sb1 - O2	2.111(2)	C18-Sb1-C19	117.01(17)
Sb1 - C17	2.087(3)	C18-Sb1-O1	95.71(12)
Sb1 - C18	2.088(3)	C17-Sb1-O2	90.43(11)
Sb1 - C19	2.092(4)	C19-Sb1-O2	91.32(12)
		C17-Sb1-C19	122.69(16)
		C17-Sb1-O1	91.06(12)
		C19-Sb1-O1	84.29(12)
		C18-Sb1-O2	87.21(11)
		O1-Sb1-O2	175.49(8)

Table S9. Selected bond lengths and valence angles in the structure of $SbMe_3(2,4\text{-diCl-PhCO})_2$

Bond length, Å		Valence angle,°	
Cyanoxime:			
O1 - N1	1.329(12)	C1 - N1 - O1	113.5(9)
N1 - C1	1.285(14)	C2 - C1 - N1	118.9(10)
C1 - C2	1.464(17)	N1 - C1 - C3	121.3(11)
C1 - C3	1.462(16)	C3 - C1 - C2	119.7(10)
C2 - N2	1.130(15)	N2 - C2 - C1	176.0(14)
N3 - C12	1.388(18)	C12 – N3 – O2	109.8(12)
N3 - O2	1.285(15)	C13 - C12 – N3	112.5(12)
		N3 - C12 – C14	126.4(13)
		C14 - C12 – C13	121.1(11)
		N4 – C13 - C12	174.9(16)
Metal Center:			
Sb1 - O1	2.100(8)	C10 - Sb1 – C9	117.4(5)
Sb1 - O2	2.184(10)	C11 - Sb1 - C9	122.4(5)
Sb1 - C9	2.079(12)	C10 - Sb1 - O1	120.2(5)
Sb1 - C10	2.065(13)	C9 - Sb1 - O1	94.0(4)
Sb1 - C11	2.099(13)	C10 - Sb1 - O1	92.2(4)
		C11 - Sb1 - O1	84.9(5)
		C9 - Sb1 - O2	91.1(4)
		C10 - Sb1 – O2	83.9(4)
		C11 - Sb1 - O2	93.8(5)
		O1 - Sb1 - O2	174.6(3)

Table S10. Selected bond lengths and valence angles in the structure of $SbMe_3(2,6\text{-diCl-PhCO})_2$.

Bond length, Å		Valence angle,°	
Cyanoxime:			
O1 - N1	1.359(7)	C1 - N1 - O1	113.1(5)
N1 - C1	1.282(8)	C2 - C1 - N1	122.2(6)
C1 - C2	1.464(9)	N1 - C1 - C3	121.2(6)
C1 - C3	1.481(9)	C3 - C1 - C2	116.5(6)
C2 - N2	1.134(9)	N2 - C2 - C1	176.3(7)
N3 - O2	1.354(7)	C9 - N3 - O2	112.7(5)
N3 - C9	1.273(8)	C11 - C9 - N3	118.0(6)
		N3 - C9 - C10	123.4(6)
		C11 - C9 - C10	118.6(5)
		N4 - C10 - C9	177.2(8)
Metal Center:			
Sb1 - O1	2.115(4)	C18 - Sb1 - O2	92.7(2)
Sb1 - O2	2.091(4)	O2 - Sb1 - C19	83.6(2)
Sb1 - C17	2.098(7)	O2 - Sb1 - C17	93.7(2)
Sb1 - C18	2.083(6)	C18 - Sb1 - O1	87.3(2)
Sb1 - C19	2.098(7)	C19 - Sb1 - O1	92.5(2)
		C18 - Sb1 - C19	120.2(3)
		C18 - Sb1 - C17	118.5(3)
		C19 - Sb1 - C17	121.3(3)
		O2 - Sb1 - O1	175.55(18)
		C17 - Sb1 - O1	90.2(2)

Table S11. Selected bond lengths and valence angles of $SbMe_3(ACO)_2$.

Bond length, Å		Valence angle,°	
Cyanoxime:			
C1 - C2	1.437(6)	C3 - C1 - C2	118.2(4)
C1 - C3	1.502(6)	O1 - N1 - C1	113.5(4)
C1 - N1	1.292(6)	N1 - C1 - C2	123.4(4)
C2 - N2	1.144(6)	O3 - N4 - C4	114.5(4)
C3 - N3	1.320(6)	N1 - C1 - C3	118.5(4)
C3 -O2	1.220(5)	N2 - C2 - C1	178.8(6)
C4 - C5	1.441(7)	N3 - C3 -C1	115.8(4)
C4 - C6	1.496(6)	O2 - C3 - C1	119.0(4)
C4 - N4	1.291(6)	O2 - C3 - N3	125.2(5)
C5 - N5	1.143(6)	C6 - C4 - C5	117.6(4)
C6 - N6	1.316(6)	N4 - C4 - C5	122.6(4)
C6 - O4	1.229(5)	N4 - C4 - C6	119.8(4)
N1 - O1	1.341(5)	N5 - C5 - C4	178.7(6)
N4 - O3	1.337(5)	O4 - C6 - N6	124.6(5)
Metal Center:			
C7 - Sb1	2.082(7)	Sb1 - O1 - N1	112.7(2)
C8 - Sb1	2.079(6)	Sb1 - O3 - N4	114.8(3)
C9 - Sb1	2.092(7)	O1 - Sb1 - C7	93.1(2)
O1 - Sb1	2.123(3)	O1 - Sb1 - C8	84.2(2)
O3 - Sb1	2.140(3)	O1 - Sb1 - C9	94.9(2)
		O3 - Sb1 - C7	86.0(2)
		O3 - Sb1 - C8	93.0(2)
		O3 - Sb1 - C9	89.1(2)
		O3 - Sb1 - O1	175.95(14)

Table S12. Selected bond lengths and valence angles of $SbMe_3(TCO)_2$.

Bond length, Å		Valence angle,°	
Cyanoxime:			
C1 - N1	1.304(11)	N1-C1-C2	122.0(8)
C1 - C2	1.433(13)	N1-C1-C3	119.2(8)
C1 - C3	1.476(12)	C2-C1-C3	118.7(7)
C2 - N2	1.132(11)	N2-C2-C1	174.9(10)
C3 - N3	1.304(12)	N3-C3-C1	114.3(8)
C3 - S1	1.648(9)	N3-C3-S1	124.7(8)
N1 - O1	1.337(9)	C1-N1-O1	113.5(7)
Metal Center:			
C4 - Sb1	2.093(15)	N1-O1-Sb1	111.3(5)
C5 - Sb1	2.069(14)	C6-Sb1-O1	91.54(19)
C6 - Sb1	2.061(15)	C5-Sb1-O1	93.73(18)
Sb1 - O1	2.125(6)	C4-Sb1-O1	84.93(17)
		C5-Sb1-O1	93.73(18)
		C6-Sb1-O1	91.54(19)
		C4-Sb1-O1	84.93(17)
		O1-Sb1-O1	169.6(3)
		C6-Sb1-C5	116.7(7)
		C6-Sb1-C4	119.5(7)
		C5-Sb1-C4	123.9(7)

Table S13. Selected bond lengths and valence angles in the structure of $SbMe_3(TDCO)_2$.

Bond length, Å		Valence angle,°	
Cyanoxime:			
O1 - N1	1.340(6)	C1 - N1 - O1	111.4(5)
N1 - C1	1.306(7)	C2 - C1 - N1	113.3(5)
C1 - C2	1.452(9)	N1 - C1 - C3	128.3(6)
C1 - C3	1.488(8)	C3 - C1 - C2	117.7(5)
C2 - N2	1.135(8)	N2 - C2 - C1	177.4(7)
S1 – C3	1.663(6)	C1 - C3 - S1	116.4(4)
S2 - C8	1.672(7)	N3 - C3 - C1	118.5(5)
C6 - N4a	1.55(4)	N3 - C3 - S1	125.1(5)
C6 - N4	1.299(9)	C4 - N3 - C5	114.3(6)
N4a - O2a	1.37(5)	C9 - N6 - C10	114.2(6)
N4 - O2	1.352(9)	N5 - C7 - C6	178.7(8)
anti - geometrical configuration:			
		C7 - C6 - N4a	148.7(16)
		C8 - C6 - N4a	93.50(15)
		Sb1 - O2a - N4a	105.0(2)
syn - geometrical conformation:			
		C7 - C6 - N4	117.5(5)
		C8 - C6 - N4	134.2(6)
		Sb1 - O2 - N4	106.8(4)
Metal Center:			
Sb1 - O1	2.114(4)	O1 - Sb1 - O2	174.2(18)
Sb1 - O2	2.163(6)	O1 - Sb1 - O2a	150.1(7)
Sb1 – O2a	2.080(3)	C11 - Sb1 - C12	120.2(3)
Sb1 - C11	2.080(6)	C11 - Sb1 - C13	121.4(3)
Sb1 - C12	2.082(6)	C11 - Sb1 - O2a	114.7(7)
Sb1 - C13	2.072(6)	C11 - Sb1 - O1	92.30(2)
		C11 - Sb1 - O2	83.20(2)
		C12 - Sb1 - C13	118.4(3)
		C12 - Sb1 - O2a	84.90(8)
		C12 - Sb1 - O2	92.60(2)
		C12 - Sb1 - O1	92.70(2)
		C13 - Sb1 - O1	86.10(2)
		C13 - Sb1 - O2	93.20(3)
		C13 - Sb1 - O2a	69.30(8)

Table S14. Selected bond lengths and valence angles of $SbMe_3(MCO)_2$.

Bond length, Å		Valence angle,°	
Cyanoxime:			
O1 - N1	1.350(5)	C1 - N1 - O1	114.2(4)
N1 - C1	1.285(7)	C2 - C1 - N1	120.8(5)
C1 - C2	1.453(7)	N1 - C1 - C3	122.2(5)
C1 - C3	1.494(7)	C3 - C1 - C2	116.0(5)
C2 - N2	1.139(7)	N2 - C2 - C1	178.5(6)
O4 - N4	1.353(5)	C1 - C3 - O2	116.7(5)
N4 - C8	1.282(7)	C1 - C3 - N3	119.6(5)
		O2 - C3 - N3	123.6(5)
		O4 - N4 - C8	114.8(4)
		N4 - C8 - C9	123.1(5)
		N4 - C8 - C10	122.0(5)
		C9 - C8 - C10	114.5(5)
		N5 - C9 - C8	179.0(6)
		C8 - C10 - O5	116.7(5)
		C8 - C10 - N6	120.2(5)
		N6 - C10 - O5	123.0(5)
Metal Center:			
Sb1 - O1	2.114(3)	C17 - Sb1 - C16	120.5(2)
Sb1 - O4	2.123(3)	C17 - Sb1 - C15	120.1(2)
Sb1 - C15	2.105(6)	C16 - Sb1 - C15	119.4(3)
Sb1 - C16	2.094(5)	C17 - Sb1 - O1	90.04(17)
Sb1 - C17	2.085(5)	C16 - Sb1 - O1	93.21(19)
		C15 - Sb1 - O1	85.20(2)
		C17 - Sb1 - O4	88.64(17)
		C16 - Sb1 - O4	90.83(19)
		C15 - Sb1 - O4	92.10(2)
		O1 - Sb1 - O4	175.9(13)

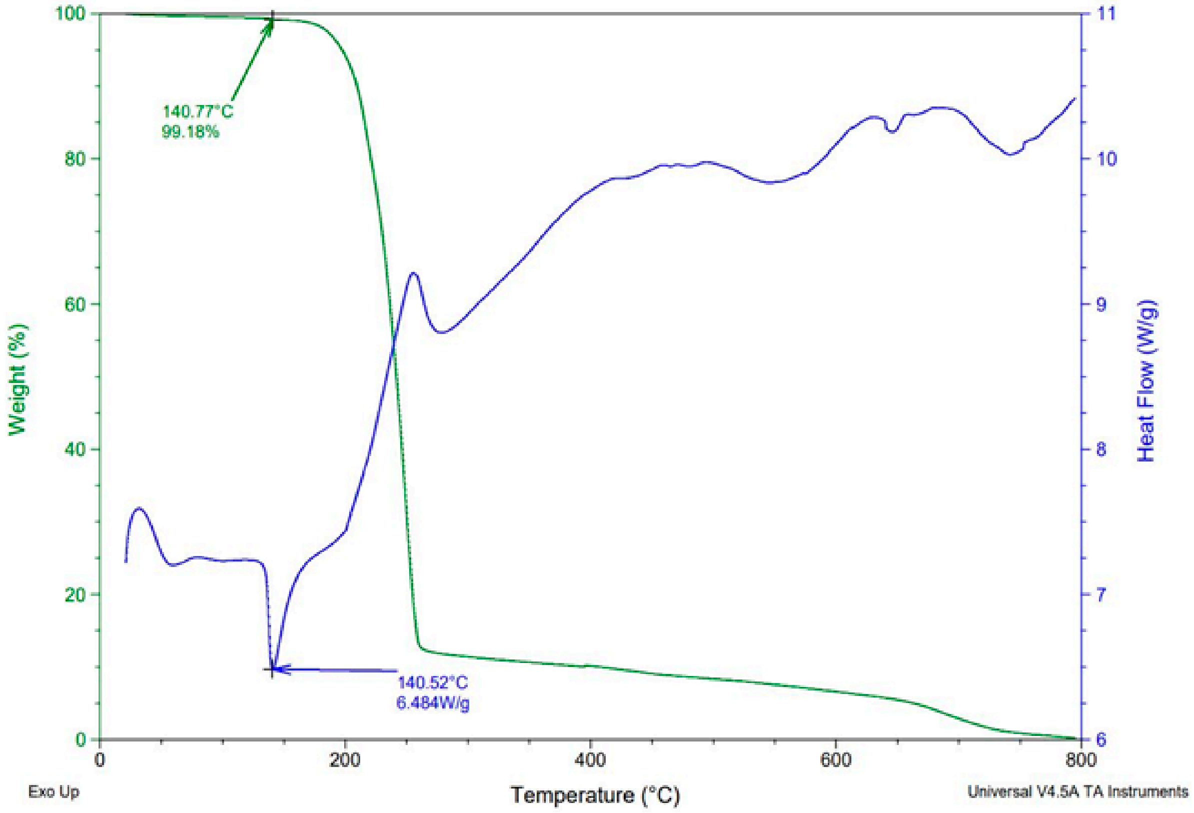

Thermal analysis traces for $SbMe_3(ECO)_2$: panoramic view. Melting point at 140.52 ° and decomposition~170°C. Green line indicates the weight loss and blue line indicates heat flow.

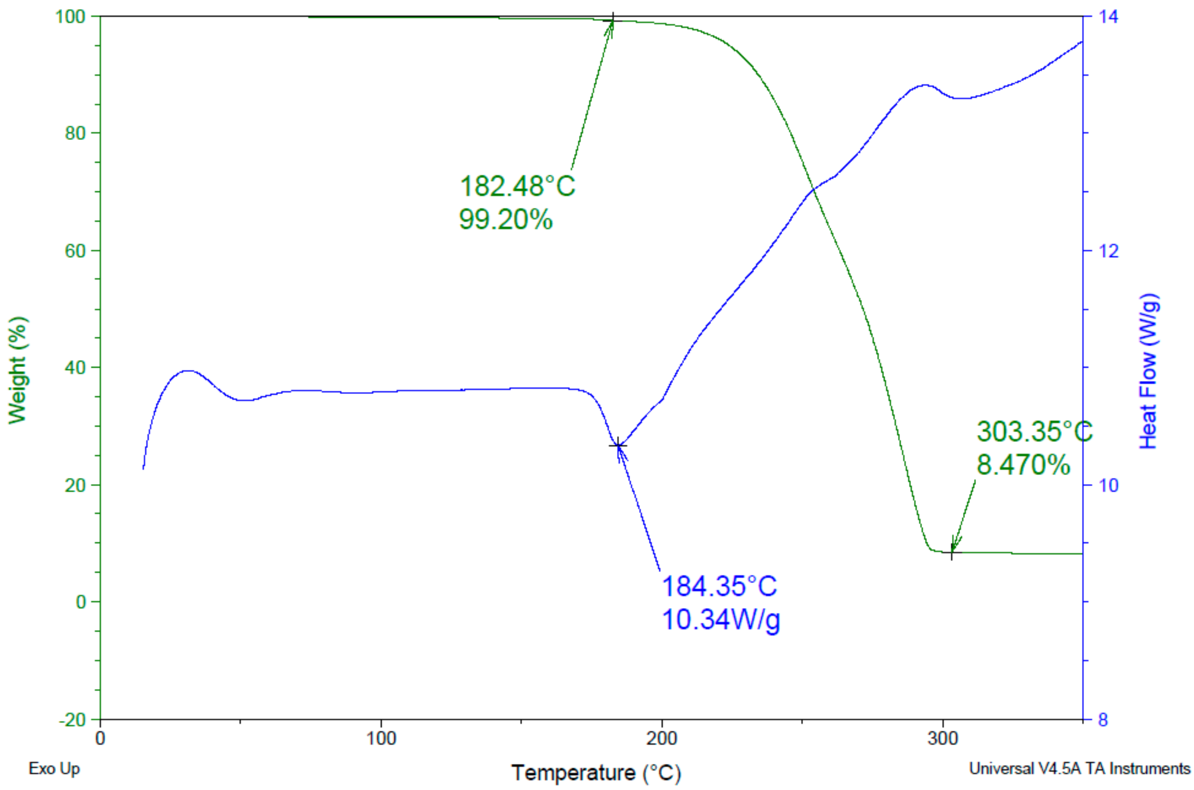

Thermal analysis traces for $SbMe_3(2,6\text{-diCl-PhCO})_2$: panoramic view. Melting point at 184.35 ° and decomposition~220°C. Green line indicates the weight loss and blue line indicates heat flow.

Table S15. Elemental analysis, yield and color of initial compounds, ligands, and antimony cyanoximates

Compound	Melting point (°C)	Yield (%)	Elemental Analysis, Element %		
			C, % Calc. (Found)	H, % Calc. (Found)	N, % Calc. (Found)
H(2-Cl-PhCO)	124.43	30	N/A	N/A	N/A
H(4-Cl-PhCO)	95	22	N/A	N/A	N/A
H(2,4-diCl-PhCO)	133.51	73	N/A	N/A	N/A
H(2,6-diCl-PhCO)	99.61	60	44.68 (44.75)	1.87 (1.94)	13.03 (13.12)
Tl(2-Cl-PhCO)	N/A	36.6	25.02 (25.30)	1.05 (0.88)	7.30 (7.18)
Tl(4-Cl-PhCO)	N/A	64.6	25.02 (25.91)	1.05 (0.92)	7.30 (7.26)
Tl(2,4-diCl-PhCO)	N/A	64.4	22.96 (24.66)	0.72 (0.67)	6.70 (6.86)
Tl(2,6-diCl-PhCO)	N/A	19.2	22.96 (22.19)	0.72 (0.64)	6.70 (6.36)
$SbMe_3$(2-Cl-PhCO)$_2$	128.78	80.3	N/A	N/A	N/A
$SbMe_3$(4-Cl-PhCO)$_2$	162.27	44.2	43.38 (43.33)	3.26 (3.35)	10.65 (10.39)
$SbMe_3$(2,4-diCl-PhCO)$_2$	129.18	74.0	38.36 (38.07)	2.54 (2.43)	9.42 (9.35)
$SbMe_3$(2,6-diCl-PhCO)$_2$	184.35	71.0	N/A	N/A	N/A
$SbMe_3$(TCO)$_2$	186.9 - 189.6	10.4	N/A	N/A	N/A
$SbMe_3$(TDCO)$_2$	175.27	41.5	32.58 (32.77)	4.42 (4.36)	17.54 (17.47)
$SbMe_3$(ACO)$_2$	N/A	8.0	N/A	N/A	N/A
$SbMe_3$(ECO)$_2$	140.52	80.8	N/A	N/A	N/A
$SbMe_3$(MCO)$_2$	118.5 - 120.9	77.3	N/A	N/A	N/A

* N/A = sample data was not recorded.

The crystal structure of $SbMe_3Br_2$, key starting material for all trimethyl-antimony bis-cyanoximates.

All the antimony cyanoximates were made from $SbMe_3Br_2$ which has pentacoordinated pnictogen atom in formal oxidation state +5. Compound represents white crystalline substance soluble in common organic solvents and hydrolyzes in water to a mixture of hydrated products including polynuclear species. Covalently attached bromine atoms are prone to replacement by better nucleophiles and that makes $SbMe_3Br_2$ excellent starting compound for making new trimethyl-antimony(V) organometallic compounds. Despite the fact of earlier determination of the crystal structure in the '70, we performed re-determination of its structure using modern hardware and processing software. Obtained data are much better than previously reported. Crystallographic community encourages chemists to improve quality of old deposited into Cambridge Crystal Structures Datacenter (CCDC) by deposition better structures into the database.

The crystallographic data for $SbMe_3Br_2$ is shown in Table S8. This compound crystallizes in hexagonal space group P 6_3/mmc with C3 being the principal axis in the molecule. The ASU unit in Figure XA. The $SbMe_3Br_2$ represents a compound with D_{3h} point group symmetry of non-H atoms with a 3-fold principal axis, 2-fold axes and a set of mirror planes. Figure XB shows multiple positions for H-atoms originated from their Site Occupancy Factor (SOF) for each H-atom is 0.25 because of their hosting C-atom occupying the mirror plane. For each hydrogen to have SOF = 1, there must be 4 H-atoms with SOF 0.25. Thus, there are 3 hydrogen atoms distributed between four positions totaling in occupancy to 1 and attached to the hosting single carbon atom generating 12 H-atoms in total on the methyl carbon. Figure 2 shows bond lengths and valence angles in the structure of this important precursor for the rest of reported here trimethylantimony(V) cyanoximates.

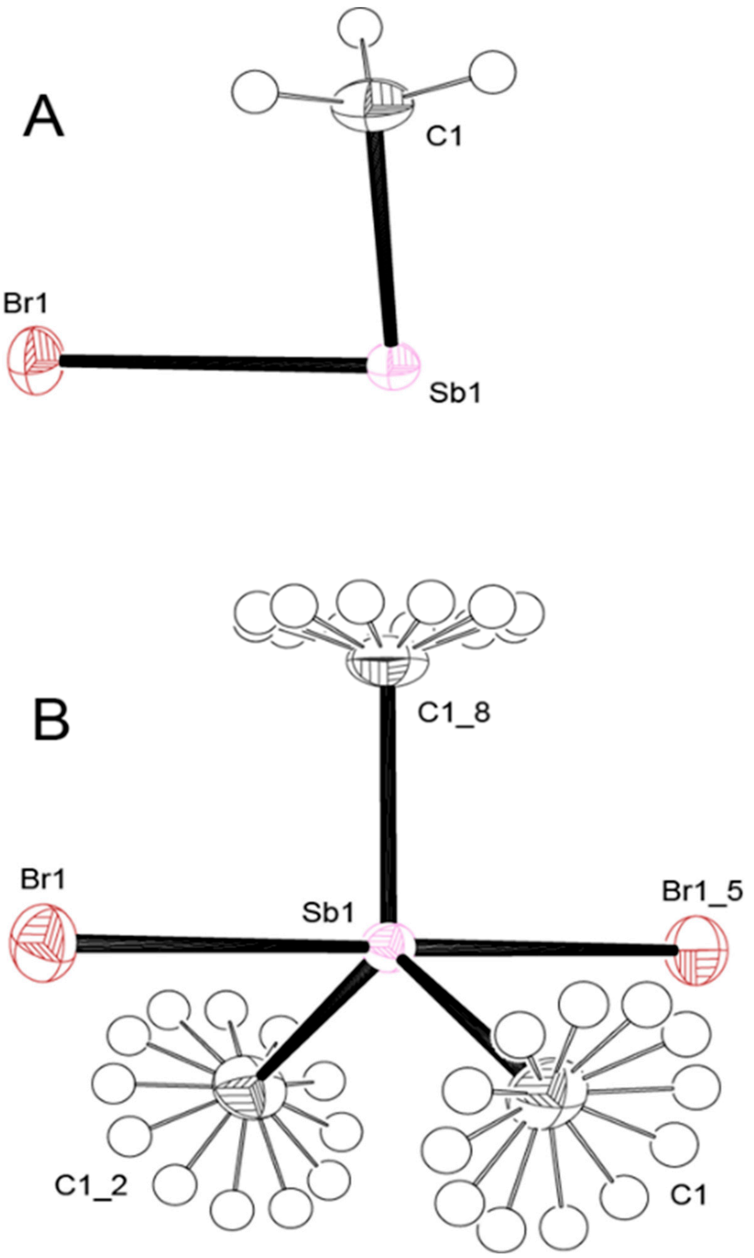

Figure S6. The molecular structure and numbering scheme for $SbMe_3Br_2$. The ASU in the structure (**A**), and the GROW fragment (**B**). Symmetry operations for positions: _2: -y, x-y, z; _5: y, -x-y, z+1/2; and _8: x-y, -y, -z.

Table S16. Crystal and refinement data for $SbMe_3Br_2$.

Empirical formula	$C_2H_6Br_{1.33}Sb$
Formula weight, g/mol	258.36
Temperature, K	120(2)
Wavelength, Å	0.71073 (Mo Kα)
Color, habitus	colorless needle
Crystal system	hexagonal
Space group	P 6_3/mmc
Unit cell dimensions, Å, °	a = 7.298(2) α = 90
	b = 7.298(2) β = 90
	c = 8.842(3) γ = 120
Unit cell volume, $Å^3$	407.8(3)
Z	3
Density (calculated), g/cm^3	3.156
Absorption coefficient, mm^{-1}	14.700
F (000)	347
Θ range, °	3.22 to 27.00°
Index ranges	$-9 \leq h \leq 9$
	$-9 \leq k \leq 9$
	$-11 \leq l \leq 11$
Reflections collected	4422
Independent reflections	200 [R_{int} = 0.0250]
Data / restraints / parameters	200 / 0 / 11
Goodness-of-fit on F^2	1.091
Final R indices [I>2σ(I)]	R1 = 0.0141
	wR2 = 0.0362
R indices [all data]	R1 = 0.0186
	wR2 = 0.0381
Largest diff. peak and hole, $eÅ^{-3}$	0.795 and -0.294

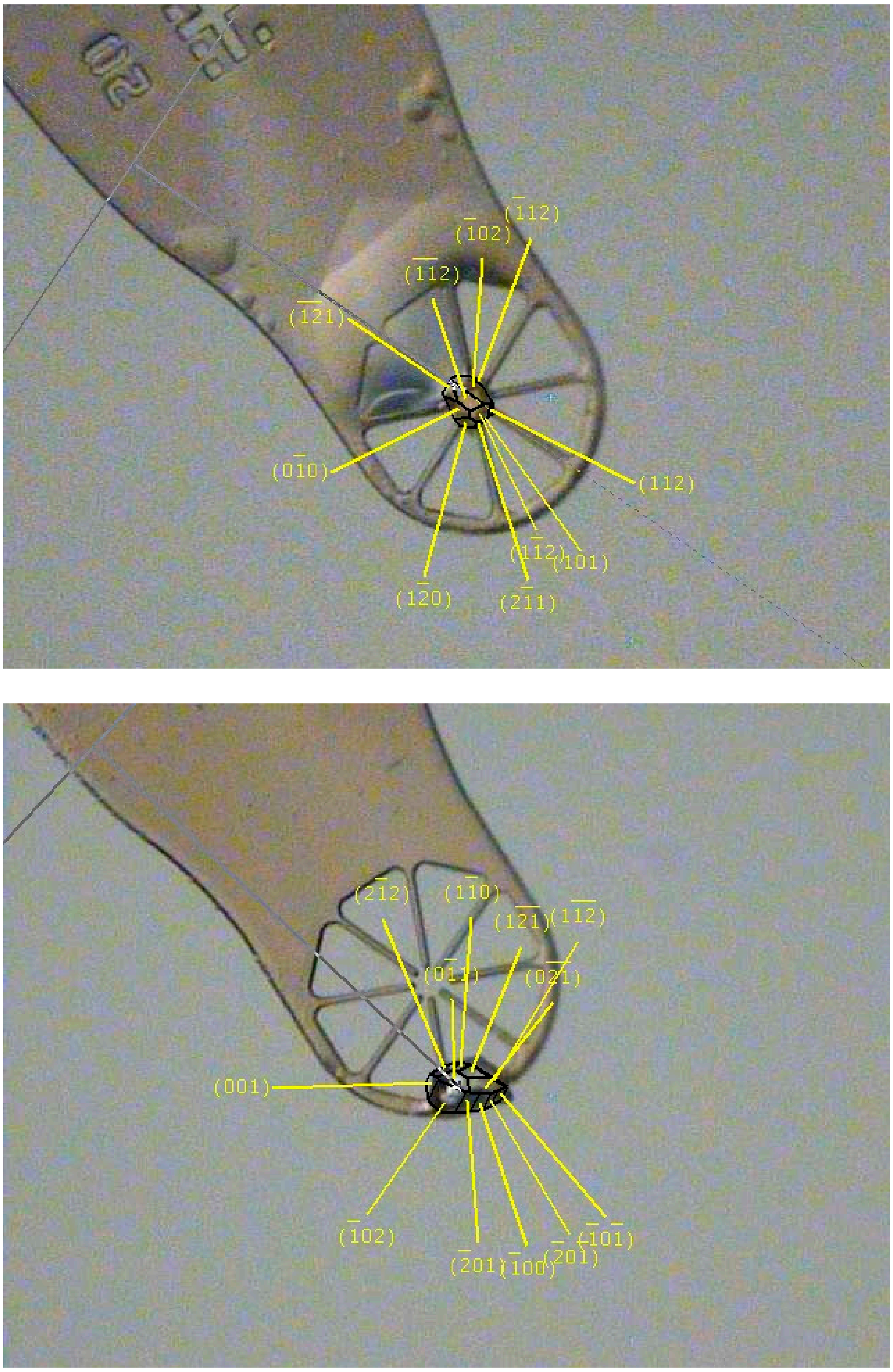

Crystal faces indexing was used for accurate measurements of crystals sizes and absorption correction. **A** – $SbMe_3(TCO)_2$; **B** – $SbMe_3(4Cl\text{-}PhCO)_2$. Both crystals in in MiTeGen loops.

Combined checkCIF reports for presented in this paper crystal structures.

----

checkCIF/PLATON (basic structural check)

Structure factors have been supplied for datablock(s) Sb-Me3-ACO2

No syntax errors found. CIF dictionary
Please wait while processing .... Interpreting this report
Structure factor report

Datablock: Sb-Me3-ACO2

Bond precision:	C-C = 0.0072 A		Wavelength=0.71073
Cell:	a=8.727(2)	b=8.829(2)	c=10.349(2)
	alpha=92.780(4)	beta=92.045(4)	gamma=107.665(4)
Temperature:	120 K		

	Calculated	Reported
Volume	757.8(3)	757.8(3)
Space group	P -1	P -1
Hall group	-P 1	-P 1
Moiety formula	C9 H13 N6 O4 Sb	C9 H13 N6 O4 Sb
Sum formula	C9 H13 N6 O4 Sb	C9 H13 N6 O4 Sb
Mr	391.01	391.91
Dx,g cm-3	1.714	1.660
Z	2	2
Mu (mm-1)	1.842	1.840
F000	384.0	359.0
F000'	382.97	
h,k,lmax	10,11,12	10,11,12
Nref	3108	3096
Tmin,Tmax	0.593,0.670	
Tmin'	0.557	

Correction method= Not given

Data completeness= 0.996 Theta(max)= 26.430

```
R(reflections)= 0.0446( 2373)        wR2(reflections)= 0.0864( 3096)
S = 0.996            Npar= 233
```

The following ALERTS were generated. Each ALERT has the format
test-name_ALERT_alert-type_alert-level.
Click on the hyperlinks for more details of the test.

Alert level C

DENSD01_ALERT_1_C The ratio of the submitted crystal density and that
calculated from the formula is outside the range 0.99 <> 1.01
Crystal density given = 1.660
Calculated crystal density = 1.717

PLAT043_ALERT_1_C Calculated and Reported Mol. Weight Differ by .. 0.89 Check
PLAT046_ALERT_1_C Reported Z, MW and D(calc) are Inconsistent .... 1.718 Check
PLAT057_ALERT_3_C Correction for Absorption Required RT(exp) ... 1.13 Do !
PLAT068_ALERT_1_C Reported F000 Differs from Calcd (or Missing)... Please Check
PLAT222_ALERT_3_C NonSolvent Resd 1 H Uiso(max)/Uiso(min) Range 10.0 Ratio
PLAT245_ALERT_2_C U(iso) H6A Smaller than U(eq) N6 by 0.020 Ang**2
PLAT250_ALERT_2_C Large U3/U1 Ratio for <U(i,j)> Tensor(Resd 1) 2.6 Note
PLAT350_ALERT_3_C Short C-H (X0.96,N1.08A) C7 - H7C . 0.79 Ang.

And 3 other PLAT350 Alerts

More ...

PLAT975_ALERT_2_C Check Calcd Resid. Dens. 0.81Ang From O1 . 0.45 eA-3
PLAT975_ALERT_2_C Check Calcd Resid. Dens. 0.70Ang From O4 . 0.43 eA-3

Alert level G

PLAT154_ALERT_1_G The s.u.'s on the Cell Angles are Equal ..(Note) 0.004 Degree
PLAT164_ALERT_4_G Nr. of Refined C-H H-Atoms in Heavy-Atom Struct. 9 Note
PLAT343_ALERT_2_G Unusual sp3 Angle Range in Main Residue for C7 Check
PLAT769_ALERT_4_G CIF Embedded Explicitly Supplied Scattering Data Please Note
PLAT883_ALERT_1_G No Info/Value for _atom_sites_solution_primary . Please Do !
PLAT912_ALERT_4_G Missing # of FCF Reflections Above STh/L= 0.600 12 Note
PLAT969_ALERT_5_G The 'Henn et al.' R-Factor-gap value .... 1.483 Note
Predicted wR2: Based on SigI**2 5.83 or SHELX Weight 8.68
PLAT978_ALERT_2_G Number C-C Bonds with Positive Residual Density. 2 Info
PLAT982_ALERT_1_G The Sb-f'= -0.6064 Deviates from IT-value = -0.5866 Check
PLAT983_ALERT_1_G The Sb-f"= 1.4876 Deviates from IT-Value = 1.5461 Check

---

0 **ALERT level A** = Most likely a serious problem - resolve or explain
0 **ALERT level B** = A potentially serious problem, consider carefully
14 **ALERT level C** = Check. Ensure it is not caused by an omission or oversight
10 **ALERT level G** = General information/check it is not something unexpected

8 ALERT type 1 CIF construction/syntax error, inconsistent or missing data
6 ALERT type 2 Indicator that the structure model may be wrong or deficient
6 ALERT type 3 Indicator that the structure quality may be low
3 ALERT type 4 Improvement, methodology, query or suggestion
1 ALERT type 5 Informative message, check

PLATON version of 15/07/2024; check.def file version of 15/07/2024

Datablock Sb-Me3-ACO2 - ellipsoid plot

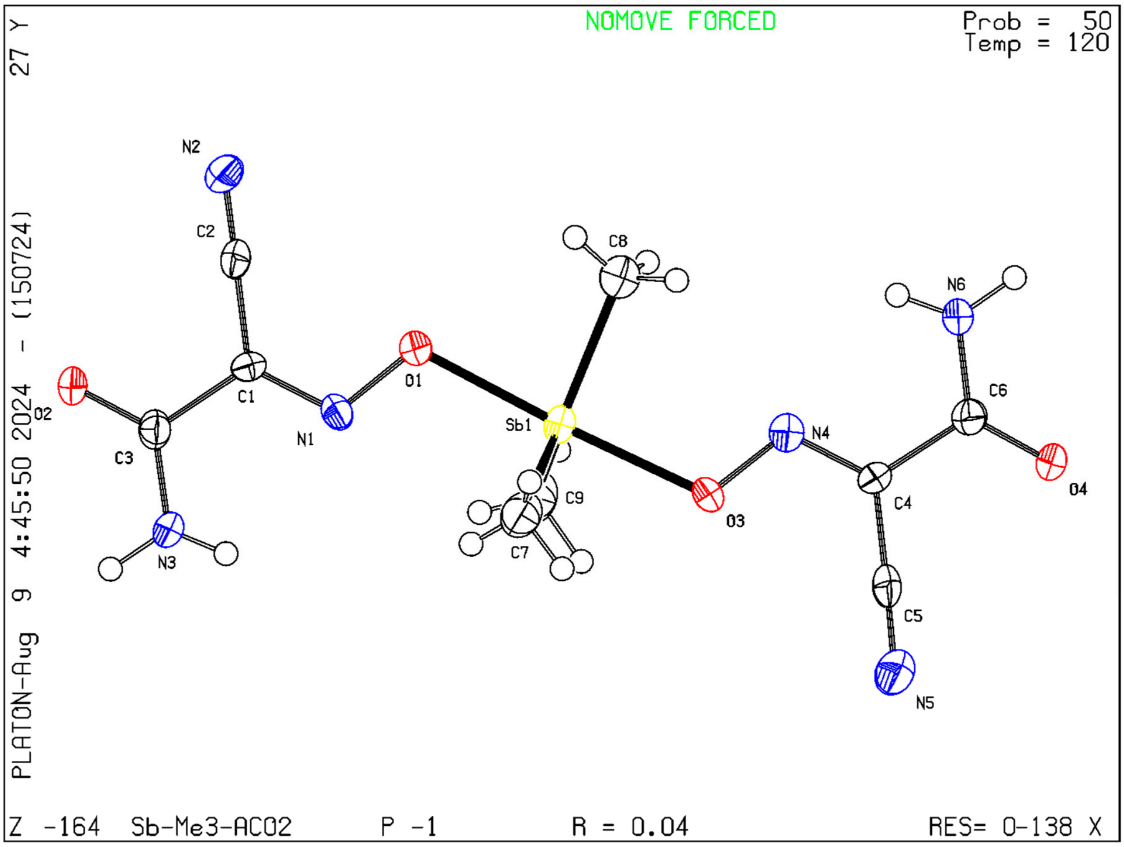

[Download CIF editor (publCIF) from the IUCr](#)
[Download CIF editor (enCIFer) from the CCDC](#)
[Test a new CIF entry](#)

checkCIF/PLATON (basic structural check)

Structure factors have been supplied for datablock(s) Sb-Me3-MCO2

No syntax errors found. CIF dictionary
Please wait while processing .... Interpreting this report
Structure factor report

Datablock: Sb-Me3-MCO2

Bond precision:	C-C = 0.0081 A	Wavelength=0.71073
Cell:	a=11.3901(19) b=12.602(2)	c=31.362(5)
	alpha=90 beta=90	gamma=90
Temperature: 120 K		
	Calculated	Reported
Volume	4501.6(13)	4501.5(13)
Space group	P b c a	P b c a
Hall group	-P 2ac 2ab	-P 2ac 2ab
Moiety formula	C17 H25 N6 O6 Sb	C17 H25 N6 O6 Sb
Sum formula	C17 H25 N6 O6 Sb	C17 H25 N6 O6 Sb
Mr	531.19	531.18
Dx,g cm-3	1.568	1.568
Z	8	8
Mu (mm-1)	1.270	1.269
F000	2144.0	2140.2
F000'	2140.18	
h,k,lmax	14,15,39	14,15,39
Nref	4634	4620
Tmin,Tmax	0.775,0.969	0.644,0.745
Tmin'	0.775	

Correction method= # Reported T Limits: Tmin=0.644 Tmax=0.745 AbsCorr = MULTI-SCAN

Data completeness= 0.997 Theta(max)= 26.430

R(reflections)= 0.0416(3056) wR2(reflections)= 0.1029(4620)

S = 1.031 Npar= 360

The following ALERTS were generated. Each ALERT has the format
test-name_ALERT_alert-type_alert-level.
Click on the hyperlinks for more details of the test.

Alert level C

ABSTY02_ALERT_1_C An _exptl_absorpt_correction_type has been given without
a literature citation. This should be contained in the
_exptl_absorpt_process_details field.
Absorption correction given as multi-scan

RINTA01_ALERT_3_C The value of Rint is greater than 0.12
Rint given 0.141

PLAT222_ALERT_3_C NonSolvent Resd 1 H Uiso(max)/Uiso(min) Range 6.7 Ratio

PLAT245_ALERT_2_C U(iso) H4B Smaller than U(eq) C4 by 0.019 Ang**2

And 5 other PLAT245 Alerts

More ...

PLAT342_ALERT_3_C Low Bond Precision on C-C Bonds .... 0.00812 Ang.

PLAT350_ALERT_3_C Short C-H (X0.96,N1.08A) C4 - H4B . 0.84 Ang.

And 2 other PLAT350 Alerts

More ...

Alert level G

PLAT002_ALERT_2_G Number of Distance or Angle Restraints on AtSite 2 Note
PLAT020_ALERT_3_G The Value of Rint is Greater Than 0.12 .... 0.141 Report
PLAT164_ALERT_4_G Nr. of Refined C-H H-Atoms in Heavy-Atom Struct. 22 Note
PLAT172_ALERT_4_G The CIF-Embedded .res File Contains DFIX Records 1 Report
PLAT343_ALERT_2_G Unusual sp3 Angle Range in Main Residue for C15 Check
PLAT398_ALERT_2_G Deviating C-O-C Angle From 120 for O6 . 109.7 Degree
PLAT769_ALERT_4_G CIF Embedded Explicitly Supplied Scattering Data Please Note
PLAT860_ALERT_3_G Number of Least-Squares Restraints .... 1 Note
PLAT883_ALERT_1_G No Info/Value for _atom_sites_solution_primary . Please Do !
PLAT910_ALERT_3_G Missing # of FCF Reflection(s) Below Theta(Min). 1 Note
0 0 2,
PLAT912_ALERT_4_G Missing # of FCF Reflections Above STh/L= 0.600 11 Note
PLAT933_ALERT_2_G Number of HKL-OMIT Records in Embedded .res File 1 Note
0 0 2,
PLAT960_ALERT_3_G Number of Intensities with I < - 2*sig(I) ... 10 Check
PLAT969_ALERT_5_G The 'Henn et al.' R-Factor-gap value .... 2.115 Note
Predicted wR2: Based on SigI**2 4.86 or SHELX Weight 9.98
PLAT978_ALERT_2_G Number C-C Bonds with Positive Residual Density. 0 Info
PLAT982_ALERT_1_G The Sb-f'= -0.6064 Deviates from IT-value = -0.5866 Check
PLAT983_ALERT_1_G The Sb-f"= 1.4876 Deviates from IT-Value = 1.5461 Check

---

0 **ALERT level A** = Most likely a serious problem - resolve or explain
0 **ALERT level B** = A potentially serious problem, consider carefully
13 **ALERT level C** = Check. Ensure it is not caused by an omission or oversight
17 **ALERT level G** = General information/check it is not something unexpected

4 ALERT type 1 CIF construction/syntax error, inconsistent or missing data
11 ALERT type 2 Indicator that the structure model may be wrong or deficient
10 ALERT type 3 Indicator that the structure quality may be low
4 ALERT type 4 Improvement, methodology, query or suggestion
1 ALERT type 5 Informative message, check

PLATON version of 15/07/2024; check.def file version of 15/07/2024

Datablock Sb-Me3-MCO2 - ellipsoid plot

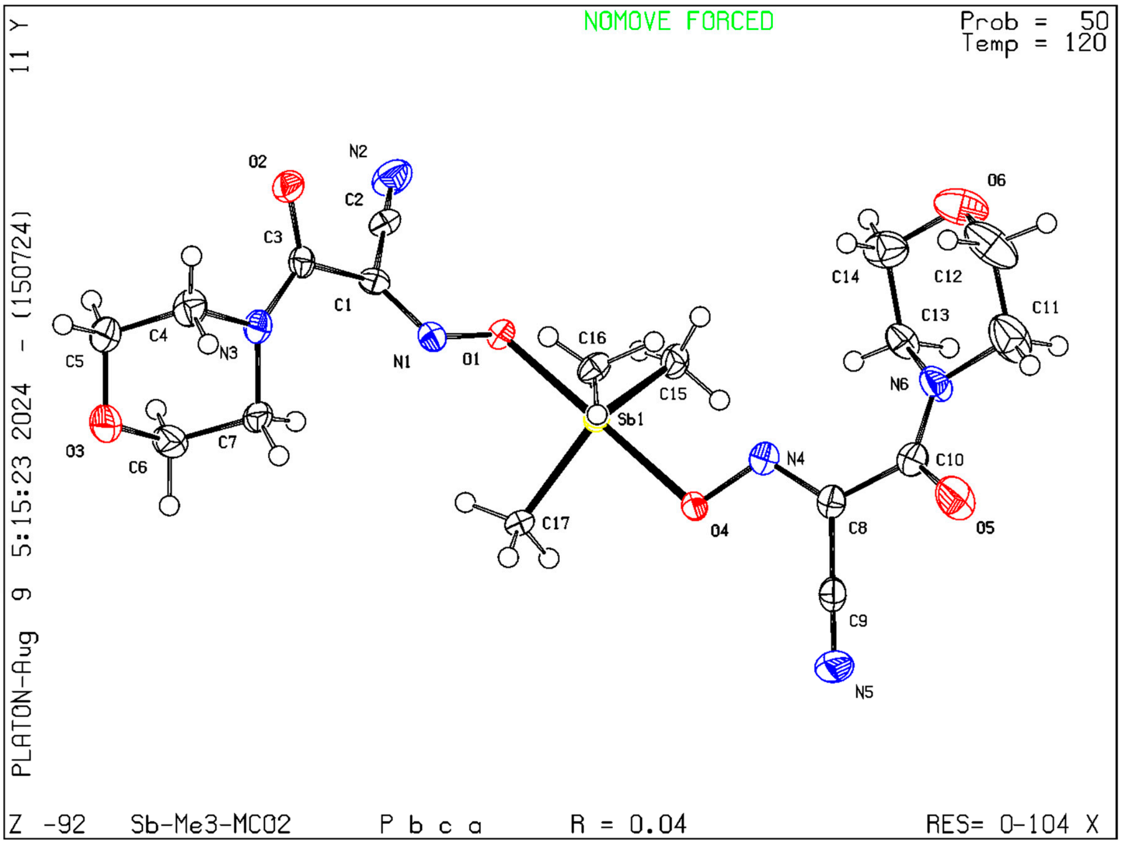

Download CIF editor (publCIF) from the IUCr
Download CIF editor (enCIFer) from the CCDC
Test a new CIF entry

checkCIF/PLATON (basic structural check)

Structure factors have been supplied for datablock(s) 2024-Sb-Me3-ECO2-RT

No syntax errors found. CIF dictionary
Please wait while processing .... Interpreting this report
Structure factor report

Datablock: 2024-Sb-Me3-ECO2-RT

```
Bond precision:      C-C = 0.0065 A               Wavelength=0.71073
```

Cell:	a=9.1401(5)	b=16.0912(9)	c=13.4416(8)
	alpha=90	beta=103.187(1)	gamma=90
Temperature:	296 K		

	Calculated	Reported
Volume	1924.80(19)	1924.80(19)
Space group	P 21/n	P 1 21/n 1
Hall group	-P 2yn	-P 2ybc (x-
Moiety formula	C13 H19 N4 O6 Sb	C13 H19 N4 O6 Sb
Sum formula	C13 H19 N4 O6 Sb	C13 H19 N4 O6 Sb
Mr	449.08	449.08
Dx,g cm-3	1.550	1.550
Z	4	4
Mu (mm-1)	1.466	1.466
F000	896.0	894.1
F000'	894.05	
h,k,lmax	10,19,15	10,19,15
Nref	3381	3374
Tmin,Tmax	0.720,0.765	0.674,0.746
Tmin'	0.661	

Correction method= # Reported T Limits: Tmin=0.674 Tmax=0.746 AbsCorr = MULTI-SCAN

Data completeness= 0.998 Theta(max)= 25.000

R(reflections)= 0.0269(2976) wR2(reflections)= 0.0744(3374)

S = 1.048 Npar= 216

The following ALERTS were generated. Each ALERT has the format
test-name_ALERT_alert-type_alert-level.
Click on the hyperlinks for more details of the test.

Alert level C

ABSTY02_ALERT_1_C An _exptl_absorpt_correction_type has been given without
a literature citation. This should be contained in the
_exptl_absorpt_process_details field.
Absorption correction given as multi-scan
PLAT126_ALERT_1_C Error in or Uninterpretable Hall Symbol .... -P 2YBC (X Check
PLAT230_ALERT_2_C Hirshfeld Test Diff for O6 --C9 . 6.0 s.u.
PLAT242_ALERT_2_C Low 'MainMol' Ueq as Compared to Neighbors of Sb1 Check
PLAT242_ALERT_2_C Low 'MainMol' Ueq as Compared to Neighbors of C3 Check
PLAT250_ALERT_2_C Large U3/U1 Ratio for <U(i,j)> Tensor(Resd 1) 2.3 Note
PLAT360_ALERT_2_C Short C(sp3)-C(sp3) Bond C4 - C5 . 1.39 Ang.
PLAT360_ALERT_2_C Short C(sp3)-C(sp3) Bond C9 - C10 . 1.36 Ang.
PLAT911_ALERT_3_C Missing FCF Refl Between Thmin & STh/L= 0.595 7 Report
-1 0 1, 2 0 2, 0 4 2, 0 0 4, 3 1 4, 0 3 4,
-2 4 4,

Alert level G

PLAT073_ALERT_1_G H-atoms ref, but _hydrogen_treatment Reported as constr Check
PLAT171_ALERT_4_G The CIF-Embedded .res File Contains EADP Records 1 Report

PLAT769_ALERT_4_G CIF Embedded Explicitly Supplied Scattering Data Please Note
PLAT883_ALERT_1_G No Info/Value for _atom_sites_solution_primary . Please Do !
PLAT909_ALERT_3_G Percentage of I>2sig(I) Data at Theta(Max) Still 77% Note
PLAT933_ALERT_2_G Number of HKL-OMIT Records in Embedded .res File 10 Note
-8 13 12, -1 0 1, 2 0 2, 0 4 2, 0 0 4, 3 1 4,
-11 11 2, 0 3 4, -2 4 4, 4 17 9,
PLAT960_ALERT_3_G Number of Intensities with I < - 2*sig(I) ... 5 Check
PLAT967_ALERT_5_G Note: Two-Theta Cutoff Value in Embedded .res .. 50.0 Degree
PLAT969_ALERT_5_G The 'Henn et al.' R-Factor-gap value .... 4.346 Note
Predicted wR2: Based on SigI**2 1.71 or SHELX Weight 7.10
PLAT978_ALERT_2_G Number C-C Bonds with Positive Residual Density. 2 Info
PLAT982_ALERT_1_G The Sb-f'= -0.6064 Deviates from IT-value = -0.5866 Check
PLAT983_ALERT_1_G The Sb-f"= 1.4876 Deviates from IT-Value = 1.5461 Check

---

0 **ALERT level A** = Most likely a serious problem - resolve or explain
0 **ALERT level B** = A potentially serious problem, consider carefully
9 **ALERT level C** = Check. Ensure it is not caused by an omission or oversight
12 **ALERT level G** = General information/check it is not something unexpected

6 ALERT type 1 CIF construction/syntax error, inconsistent or missing data
8 ALERT type 2 Indicator that the structure model may be wrong or deficient
3 ALERT type 3 Indicator that the structure quality may be low
2 ALERT type 4 Improvement, methodology, query or suggestion
2 ALERT type 5 Informative message, check

PLATON version of 15/07/2024; check.def file version of 15/07/2024

Datablock 2024-Sb-Me3-ECO2-RT - ellipsoid plot

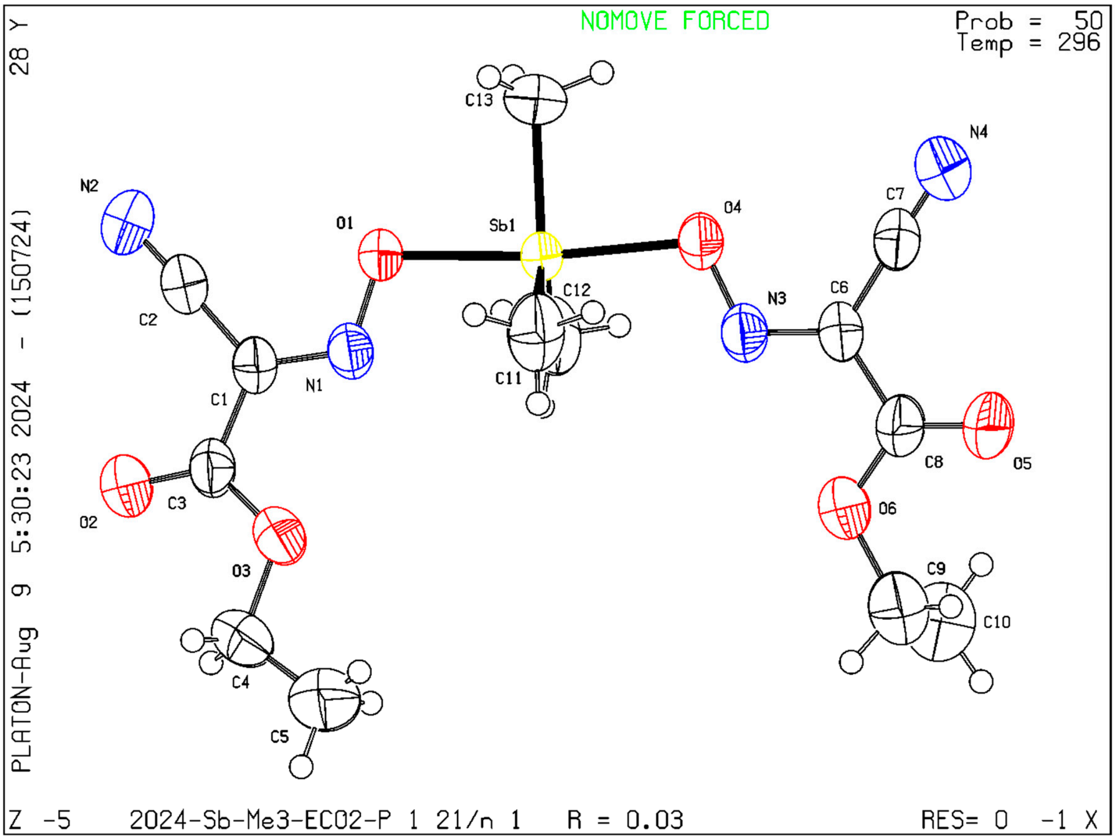

Download CIF editor (publCIF) from the IUCr
Download CIF editor (enCIFer) from the CCDC
Test a new CIF entry

checkCIF/PLATON (basic structural check)

Structure factors have been supplied for datablock(s) SbMe3-4Cl-PhCO2

THIS REPORT IS FOR GUIDANCE ONLY. IF USED AS PART OF A REVIEW PROCEDURE FOR PUBLICATION, IT SHOULD NOT REPLACE THE EXPERTISE OF AN EXPERIENCED CRYSTALLOGRAPHIC REFEREE.

No syntax errors found. CIF dictionary
Please wait while processing .... Interpreting this report

Structure factor report

Datablock: SbMe3-4Cl-PhCO2

Bond precision:	C-C = 0.0049 A		Wavelength=0.71073
Cell:	a=11.683(3)	b=7.2655(18)	c=25.126(6)
	alpha=90	beta=96.141(4)	gamma=90
Temperature:	120 K		

	Calculated	Reported
Volume	2120.5(9)	2120.6(9)
Space group	P 21/n	P 21/n
Hall group	-P 2yn	-P 2yn
Moiety formula	C19 H17 Cl2 N4 O2 Sb	C19 H17 Cl2 N4 O2 Sb
Sum formula	C19 H17 Cl2 N4 O2 Sb	C19 H17 Cl2 N4 O2 Sb
Mr	526.03	526.02
Dx,g cm-3	1.648	1.648
Z	4	4
Mu (mm-1)	1.575	1.575
F000	1040.0	1040.0
F000'	1039.14	
h,k,lmax	14,8,30	14,8,30
Nref	4150	4135
Tmin,Tmax	0.814,0.897	0.887,1.000
Tmin'	0.797	

Correction method= # Reported T Limits: Tmin=0.887 Tmax=1.000 AbsCorr = NUMERICAL

Data completeness= 0.996 Theta(max)= 26.000

R(reflections)= 0.0300(3316) wR2(reflections)= 0.0693(4135)

S = 1.036 Npar= 256

The following ALERTS were generated. Each ALERT has the format
test-name_ALERT_alert-type_alert-level.
Click on the hyperlinks for more details of the test.

Alert level C

PLAT911_ALERT_3_C Missing FCF Refl Between Thmin & STh/L= 0.600 13 Report
4 0 0, 1 1 1, -13 0 3, -14 0 4, 1 0 7, 2 0 8,
2 0 12, 3 0 13, 2 1 15, 3 0 17, 4 0 18, 4 0 20,
-5 0 27,
PLAT977_ALERT_2_C Check Negative Difference Density on H19A . -0.39 eA-3

Alert level G

PLAT883_ALERT_1_G No Info/Value for _atom_sites_solution_primary . Please Do !
PLAT910_ALERT_3_G Missing # of FCF Reflection(s) Below Theta(Min). 2 Note
-1 0 1, 0 0 2,
PLAT933_ALERT_2_G Number of HKL-OMIT Records in Embedded .res File 6 Note
4 0 20, -5 0 27, 4 0 18, -13 0 3, 4 0 0, -14 0 4,

PLAT941_ALERT_3_G Average HKL Measurement Multiplicity .... 4.3 Low
PLAT967_ALERT_5_G Note: Two-Theta Cutoff Value in Embedded .res .. 52.0 Degree
PLAT969_ALERT_5_G The 'Henn et al.' R-Factor-gap value .... 1.792 Note
Predicted wR2: Based on SigI**2 3.86 or SHELX Weight 6.68
PLAT978_ALERT_2_G Number C-C Bonds with Positive Residual Density. 4 Info

0 **ALERT level A** = Most likely a serious problem - resolve or explain
0 **ALERT level B** = A potentially serious problem, consider carefully
2 **ALERT level C** = Check. Ensure it is not caused by an omission or oversight
7 **ALERT level G** = General information/check it is not something unexpected

1 ALERT type 1 CIF construction/syntax error, inconsistent or missing data
3 ALERT type 2 Indicator that the structure model may be wrong or deficient
3 ALERT type 3 Indicator that the structure quality may be low
0 ALERT type 4 Improvement, methodology, query or suggestion
2 ALERT type 5 Informative message, check

PLATON version of 15/07/2024; check.def file version of 15/07/2024

Datablock SbMe3-4Cl-PhCO2 - ellipsoid plot

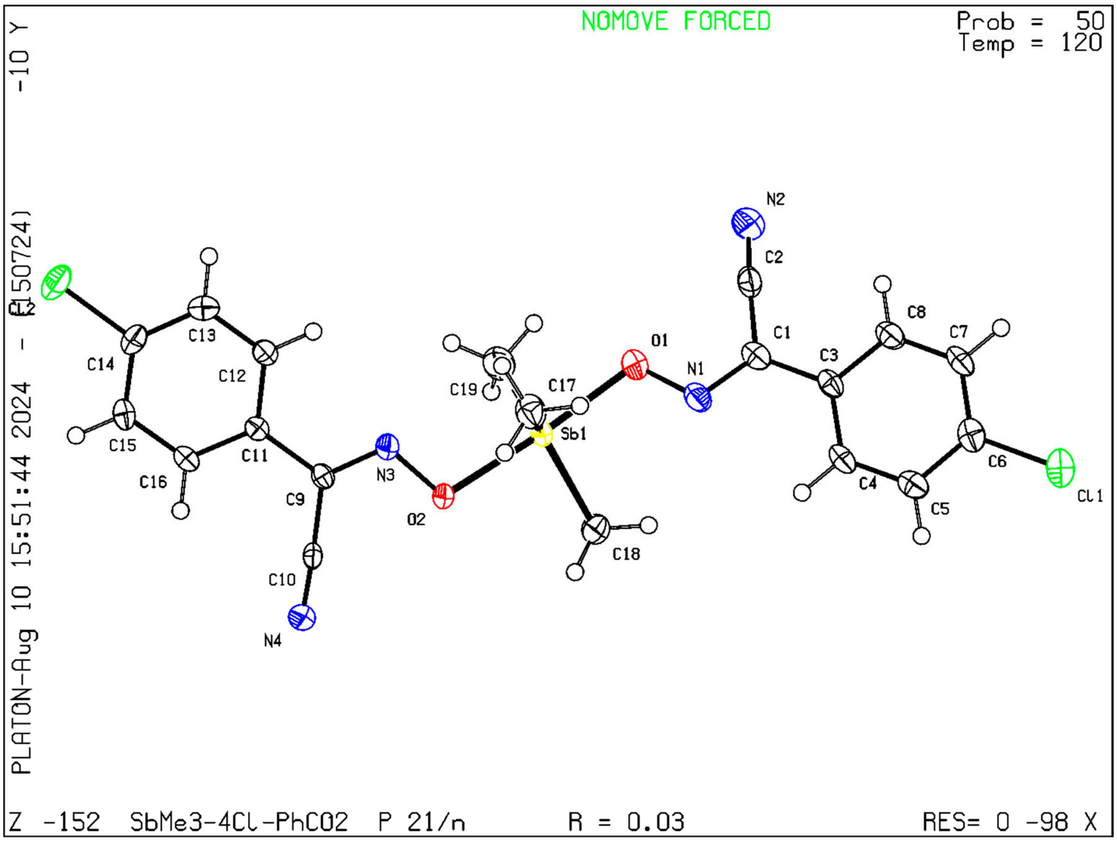

[Download CIF editor (publCIF) from the IUCr]
[Download CIF editor (enCIFer) from the CCDC]
[Test a new CIF entry]

checkCIF/PLATON (basic structural check)

Structure factors have been supplied for datablock(s) SbMe3-2,6-diCl-PhCO2

THIS REPORT IS FOR GUIDANCE ONLY. IF USED AS PART OF A REVIEW PROCEDURE FOR PUBLICATION, IT SHOULD NOT REPLACE THE EXPERTISE OF AN EXPERIENCED CRYSTALLOGRAPHIC REFEREE.

No syntax errors found. CIF dictionary
Please wait while processing .... Interpreting this report
Structure factor report

Datablock: SbMe3-2,6-diCl-PhCO2

Bond precision:	C-C = 0.0097 A		Wavelength=0.71073
Cell:	a=7.540(3)	b=10.889(4)	c=14.709(6)
	alpha=104.891(6)	beta=93.234(6)	gamma=100.956(6)
Temperature:	150 K		

	Calculated	Reported
Volume	1138.8(8)	1138.7(8)
Space group	P -1	P -1
Hall group	-P 1	-P 1
Moiety formula	C19 H15 Cl4 N4 O2 Sb	?
Sum formula	C19 H15 Cl4 N4 O2 Sb	C19 H15 Cl4 N4 O2 Sb
Mr	594.91	594.90
Dx,g cm-3	1.735	1.735
Z	2	2
Mu (mm-1)	1.704	1.704
F000	584.0	584.0
F000'	584.17	
h,k,lmax	9,13,18	9,13,18
Nref	4662	4636
Tmin,Tmax	0.836,0.886	0.584,0.745
Tmin'	0.836	

Correction method= # Reported T Limits: Tmin=0.584 Tmax=0.745 AbsCorr = MULTI-SCAN

Data completeness= 0.994 Theta(max)= 26.368

R(reflections)= 0.0541(3335) wR2(reflections)= 0.1376(4636)

S = 0.968 Npar= 274

The following ALERTS were generated. Each ALERT has the format
test-name_ALERT_alert-type_alert-level.
Click on the hyperlinks for more details of the test.

Alert level C

PLAT342_ALERT_3_C Low Bond Precision on C-C Bonds .... 0.00969 Ang.
PLAT911_ALERT_3_C Missing FCF Refl Between Thmin & STh/L= 0.600 9 Report
1 2 3, 1 3 4, 1 4 4, 1 4 5, 2 4 5, 1 5 5,
2 5 6, 0 9 7, -1 10 7,
PLAT971_ALERT_2_C Check Calcd Resid. Dens. 0.95Ang From Sb1 1.70 eA-3
PLAT972_ALERT_2_C Check Calcd Resid. Dens. 0.69Ang From Sb1 -1.69 eA-3
And 2 other PLAT972 Alerts
More ...
PLAT975_ALERT_2_C Check Calcd Resid. Dens. 1.03Ang From O1 . 0.58 eA-3
PLAT977_ALERT_2_C Check Negative Difference Density on H19C . -0.34 eA-3

Alert level G

PLAT154_ALERT_1_G The s.u.'s on the Cell Angles are Equal ..(Note) 0.006 Degree
PLAT480_ALERT_4_G Long H...A H-Bond Reported H13 ..CL2 . 2.99 Ang.
PLAT883_ALERT_1_G No Info/Value for _atom_sites_solution_primary . Please Do !
PLAT910_ALERT_3_G Missing # of FCF Reflection(s) Below Theta(Min). 1 Note
0 0 1,
PLAT912_ALERT_4_G Missing # of FCF Reflections Above STh/L= 0.600 16 Note
PLAT933_ALERT_2_G Number of HKL-OMIT Records in Embedded .res File 7 Note
1 3 4, 1 2 3, 2 5 6, 1 5 5, 2 4 5, 1 4 5,
1 4 4,
PLAT941_ALERT_3_G Average HKL Measurement Multiplicity .... 2.7 Low
PLAT969_ALERT_5_G The 'Henn et al.' R-Factor-gap value .... 1.828 Note
Predicted wR2: Based on SigI**2 7.53 or SHELX Weight 14.22
PLAT978_ALERT_2_G Number C-C Bonds with Positive Residual Density. 1 Info

0 **ALERT level A** = Most likely a serious problem - resolve or explain
0 **ALERT level B** = A potentially serious problem, consider carefully
8 **ALERT level C** = Check. Ensure it is not caused by an omission or oversight
9 **ALERT level G** = General information/check it is not something unexpected

2 ALERT type 1 CIF construction/syntax error, inconsistent or missing data
8 ALERT type 2 Indicator that the structure model may be wrong or deficient
4 ALERT type 3 Indicator that the structure quality may be low
2 ALERT type 4 Improvement, methodology, query or suggestion
1 ALERT type 5 Informative message, check

PLATON version of 15/07/2024; check.def file version of 15/07/2024

Datablock SbMe3-2,6-diCl-PhCO2 - ellipsoid plot

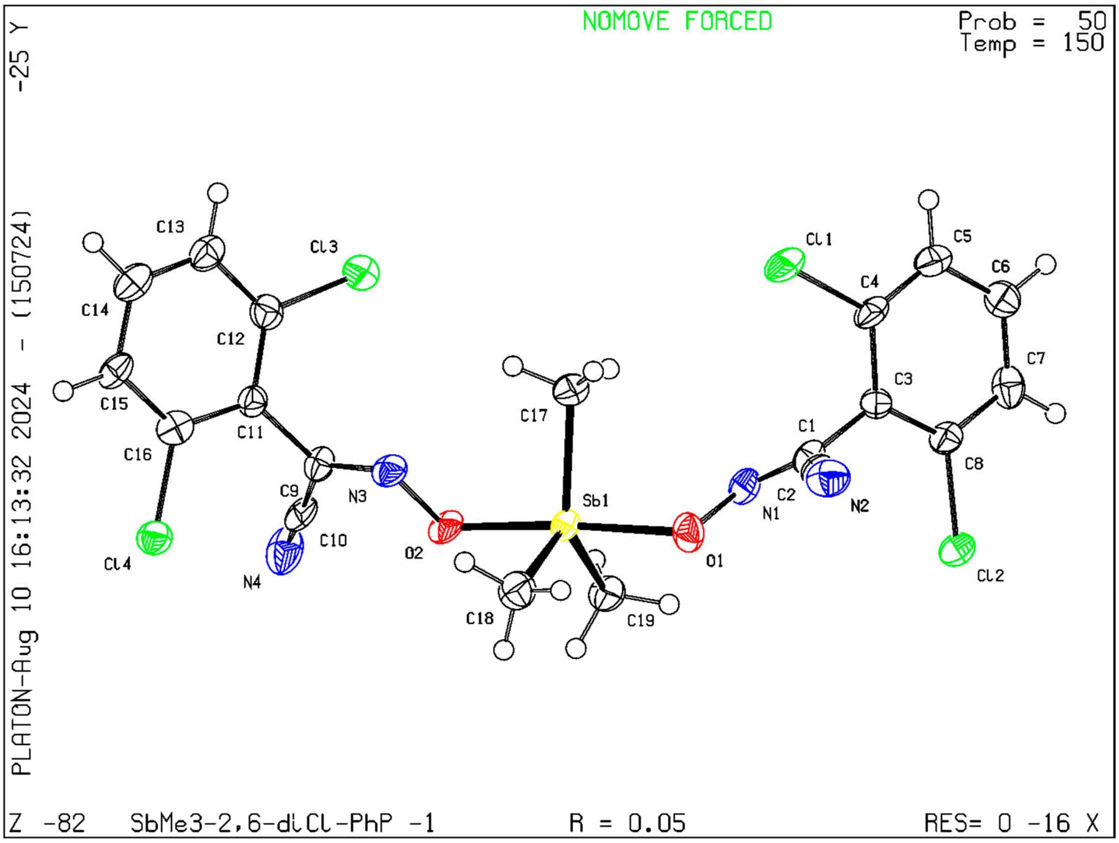

Download CIF editor (publCIF) from the IUCr
Download CIF editor (enCIFer) from the CCDC
Test a new CIF entry

checkCIF/PLATON (basic structural check)

Structure factors have been supplied for datablock(s) SbMe3-2,4-diCl-PhCO2

THIS REPORT IS FOR GUIDANCE ONLY. IF USED AS PART OF A REVIEW PROCEDURE FOR PUBLICATION, IT SHOULD NOT REPLACE THE EXPERTISE OF AN EXPERIENCED CRYSTALLOGRAPHIC REFEREE.

No syntax errors found. CIF dictionary
Please wait while processing .... Interpreting this report
Structure factor report

Datablock: SbMe3-2,4-diCl-PhCO2

Bond precision:	C-C = 0.0186 A		Wavelength=0.71073
Cell:	a=7.1216(19)	b=23.813(6)	c=13.847(4)
	alpha=90	beta=102.966(5)	gamma=90
Temperature:	120 K		

	Calculated	Reported
Volume	2288.4(11)	2288.4(10)
Space group	P 21/c	P 21/c
Hall group	-P 2ybc	-P 2ybc
Moiety formula	C19 H15 Cl4 N4 O2 Sb	C19 H15 Cl4 N4 O2 Sb
Sum formula	C19 H15 Cl4 N4 O2 Sb	C19 H15 Cl4 N4 O2 Sb
Mr	594.91	594.90
Dx,g cm-3	1.727	1.727
Z	4	4
Mu (mm-1)	1.696	1.696
F000	1168.0	1168.0
F000'	1168.34	
h,k,lmax	8,28,16	0,0,0
Nref	4032	4026
Tmin,Tmax	0.828,0.893	0.694,0.873
Tmin'	0.827	

Correction method= # Reported T Limits: Tmin=0.694 Tmax=0.873 AbsCorr = MULTI-SCAN

Data completeness= 0.999 Theta(max)= 24.999

R(reflections)= 0.0873(2761) wR2(reflections)= 0.1804(4026)

S = 1.101 Npar= 268

The following ALERTS were generated. Each ALERT has the format
test-name_ALERT_alert-type_alert-level.
Click on the hyperlinks for more details of the test.

Alert level C

PLAT213_ALERT_2_C Atom C12 has ADP max/min Ratio ..... 3.3 prolat
PLAT230_ALERT_2_C Hirshfeld Test Diff for N3 --C12 . 5.9 s.u.
PLAT232_ALERT_2_C Hirshfeld Test Diff (M-X) Sb1 --O2 . 7.2 s.u.
PLAT234_ALERT_4_C Large Hirshfeld Difference O2 --N3 . 0.17 Ang.
And 3 other PLAT234 Alerts
More ...
PLAT242_ALERT_2_C Low 'MainMol' Ueq as Compared to Neighbors of C13 Check
PLAT342_ALERT_3_C Low Bond Precision on C-C Bonds .... 0.01862 Ang.
PLAT906_ALERT_3_C Large K Value in the Analysis of Variance ...... 27.560 Check
PLAT906_ALERT_3_C Large K Value in the Analysis of Variance ...... 3.900 Check
PLAT911_ALERT_3_C Missing FCF Refl Between Thmin & STh/L= 0.595 6 Report
7 5 0, 1 16 0, -5 15 3, 2 15 5, -6 2 7, -7 3 8,
PLAT973_ALERT_2_C Check Calcd Positive Resid. Density on Sb1 1.02 eA-3
PLAT977_ALERT_2_C Check Negative Difference Density on H9B . -0.38 eA-3
PLAT977_ALERT_2_C Check Negative Difference Density on H16 . -0.35 eA-3

Alert level G

PLAT083_ALERT_2_G SHELXL Second Parameter in WGHT Unusually Large   60.91 Why ?
PLAT171_ALERT_4_G The CIF-Embedded .res File Contains EADP Records   1 Report
PLAT434_ALERT_2_G Short Inter HL..HL Contact Cl1   ..Cl4   .   3.39 Ang.
1-x,1-y,1-z =   3_666 Check
PLAT480_ALERT_4_G Long H...A H-Bond Reported H18   ..CL4   .   2.94 Ang.

And 26 other PLAT480 Alerts

More ...

PLAT883_ALERT_1_G No Info/Value for _atom_sites_solution_primary .   Please Do !
PLAT909_ALERT_3_G Percentage of I>2sig(I) Data at Theta(Max) Still   41% Note
PLAT910_ALERT_3_G Missing # of FCF Reflection(s) Below Theta(Min).   1 Note
0 2 0,
PLAT933_ALERT_2_G Number of HKL-OMIT Records in Embedded .res File   6 Note
-5 15 3, -6 2 7,   1 16 0, -7 3 8,   2 15 5,   7 5 0,
PLAT941_ALERT_3_G Average HKL Measurement Multiplicity ....   1.0 Low
PLAT961_ALERT_5_G Dataset Contains no Negative Intensities ....   Please Check
PLAT965_ALERT_2_G The SHELXL WEIGHT Optimisation has not Converged   Please Check
PLAT967_ALERT_5_G Note: Two-Theta Cutoff Value in Embedded .res ..   50.0 Degree
PLAT969_ALERT_5_G The 'Henn et al.' R-Factor-gap value ....   3.592 Note
Predicted wR2: Based on SigI**2 5.02 or SHELX Weight 16.38
PLAT978_ALERT_2_G Number C-C Bonds with Positive Residual Density.   0 Info

---

0 **ALERT level A** = Most likely a serious problem - resolve or explain
0 **ALERT level B** = A potentially serious problem, consider carefully
15 **ALERT level C** = Check. Ensure it is not caused by an omission or oversight
40 **ALERT level G** = General information/check it is not something unexpected

1 ALERT type 1 CIF construction/syntax error, inconsistent or missing data
12 ALERT type 2 Indicator that the structure model may be wrong or deficient
7 ALERT type 3 Indicator that the structure quality may be low
32 ALERT type 4 Improvement, methodology, query or suggestion
3 ALERT type 5 Informative message, check

PLATON version of 15/07/2024; check.def file version of 15/07/2024

Datablock SbMe3-2,4-diCl-PhCO2 - ellipsoid plot

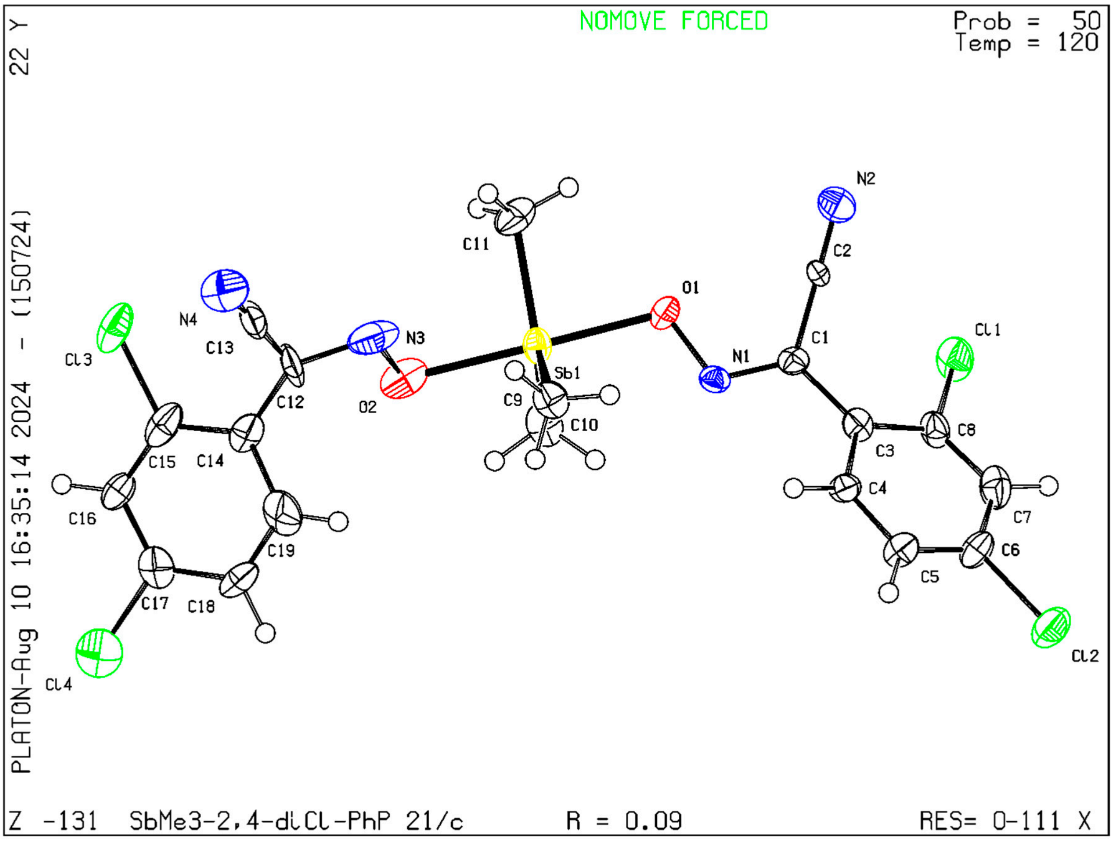

Download CIF editor (publCIF) from the IUCr
Download CIF editor (enCIFer) from the CCDC
Test a new CIF entry

checkCIF/PLATON (basic structural check)

Structure factors have been supplied for datablock(s) SbMe3-TCO2

THIS REPORT IS FOR GUIDANCE ONLY. IF USED AS PART OF A REVIEW PROCEDURE FOR PUBLICATION, IT SHOULD NOT REPLACE THE EXPERTISE OF AN EXPERIENCED CRYSTALLOGRAPHIC REFEREE.

No syntax errors found. CIF dictionary
Please wait while processing .... Interpreting this report
Structure factor report

Datablock: SbMe3-TCO2

Bond precision:	C-C = 0.0125 A		Wavelength=0.71073
Cell:	a=14.6219(17)	b=16.0812(18)	c=7.1201(8)
	alpha=90	beta=90	gamma=90
Temperature: 296 K			

	Calculated	Reported
Volume	1674.2(3)	1674.2(3)
Space group	P n m a	P n m a
Hall group	-P 2ac 2n	-P 2ac 2n
Moiety formula	C9 H13 N6 O2 S2 Sb	C9 H13 N6 O2 S2 Sb
Sum formula	C9 H13 N6 O2 S2 Sb	C9 H13 N6 O2 S2 Sb
Mr	423.13	423.12
Dx,g cm-3	1.679	1.679
Z	4	4
Mu (mm-1)	1.906	1.906
F000	832.0	832.0
F000'	830.86	
h,k,lmax	17,19,8	17,19,8
Nref	1532	1532
Tmin,Tmax	0.900,0.928	0.941,1.000
Tmin'	0.895	

Correction method= # Reported T Limits: Tmin=0.941 Tmax=1.000 AbsCorr = NUMERICAL

Data completeness= 1.000 Theta(max)= 24.999

R(reflections)= 0.0639(1098) wR2(reflections)= 0.1653(1532)

S = 1.126 Npar= 100

The following ALERTS were generated. Each ALERT has the format
test-name_ALERT_alert-type_alert-level.
Click on the hyperlinks for more details of the test.

Alert level A

PLAT971_ALERT_2_A Check Calcd Resid. Dens. 1.05Ang From Sb1 4.33 eA-3

Alert level B

PLAT094_ALERT_2_B Ratio of Maximum / Minimum Residual Density .... 4.31 Report

---

Alert level C

DIFMX02_ALERT_1_C The maximum difference density is > 0.1*ZMAX*0.75
The relevant atom site should be identified.
PLAT097_ALERT_2_C Large Reported Max. (Positive) Residual Density 4.40 eA-3
PLAT234_ALERT_4_C Large Hirshfeld Difference Sb1 --C6 . 0.16 Ang.
PLAT234_ALERT_4_C Large Hirshfeld Difference C1 --C2 . 0.17 Ang.
PLAT342_ALERT_3_C Low Bond Precision on C-C Bonds .... 0.0125 Ang.
PLAT906_ALERT_3_C Large K Value in the Analysis of Variance ...... 12.033 Check
PLAT906_ALERT_3_C Large K Value in the Analysis of Variance ...... 2.121 Check

---

Alert level G

PLAT007_ALERT_5_G Number of Unrefined Donor-H Atoms .... 2 Report
H3A H3B
PLAT083_ALERT_2_G SHELXL Second Parameter in WGHT Unusually Large 17.72 Why ?
PLAT299_ALERT_4_G Atom Site Occupancy Constrained at .... 0.5 Check
H4A H4B H4C H5A H5B H5C H6A H6B
H6C
PLAT883_ALERT_1_G No Info/Value for _atom_sites_solution_primary . Please Do !
PLAT909_ALERT_3_G Percentage of I>2sig(I) Data at Theta(Max) Still 45% Note
PLAT954_ALERT_1_G Reported (CIF) and Actual (FCF) Kmax Differ by . 1 Units
PLAT965_ALERT_2_G The SHELXL WEIGHT Optimisation has not Converged Please Check
PLAT967_ALERT_5_G Note: Two-Theta Cutoff Value in Embedded .res .. 50.0 Degree
PLAT969_ALERT_5_G The 'Henn et al.' R-Factor-gap value .... 6.198 Note
Predicted wR2: Based on SigI**2 2.67 or SHELX Weight 14.68
PLAT978_ALERT_2_G Number C-C Bonds with Positive Residual Density. 2 Info

---

1 **ALERT level A** = Most likely a serious problem - resolve or explain
1 **ALERT level B** = A potentially serious problem, consider carefully
7 **ALERT level C** = Check. Ensure it is not caused by an omission or oversight
10 **ALERT level G** = General information/check it is not something unexpected

3 ALERT type 1 CIF construction/syntax error, inconsistent or missing data
6 ALERT type 2 Indicator that the structure model may be wrong or deficient
4 ALERT type 3 Indicator that the structure quality may be low
3 ALERT type 4 Improvement, methodology, query or suggestion
3 ALERT type 5 Informative message, check

PLATON version of 15/07/2024; check.def file version of 15/07/2024

Datablock SbMe3-TCO2 - ellipsoid plot

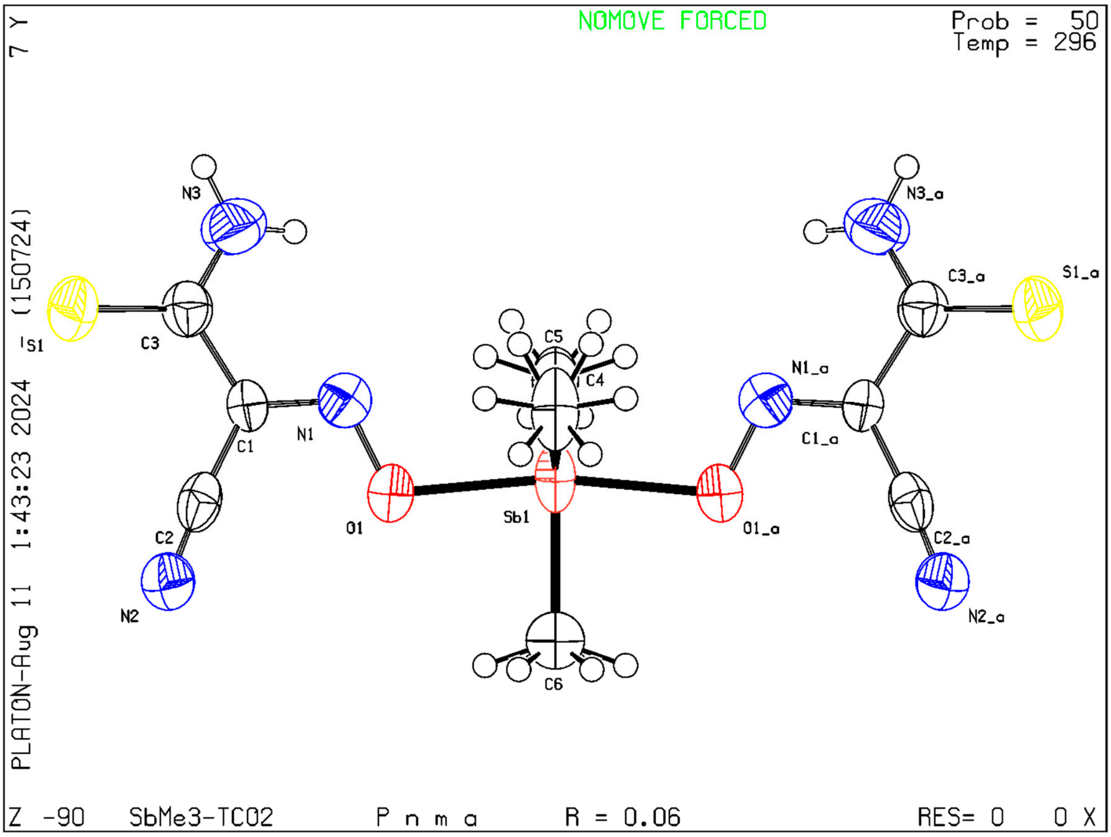

Download CIF editor (publCIF) from the IUCr
Download CIF editor (enCIFer) from the CCDC
Test a new CIF entry

checkCIF/PLATON (basic structural check)

Structure factors have been supplied for datablock(s) SbMe3-TDCO2

No syntax errors found. CIF dictionary
Please wait while processing .... Interpreting this report
Structure factor report

Datablock: SbMe3-TDCO2

Bond precision:	C-C = 0.0090 A			Wavelength=0.71073
Cell:	a=13.4229(13)	b=12.1231(11)	c=12.9825(12)	
	alpha=90	beta=103.248(2)	gamma=90	

Temperature: 296 K

	Calculated	Reported
Volume	2056.4(3)	2056.4(3)
Space group	P 21/c	P 21/c
Hall group	-P 2ybc	-P 2ybc
Moiety formula	C13 H21 N6 O2 S2 Sb	C13 H21 N6 O2 S2 Sb
Sum formula	C13 H21 N6 O2 S2 Sb	C13 H21 N6 O2 S2 Sb
Mr	479.24	479.23
Dx,g cm-3	1.548	1.548
Z	4	4
Mu (mm-1)	1.562	1.562
F000	960.0	960.0
F000'	958.89	
h,k,lmax	16,15,16	16,15,16
Nref	4225	4221
Tmin,Tmax	0.730,0.792	0.683,0.746
Tmin'	0.712	

Correction method= # Reported T Limits: Tmin=0.683 Tmax=0.746 AbsCorr = ?

Data completeness= 0.999 Theta(max)= 26.417

R(reflections)= 0.0514(2921) wR2(reflections)= 0.1130(4221)

S = 1.031 Npar= 231

The following ALERTS were generated. Each ALERT has the format
test-name_ALERT_alert-type_alert-level.
Click on the hyperlinks for more details of the test.

Alert level C

PLAT052_ALERT_1_C Info on Absorption Correction Method Not Given Please Do !
PLAT342_ALERT_3_C Low Bond Precision on C-C Bonds .... 0.009 Ang.
PLAT906_ALERT_3_C Large K Value in the Analysis of Variance ...... 4.089 Check
PLAT971_ALERT_2_C Check Calcd Resid. Dens. 0.88Ang From Sb1 1.68 eA-3

Alert level G

PLAT083_ALERT_2_G SHELXL Second Parameter in WGHT Unusually Large 6.21 Why ?
PLAT171_ALERT_4_G The CIF-Embedded .res File Contains EADP Records 2 Report
PLAT301_ALERT_3_G Main Residue Disorder ....(Resd 1) 8% Note
PLAT883_ALERT_1_G No Info/Value for _atom_sites_solution_primary . Please Do !
PLAT912_ALERT_4_G Missing # of FCF Reflections Above STh/L= 0.600 3 Note
PLAT941_ALERT_3_G Average HKL Measurement Multiplicity .... 4.4 Low
PLAT965_ALERT_2_G The SHELXL WEIGHT Optimisation has not Converged Please Check
PLAT969_ALERT_5_G The 'Henn et al.' R-Factor-gap value .... 2.175 Note
Predicted wR2: Based on SigI**2 5.20 or SHELX Weight 10.96
PLAT978_ALERT_2_G Number C-C Bonds with Positive Residual Density. 0 Info

---

0 **ALERT level A** = Most likely a serious problem - resolve or explain
0 **ALERT level B** = A potentially serious problem, consider carefully
4 **ALERT level C** = Check. Ensure it is not caused by an omission or oversight

9 **ALERT level G** = General information/check it is not something unexpected

2 ALERT type 1 CIF construction/syntax error, inconsistent or missing data
4 ALERT type 2 Indicator that the structure model may be wrong or deficient
4 ALERT type 3 Indicator that the structure quality may be low
2 ALERT type 4 Improvement, methodology, query or suggestion
1 ALERT type 5 Informative message, check

PLATON version of 15/07/2024; check.def file version of 15/07/2024

Datablock SbMe3-TDCO2 - ellipsoid plot

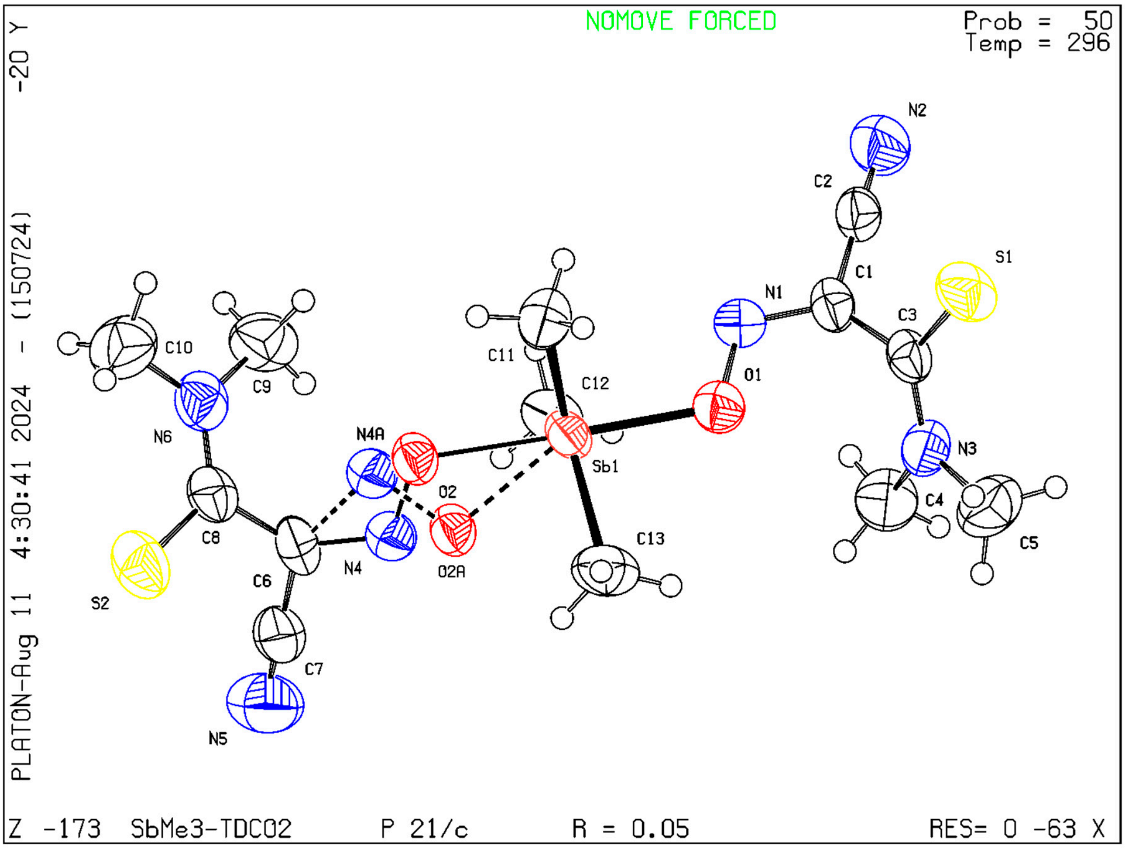

Download CIF editor (publCIF) from the IUCr
Download CIF editor (enCIFer) from the CCDC
Test a new CIF entry

CheckCIF reports for <u>*auxiliary structures*</u> of some starting complexes of Tl(I) with cyanoximes

checkCIF/PLATON (basic structural check)

Structure factors have been supplied for datablock(s) Tl-4Cl-PhCO

No syntax errors found. CIF dictionary
Please wait while processing .... Interpreting this report
Structure factor report

Datablock: Tl-4Cl-PhCO

Bond precision:	C-C = 0.0086 A		Wavelength=0.71073
Cell:	a=10.951(4)	b=4.0853(15)	c=20.616(7)
	alpha=90	beta=99.041(4)	gamma=90
Temperature:	296 K		

	Calculated	Reported
Volume	910.9(6)	910.9(6)
Space group	P 21/n	P 21/n
Hall group	-P 2yn	-P 2yn
Moiety formula	C8 H4 Cl N2 O Tl	C8 H4 Cl N2 O Tl
Sum formula	C8 H4 Cl N2 O Tl	C8 H4 Cl N2 O Tl
Mr	383.96	383.95
Dx,g cm-3	2.800	2.800
Z	4	4
Mu (mm-1)	17.980	17.980
F000	688.0	688.0
F000'	678.29	
h,k,lmax	14,5,28	14,5,28
Nref	2407	2391
Tmin,Tmax	0.346,0.478	0.211,0.437
Tmin'	0.001	

Correction method= # Reported T Limits:
Tmin=0.211 Tmax=0.437 AbsCorr = MULTI-SCAN

Data completeness= 0.993 Theta(max)= 28.903

R(reflections)= 0.0362(1893)	wR2(reflections)= 0.0892(2391)
S = 1.025	Npar= 130

The following ALERTS were generated. Each ALERT has the format
test-name_ALERT_alert-type_alert-level.
Click on the hyperlinks for more details of the test.

Alert level C

PLAT245_ALERT_2_C U(iso) H4 Smaller than U(eq) C4 by 0.019 Ang**2
PLAT245_ALERT_2_C U(iso) H7 Smaller than U(eq) C7 by 0.011 Ang**2
PLAT342_ALERT_3_C Low Bond Precision on C-C Bonds .... 0.00862 Ang.
PLAT911_ALERT_3_C Missing FCF Refl Between Thmin & STh/L= 0.600 9 Report
-11 2 1, 1 0 1, -9 0 3, -1 0 3, -10 0 4, -11 0 5,
-12 0 6, 4 3 9, 4 0 22,
PLAT971_ALERT_2_C Check Calcd Resid. Dens. 0.84Ang From Tl01 1.73 eA-3
PLAT971_ALERT_2_C Check Calcd Resid. Dens. 0.87Ang From Tl01 1.63 eA-3
PLAT972_ALERT_2_C Check Calcd Resid. Dens. 0.96Ang From Tl01 -2.00 eA-3
PLAT972_ALERT_2_C Check Calcd Resid. Dens. 0.79Ang From Tl01 -1.83 eA-3
PLAT976_ALERT_2_C Check Calcd Resid. Dens. 0.95Ang From O1 . -0.56 eA-3

Alert level G

PLAT004_ALERT_5_G Polymeric Structure Found with Maximum Dimension 1 Info
PLAT164_ALERT_4_G Nr. of Refined C-H H-Atoms in Heavy-Atom Struct. 3 Note
PLAT232_ALERT_2_G Hirshfeld Test Diff (M-X) Tl01 --O1_b . 6.5 s.u.
PLAT434_ALERT_2_G Short Inter HL..HL Contact Cl1 ..Cl1 . 3.36 Ang.
-x,-y,1-z = 3_556 Check
PLAT720_ALERT_4_G Number of Unusual/Non-Standard Labels .... 1 Note
Tl01
PLAT883_ALERT_1_G No Info/Value for _atom_sites_solution_primary . Please Do !
PLAT910_ALERT_3_G Missing # of FCF Reflection(s) Below Theta(Min). 1 Note
-1 0 1,
PLAT912_ALERT_4_G Missing # of FCF Reflections Above STh/L= 0.600 6 Note
PLAT933_ALERT_2_G Number of HKL-OMIT Records in Embedded .res File 7 Note
-11 0 5, -10 0 4, -9 0 3, -1 0 1, 4 0 22, 1 0 1,
4 3 9,
PLAT941_ALERT_3_G Average HKL Measurement Multiplicity .... 4.4 Low
PLAT969_ALERT_5_G The 'Henn et al.' R-Factor-gap value .... 1.785 Note
Predicted wR2: Based on SigI**2 4.99 or SHELX Weight 8.70
PLAT978_ALERT_2_G Number C-C Bonds with Positive Residual Density. 1 Info

0 **ALERT level A** = Most likely a serious problem - resolve or explain
0 **ALERT level B** = A potentially serious problem, consider carefully
9 **ALERT level C** = Check. Ensure it is not caused by an omission or oversight
12 **ALERT level G** = General information/check it is not something unexpected

1 ALERT type 1 CIF construction/syntax error, inconsistent or missing data
11 ALERT type 2 Indicator that the structure model may be wrong or deficient
4 ALERT type 3 Indicator that the structure quality may be low
3 ALERT type 4 Improvement, methodology, query or suggestion
2 ALERT type 5 Informative message, check

PLATON version of 15/07/2024; check.def file version of 15/07/2024

Datablock Tl-4Cl-PhCO - ellipsoid plot

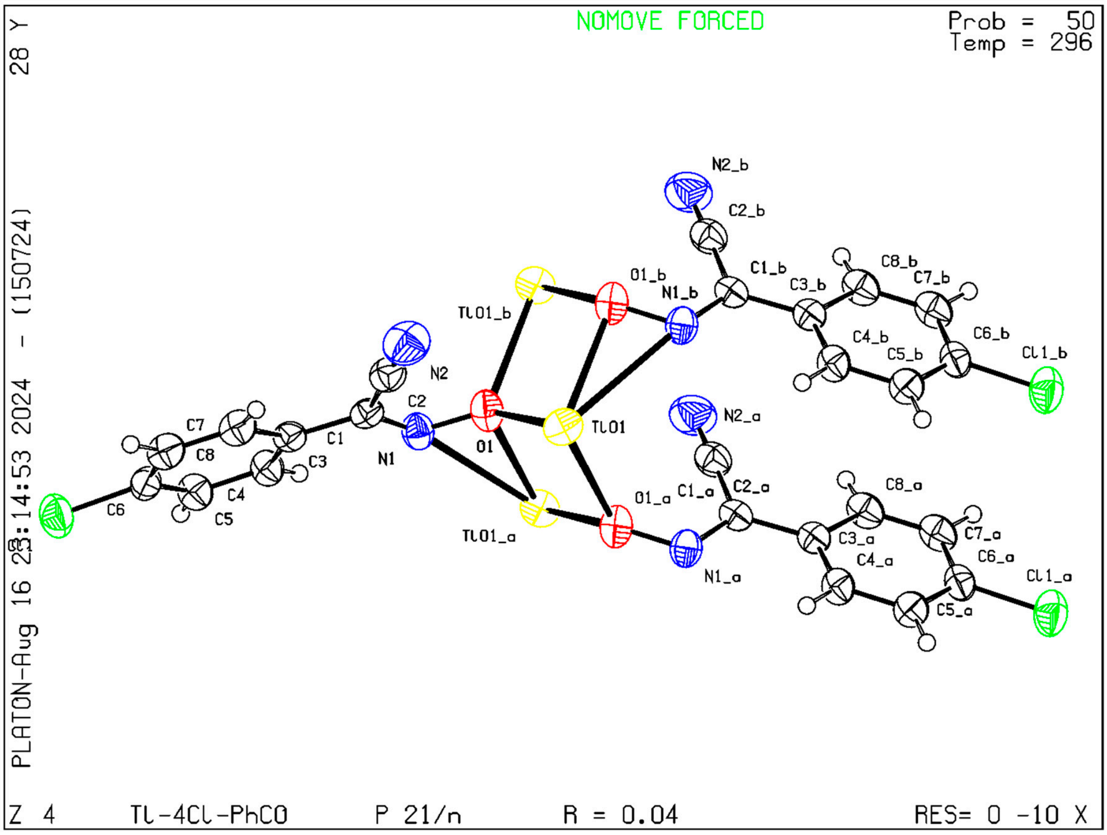

Download CIF editor (publCIF) from the IUCr
Download CIF editor (enCIFer) from the CCDC
Test a new CIF entry

checkCIF/PLATON (basic structural check)

Structure factors have been supplied for datablock(s) Tl_2-4-diCl-PhCO

No syntax errors found. CIF dictionary
Please wait while processing .... Interpreting this report
Structure factor report

Datablock: Tl_2-4-diCl-PhCO

```
Bond precision:  C-C = 0.0182 A          Wavelength=0.71073
```

Cell:	a=37.024(9)	b=3.8702(10)	c=14.628(4)
	alpha=90	beta=107.073(3)	gamma=90
Temperature:	273 K		

	Calculated	Reported
Volume	2003.7(9)	2003.7(9)
Space group	C 2/c	C 2/c
Hall group	-C 2yc	-C 2yc
Moiety formula	C8 H3 Cl2 N2 O Tl	?
Sum formula	C8 H3 Cl2 N2 O Tl	C8 H3 Cl2 N2 O Tl
Mr	418.40	418.39
Dx,g cm-3	2.774	2.774
Z	8	8
Mu (mm-1)	16.618	16.618
F000	1504.0	1504.0
F000'	1485.64	
h,k,lmax	56,5,22	54,5,21
Nref	3698	3444
Tmin,Tmax	0.131,0.206	0.326,0.747
Tmin'	0.040	

Correction method= # Reported T Limits: Tmin=0.326 Tmax=0.747 AbsCorr = MULTI-SCAN

Data completeness= 0.931 Theta(max)= 32.756

R(reflections)= 0.0596(2589) wR2(reflections)= 0.1753(3444)

S = 1.199 Npar= 127

The following ALERTS were generated. Each ALERT has the format
test-name_ALERT_alert-type_alert-level.
Click on the hyperlinks for more details of the test.

Alert level B

PLAT972_ALERT_2_B Check Calcd Resid. Dens. 1.00Ang From C2 -2.76 eA-3

Alert level C

PLAT234_ALERT_4_C Large Hirshfeld Difference N2 --C2 . 0.16 Ang.
PLAT242_ALERT_2_C Low 'MainMol' Ueq as Compared to Neighbors of C2 Check
PLAT342_ALERT_3_C Low Bond Precision on C-C Bonds .... 0.01825 Ang.
PLAT906_ALERT_3_C Large K Value in the Analysis of Variance ...... 7.800 Check
PLAT911_ALERT_3_C Missing FCF Refl Between Thmin & STh/L= 0.600 9 Report
42 0 0, -42 0 2, -32 2 2, 10 0 2, 28 0 6, -27 1 10,
-22 0 10, -29 1 11, -14 0 16,
PLAT918_ALERT_3_C Reflection(s) with I(obs) much Smaller I(calc) . 1 Check
PLAT934_ALERT_3_C Number of (Iobs-Icalc)/Sigma(W) > 10 Outliers .. 1 Check
-26 0 12,
PLAT971_ALERT_2_C Check Calcd Resid. Dens. 0.81Ang From Tl1 1.77 eA-3
And 5 other PLAT971 Alerts
More ...
PLAT972_ALERT_2_C Check Calcd Resid. Dens. 0.72Ang From Tl1 -2.07 eA-3
PLAT972_ALERT_2_C Check Calcd Resid. Dens. 1.40Ang From Tl1 -1.63 eA-3

PLAT975_ALERT_2_C Check Calcd Resid. Dens. 0.97Ang From N1 . 1.37 eA-3

Alert level G

PLAT004_ALERT_5_G Polymeric Structure Found with Maximum Dimension 1 Info
PLAT083_ALERT_2_G SHELXL Second Parameter in WGHT Unusually Large 84.28 Why ?
PLAT128_ALERT_4_G Alternate Setting for Input Space Group C2/c I2/a Note
PLAT199_ALERT_1_G Reported _cell_measurement_temperature ..... (K) 273 Check
PLAT200_ALERT_1_G Reported _diffrn_ambient_temperature ..... (K) 273 Check
PLAT794_ALERT_5_G Tentative Bond Valency for Tl1 (I) . 0.68 Info
PLAT883_ALERT_1_G No Info/Value for _atom_sites_solution_primary . Please Do !
PLAT899_ALERT_4_G SHELXL2018 is Deprecated and Succeeded by SHELXL 2019/3 Note
PLAT910_ALERT_3_G Missing # of FCF Reflection(s) Below Theta(Min). 1 Note
2 0 0,
PLAT912_ALERT_4_G Missing # of FCF Reflections Above STh/L= 0.600 227 Note
PLAT941_ALERT_3_G Average HKL Measurement Multiplicity .... 3.1 Low
PLAT950_ALERT_5_G Calculated (ThMax) and CIF-Reported Hmax Differ 2 Units
PLAT956_ALERT_1_G Calculated (ThMax) and Actual (FCF) Hmax Differ 2 Units
PLAT965_ALERT_2_G The SHELXL WEIGHT Optimisation has not Converged Please Check
PLAT969_ALERT_5_G The 'Henn et al.' R-Factor-gap value .... 3.329 Note
Predicted wR2: Based on SigI**2 5.27 or SHELX Weight 14.62
PLAT978_ALERT_2_G Number C-C Bonds with Positive Residual Density. 0 Info

0 **ALERT level A** = Most likely a serious problem - resolve or explain
1 **ALERT level B** = A potentially serious problem, consider carefully
16 **ALERT level C** = Check. Ensure it is not caused by an omission or oversight
16 **ALERT level G** = General information/check it is not something unexpected

4 ALERT type 1 CIF construction/syntax error, inconsistent or missing data
14 ALERT type 2 Indicator that the structure model may be wrong or deficient
7 ALERT type 3 Indicator that the structure quality may be low
4 ALERT type 4 Improvement, methodology, query or suggestion
4 ALERT type 5 Informative message, check

PLATON version of 15/07/2024; check.def file version of 15/07/2024

Datablock Tl_2-4-diCl-PhCO - ellipsoid plot

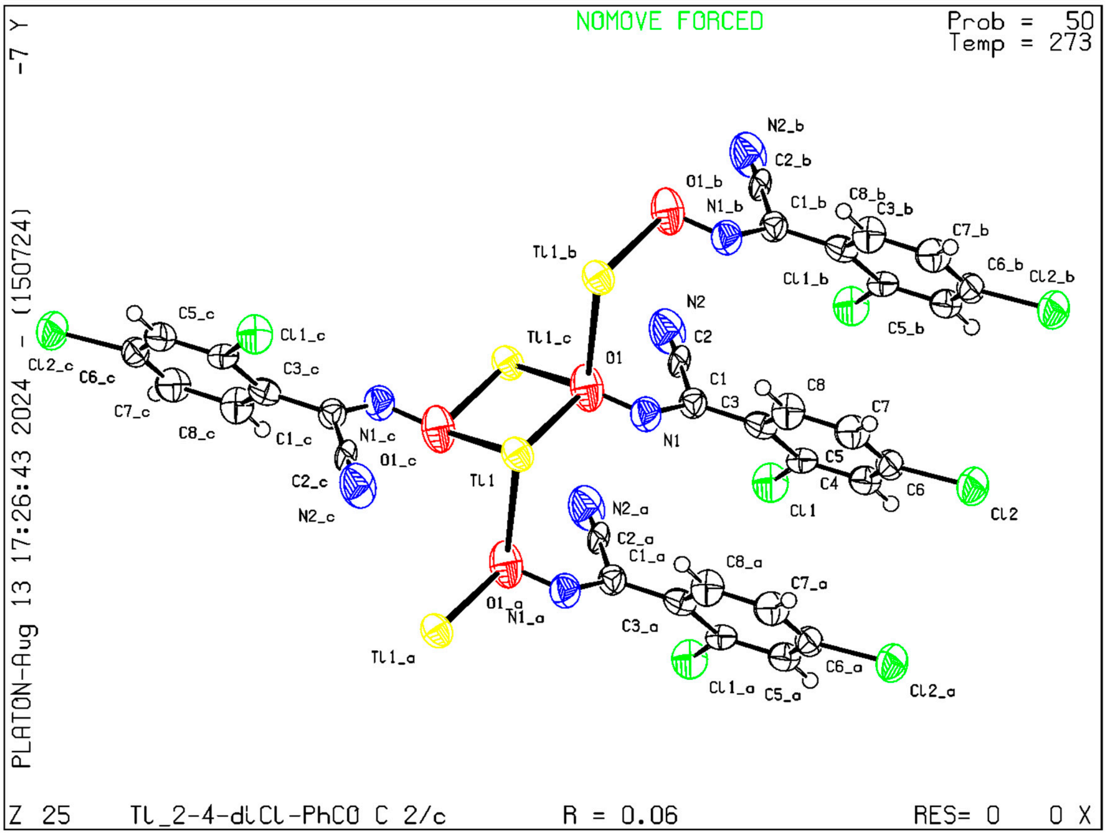

Download CIF editor (publCIF) from the IUCr
Download CIF editor (enCIFer) from the CCDC
Test a new CIF entry

checkCIF/PLATON (basic structural check)

Structure factors have been supplied for datablock(s) Tl-2-6-diCl-PhCO

THIS REPORT IS FOR GUIDANCE ONLY. IF USED AS PART OF A REVIEW PROCEDURE FOR PUBLICATION, IT SHOULD NOT REPLACE THE EXPERTISE OF AN EXPERIENCED CRYSTALLOGRAPHIC REFEREE.

No syntax errors found. CIF dictionary
Please wait while processing .... Interpreting this report
Structure factor report

Datablock: Tl-2-6-diCl-PhCO

Bond precision:	C-C = 0.0325 A		Wavelength=0.71073
Cell:	a=4.070(12)	b=7.73(2)	c=31.04(9)
	alpha=90	beta=91.01(5)	gamma=90
Temperature: 150 K			

	Calculated	Reported
Volume	976(5)	977(5)
Space group	P 21/n	P 21/n
Hall group	-P 2yn	-P 2yn
Moiety formula	C8 H3 Cl2 N2 O Tl	?
Sum formula	C8 H3 Cl2 N2 O Tl	C8 H4 Cl2 N2 O Tl
Mr	418.40	419.40
Dx,g cm-3	2.848	2.852
Z	4	4
Mu (mm-1)	17.058	17.047
F000	752.0	756.0
F000'	742.82	
h,k,lmax	5,11,44	5,10,33
Nref	2975	2440
Tmin,Tmax		0.348,0.549
Tmin'		

Correction method= # Reported T Limits: Tmin=0.348 Tmax=0.549 AbsCorr = MULTI-SCAN

Data completeness= 0.820 Theta(max)= 30.515

R(reflections)= 0.0796(1275) wR2(reflections)= 0.2004(2440)

S = 0.921 Npar= 122

The following ALERTS were generated. Each ALERT has the format
test-name_ALERT_alert-type_alert-level.
Click on the hyperlinks for more details of the test.

Alert level A

PLAT029_ALERT_3_A _diffrn_measured_fraction_theta_full value Low . 0.896 Why?
PLAT971_ALERT_2_A Check Calcd Resid. Dens. 1.00Ang From Tl01 3.90 eA-3
PLAT971_ALERT_2_A Check Calcd Resid. Dens. 0.95Ang From Tl01 3.84 eA-3
PLAT973_ALERT_2_A Check Calcd Positive Resid. Density on Tl01 3.09 eA-3

Alert level B

PLAT342_ALERT_3_B Low Bond Precision on C-C Bonds .... 0.0325 Ang.
PLAT971_ALERT_2_B Check Calcd Resid. Dens. 0.97Ang From Tl01 3.46 eA-3
And 5 other PLAT971 Alerts
More ...
PLAT972_ALERT_2_B Check Calcd Resid. Dens. 0.91Ang From Tl01 -3.29 eA-3
PLAT972_ALERT_2_B Check Calcd Resid. Dens. 0.88Ang From Tl01 -2.65 eA-3

Alert level C

PLAT041_ALERT_1_C Calc. and Reported SumFormula Strings Differ Please Check
Calc: C8 H3 Cl2 N2 O Tl

Rep.: C8 H4 Cl2 N2 O Tl
PLAT043_ALERT_1_C Calculated and Reported Mol. Weight Differ by .. 1.00 Check
PLAT053_ALERT_1_C Minimum Crystal Dimension Missing (or Error) ... Please Check
PLAT054_ALERT_1_C Medium Crystal Dimension Missing (or Error) ... Please Check
PLAT055_ALERT_1_C Maximum Crystal Dimension Missing (or Error) ... Please Check
PLAT068_ALERT_1_C Reported F000 Differs from Calcd (or Missing)... Please Check
PLAT148_ALERT_3_C s.u. on the a - Axis is (Too) Large .... 0.012 Ang.
And 2 other PLAT148 Alerts
More ...
PLAT369_ALERT_2_C Long C(sp2)-C(sp2) Bond C1 - C3 . 1.54 Ang.
PLAT906_ALERT_3_C Large K Value in the Analysis of Variance ...... 11.667 Check
PLAT906_ALERT_3_C Large K Value in the Analysis of Variance ...... 2.648 Check
PLAT911_ALERT_3_C Missing FCF Refl Between Thmin & STh/L= 0.600 156 Report
2 5 0, 1 1 1, 1 6 1, 0 7 2, -1 5 3, 3 1 4,
1 0 5, 3 1 6, 0 3 6, -1 0 7, 1 0 7, -2 2 10,
-2 0 14, -3 0 19, -1 2 19, 0 0 20, -1 2 20, -1 2 21,
-3 0 23, -1 0 23, -2 0 24, 0 0 24, -3 1 24, -3 0 25,
-1 0 25, 1 0 25, -3 1 25, -2 1 25, -1 1 25, -3 2 25,
-2 0 26, 0 0 26, -3 1 26, -2 1 26, -1 1 26, 0 1 26,
-3 2 26, -2 2 26, -3 0 27, -1 0 27, 1 0 27, -3 1 27,
-2 1 27, -1 1 27, 0 1 27, 1 1 27, -3 2 27, -2 2 27,
-1 2 27, -3 3 27, -2 3 27, -1 3 27, -2 0 28, 0 0 28,
2 0 28, -3 1 28, -2 1 28, -1 1 28, 0 1 28, 1 1 28,
-3 2 28, -2 2 28, -1 2 28, 0 2 28, -2 3 28, -1 3 28,
-3 0 29, -1 0 29, 1 0 29, -3 1 29, -2 1 29, -1 1 29,
0 1 29, 1 1 29, 2 1 29, -2 2 29, -1 2 29, 0 2 29,
1 2 29, -2 3 29, -1 3 29, 0 3 29, -2 4 29, -2 0 30,
0 0 30, 2 0 30, -2 1 30, -1 1 30, 0 1 30, 1 1 30,
2 1 30, -2 2 30, -1 2 30, 0 2 30, 1 2 30, 2 2 30,
PLAT971_ALERT_2_C Check Calcd Resid. Dens. 1.03Ang From Tl01 2.11 eA-3
And 5 other PLAT971 Alerts
More ...
PLAT972_ALERT_2_C Check Calcd Resid. Dens. 1.09Ang From Tl01 -2.37 eA-3
And 15 other PLAT972 Alerts
More ...
PLAT977_ALERT_2_C Check Negative Difference Density on H6 . -0.71 eA-3

Alert level G

FORMU01_ALERT_2_G There is a discrepancy between the atom counts in the
_chemical_formula_sum and the formula from the _atom_site* data.
Atom count from _chemical_formula_sum:C8 H4 Cl2 N2 O1 Tl1
Atom count from the _atom_site data: C8 H3 Cl2 N2 O1 Tl1
CELLZ01_ALERT_1_G Difference between formula and atom_site contents detected.
CELLZ01_ALERT_1_G WARNING: H atoms missing from atom site list. Is this intentional?
From the CIF: _cell_formula_units_Z 4
From the CIF: _chemical_formula_sum C8 H4 Cl2 N2 O Tl
TEST: Compare cell contents of formula and atom_site data

atom	Z*formula	cif sites	diff
C	32.00	32.00	0.00
H	16.00	12.00	4.00
Cl	8.00	8.00	0.00
N	8.00	8.00	0.00

O 4.00 4.00 0.00
Tl 4.00 4.00 0.00

PLAT003_ALERT_2_G Number of Uiso or U(i,j) Restrained non-H Atoms 14 Report
PLAT004_ALERT_5_G Polymeric Structure Found with Maximum Dimension 1 Info
PLAT171_ALERT_4_G The CIF-Embedded .res File Contains EADP Records 1 Report
PLAT177_ALERT_4_G The CIF-Embedded .res File Contains DELU Records 1 Report
PLAT178_ALERT_4_G The CIF-Embedded .res File Contains SIMU Records 1 Report
PLAT188_ALERT_3_G A Non-default SIMU Restraint Value has been used 0.0200 Report
PLAT192_ALERT_3_G A Non-default DELU Restraint Value for First Par 0.0020 Report
PLAT720_ALERT_4_G Number of Unusual/Non-Standard Labels .... 1 Note
Tl01
PLAT794_ALERT_5_G Tentative Bond Valency for Tl01 (I) . 0.70 Info
PLAT860_ALERT_3_G Number of Least-Squares Restraints .... 110 Note
PLAT883_ALERT_1_G No Info/Value for _atom_sites_solution_primary . Please Do !
PLAT910_ALERT_3_G Missing # of FCF Reflection(s) Below Theta(Min). 1 Note
0 0 2,
PLAT912_ALERT_4_G Missing # of FCF Reflections Above STh/L= 0.600 128 Note
PLAT913_ALERT_3_G Missing # of Very Strong Reflections in FCF .... 3 Note
1 1 1, -1 0 7, 1 0 7,
PLAT933_ALERT_2_G Number of HKL-OMIT Records in Embedded .res File 12 Note
-1 0 7, -3 0 19, -2 3 27, 1 1 1, 1 0 7, 3 1 6,
-1 5 3, 2 5 0, 1 0 5, 3 1 4, 1 6 1, 0 7 2,
PLAT941_ALERT_3_G Average HKL Measurement Multiplicity .... 1.6 Low
PLAT952_ALERT_5_G Calculated (ThMax) and CIF-Reported Lmax Differ. 11 Units
PLAT958_ALERT_1_G Calculated (ThMax) and Actual (FCF) Lmax Differ. 11 Units
PLAT969_ALERT_5_G The 'Henn et al.' R-Factor-gap value .... 1.632 Note
Predicted wR2: Based on SigI**2 12.28 or SHELX Weight 21.75
PLAT978_ALERT_2_G Number C-C Bonds with Positive Residual Density. 0 Info

---

4 **ALERT level A** = Most likely a serious problem - resolve or explain
9 **ALERT level B** = A potentially serious problem, consider carefully
36 **ALERT level C** = Check. Ensure it is not caused by an omission or oversight
23 **ALERT level G** = General information/check it is not something unexpected

10 ALERT type 1 CIF construction/syntax error, inconsistent or missing data
39 ALERT type 2 Indicator that the structure model may be wrong or deficient
14 ALERT type 3 Indicator that the structure quality may be low
5 ALERT type 4 Improvement, methodology, query or suggestion
4 ALERT type 5 Informative message, check

PLATON version of 22/08/2024; check.def file version of 21/08/2024

Datablock Tl-2-6-diCl-PhCO - ellipsoid plot

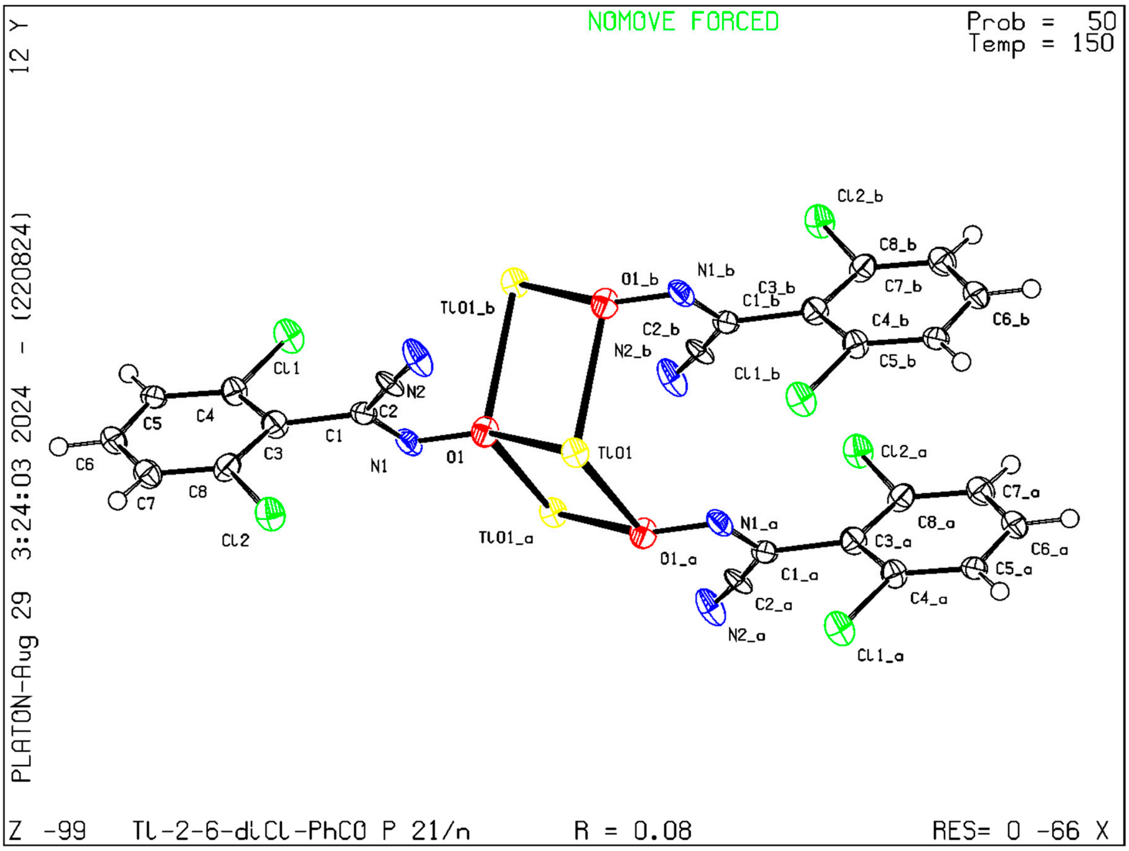

Download CIF editor (publCIF) from the IUCr
Download CIF editor (enCIFer) from the CCDC
Test a new CIF entry

SI 31

General sequence of procedures after the metathesis reaction during preparation of $Sb(CH_3)_3L_2$ with depiction of synthesis of the TCO^- derivative shown as an example.

$$SbMe_3Br_2 \; + \; 2\,ML \; = \; SbMe_3L_2 \; + \; MBr\downarrow \quad (M = Ag, Tl)$$

Filtration of very finely dispersed bromides of silver(I), which is light sensitive, and thallium(I) bromide is difficult. Using Celite© is not possible since its surface contains absorbed water and hydroxyl groups on the surface. Both cause hydrolysis and generate inconsistent products with poor analytical data. A solution to the problem is centrifugation (see next page).

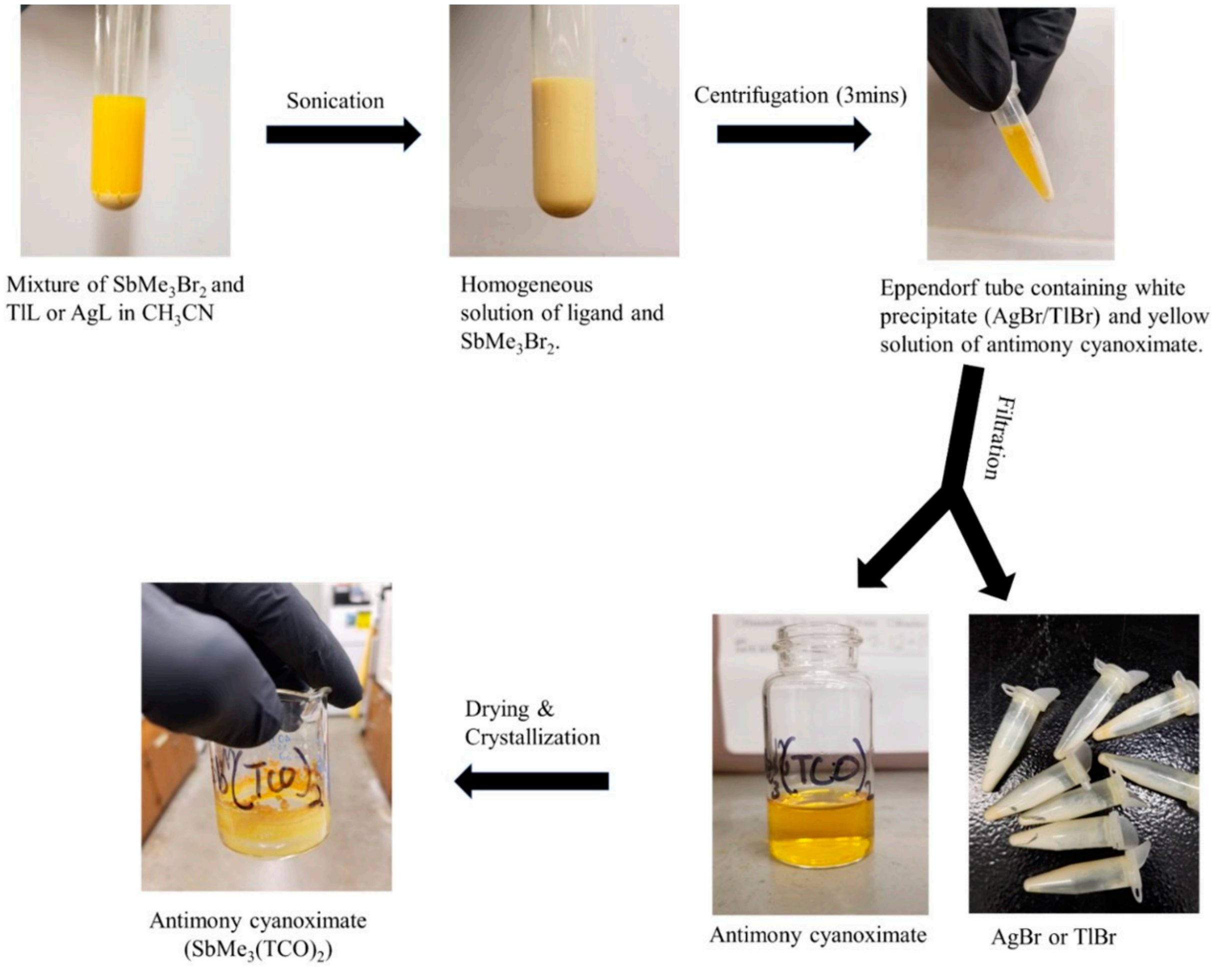

SI 32

The set of hardware used in the synthesis of organoantimony(V) compounds proved to be very convenient.

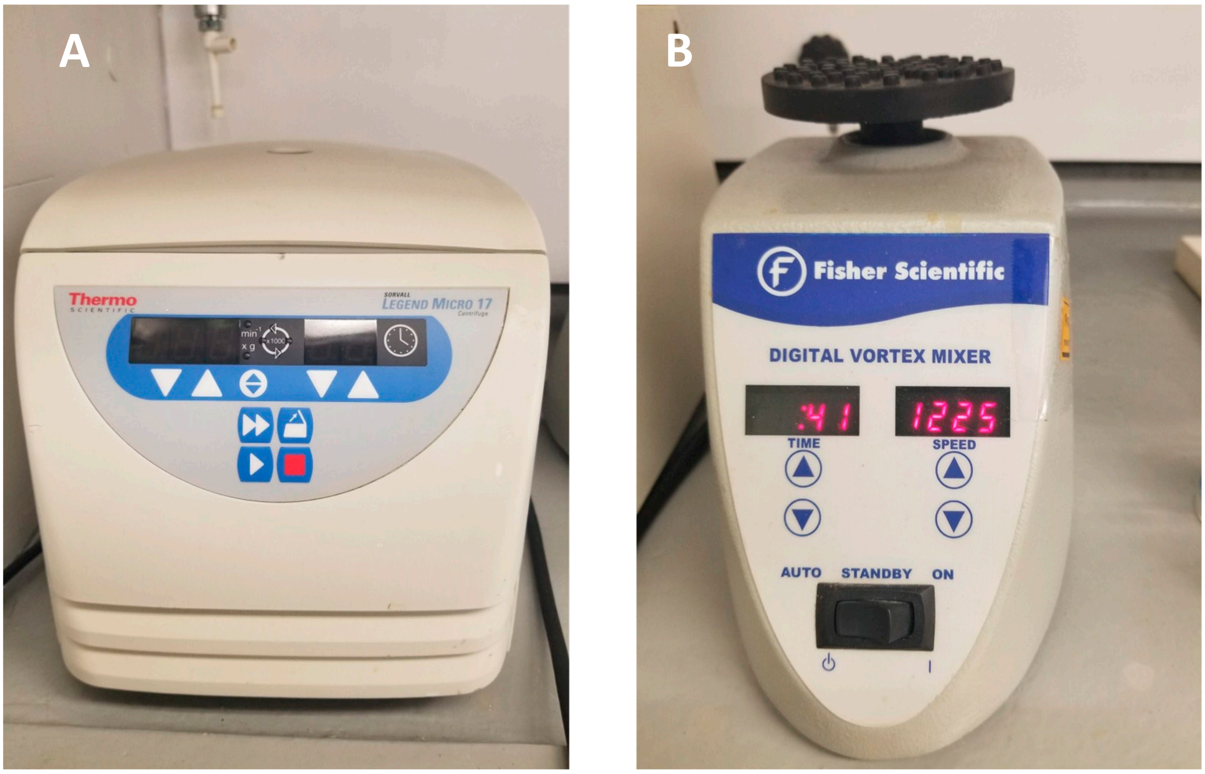

(A) Thermo Scientific Sorvall Legends Micro 17 centrifuge *<u>for separation of solids</u>* from solutions of target compounds in propionitrile.

(B) Fisher Scientific Vortex employed for the mixing of components in a heterogeneous reaction between ML (M = Ag, Tl; L = selected for studies cyanoxime) and $Sb(CH_3)_3Br_2$.
